# Non-canonical role for the BAF complex subunit DPF3 in mitosis and ciliogenesis

**DOI:** 10.1242/jcs.261744

**Published:** 2024-05-13

**Authors:** Giulia Verrillo, Anna Maria Obeid, Alexia Genco, Jacopo Scrofani, François Orange, Sarah Hanache, Julien Mignon, Tanguy Leyder, Catherine Michaux, Céline Kempeneers, Noëmie Bricmont, Stephanie Herkenne, Isabelle Vernos, Maud Martin, Denis Mottet

**Affiliations:** ^1^ University of Liege, GIGA – Research Institute, Molecular Analysis of Gene Expression (MAGE) Laboratory, B34, Avenue de l'Hôpital, B-4000 Liège, Belgium; ^2^ Centre for Genomic Regulation (CRG), The Barcelona Institute of Science and Technology, Barcelona 08003, Spain; ^3^ Université Côte d'Azur, Centre Commun de Microscopie Appliquée (CCMA), 06100 Nice, France; ^4^ University of Namur, Laboratory of Physical Chemistry of Biomolecules, Unité de Chimie Physique Théorique et Structurale (UCPTS), Rue de Bruxelles 61, B-5000 Namur, Belgium; ^5^ University of Liege, Pneumology Laboratory, I3 Group, GIGA Research Center, B-4000 Liège, Belgium; ^6^ Division of Respirology, Department of Pediatrics, University Hospital Liège, B-4000 Liège, Belgium; ^7^ University of Liege, GIGA-Cancer, Laboratory of Mitochondria and Cell Communication, B34, Avenue de l'Hôpital, B-4000 Liège, Belgium; ^8^ Universitat Pompeu Fabra (UPF), Barcelona 08002, Spain; ^9^ ICREA, Pg. Lluis Companys 23, Barcelona 08010, Spain; ^10^ Laboratory of Neurovascular Signaling, Department of Molecular Biology, ULB Neuroscience Institute, Université libre de Bruxelles, B-6041 Gosselies, Belgium

**Keywords:** DPF3, Chromosome alignment, Bridging fiber, Mitosis, Genomic instability, Centriolar satellites, Ciliogenesis

## Abstract

DPF3, along with other subunits, is a well-known component of the BAF chromatin remodeling complex, which plays a key role in regulating chromatin remodeling activity and gene expression. Here, we elucidated a non-canonical localization and role for DPF3. We showed that DPF3 dynamically localizes to the centriolar satellites in interphase and to the centrosome, spindle midzone and bridging fiber area, and midbodies during mitosis. Loss of DPF3 causes kinetochore fiber instability, unstable kinetochore–microtubule attachment and defects in chromosome alignment, resulting in altered mitotic progression, cell death and genomic instability. In addition, we also demonstrated that DPF3 localizes to centriolar satellites at the base of primary cilia and is required for ciliogenesis by regulating axoneme extension. Taken together, these findings uncover a moonlighting dual function for DPF3 during mitosis and ciliogenesis.

## Teaser

**Highlighted Article:** The BAF complex subunit DPF3 localizes to centriolar satellites in interphase and to the spindle midzone and the midbody during mitosis, and has dual functions in mitosis and primary ciliogenesis.

## INTRODUCTION

The Brg1- or Brm-associated factor (BAF) complex, the mammalian homolog of the yeast SWI/SNF complex, is one of the four mammalian ATP-dependent chromatin remodeling complex families (Ho and Crabtree, 2010; Kadoch and Crabtree, 2015). The core of the BAF complex is composed of an ATPase catalytic subunit [either Brg1 (also known as SMARCA4) or Brm (SMARCA2)] and at least BAF57 (SMARCE1), BAF53A (ACTL6A), BAF155 (SMARCC1), BAF170 (SMARCC2) and BAF47 (SMARCB1, homolog of yeast SNF5). These core subunits are responsible for essential chromatin remodeling activity (Yan et al., 2017). However, hundreds of distinct BAF complexes are predicted to form *in vivo* through combinatorial assembly (Ho and Crabtree, 2010). Based on the combination of additional non-core BAF subunits, BAF complexes have unique and specific composition in certain tissue or cell types to perform a specialized function.

Shifts in isoforms of non-core BAF complex subunits are important for the transition from a precursor to a differentiated state during neural and muscle differentiation. For instance, the neural precursor BAF complex (npBAF) in neural progenitor cells is composed of BAF53A and BAF45A (PHF10) (Lessard et al., 2007; Staahl and Crabtree, 2013). After mitosis and differentiation, these subunits are substituted by BAF53B (ACTL6B), BAF45B (DPF1) or BAF45C (DPF3) and form the neural BAF complexes (nBAF) (Lessard et al., 2007). Similarly, BAF45C is a specific component of the cardiac BAF complex (CBAF), which is essential for heart and muscle development (Han et al., 2011).

Even if there has been an enormous gain in understanding how BAF complexes assemble and function, the roles of distinct non-core subunits remain poorly understood. Despite its essential role in neural development, very little is known about the role and mechanisms of action of the auxiliary BAF45C subunit in other tissues or cell types.

In humans, BAF45C, also named double plant homeobox domain (PHD) fingers 3 (DPF3) or CERD4, exists as two isoforms: DPF3b and DPF3a. DPF3b is characterized by a tandem of PHD zinc fingers at its C-terminus extremity. These structural features enable DPF3b to bind acetylated or methylated histones on the chromatin, hence acting as a histone reader in the BAF complex (Lange et al., 2008; Zeng et al., 2010). Unlike DPF3b, DPF3a has a single truncated PHD zinc finger (PHD-1/2) and is therefore unable to bind modified histones. Nonetheless, DPF3a is involved in myogenic differentiation by interacting with the hepatoma-derived growth factor-related protein 2 (HRP2, also known as HDGFL2). Formation of DPF3a–HRP2 complex facilitates the association of HRP2 with BAF, leading to the transcription of myogenic genes (Zhu et al., 2020). Cardiac gene transcription has also been found to be regulated by DPF3a through phosphorylation of its C-terminal domain and interaction with transcriptional repressors of the HEY protein family (HES-related repressor proteins). The BAF complex is thereafter recruited by the DPF3a–HEY complex, triggering the transcription of fetal genes, which promotes cardiac hypertrophy (Cui et al., 2016).

Both DPF3a and DPF3b are intrinsically disordered proteins (IDPs) (Mignon et al., 2021, 2022; Leyder et al., 2022). The two isoforms have a high disorder content, lack a hydrophobic core and display a context-dependent expanded and/or collapsed conformational ensemble. DPF3a is more disordered than DPF3b owing to the presence of an additional intrinsically disordered region (IDR) at its C-terminus. The flexibility of IDPs is exploited in their biological function and, in many instances, allows a single IDP to interact with multiple proteins and regulate a broad range of biological processes. Our recent characterization of the disordered nature of DPF3 isoforms is highly pertinent (Mignon et al., 2021, 2022; Leyder et al., 2022) and drives us for a better understanding of its functionality. We therefore aimed to determine whether DPF3 exerts unexpected roles beyond its well-established function in regulating the BAF complex.

In our present study, the DPF3 protein was surprisingly found to dissociate from the BAF chromatin remodeling complex during mitosis and to play an unexpected direct role in cell division. DPF3 was spatiotemporally located into three distinct complex structures, including the centriolar satellites, the spindle midzone and bridging fiber area, as well as the midbody. Looking at the cellular importance of DPF3, we found that depletion of DPF3 induced cell cycle blocking in the M phase and subsequent apoptotic cell death, suggesting that localization of DPF3 to mitotic structures is essential for proper mitotic division and genomic stability. Mechanistically, we demonstrated that DPF3 depletion causes kinetochore fiber (K-fiber) instability, less stable kinetochore–microtubule (KT–MT) attachment and defects in chromosome alignment at the metaphase plate. Furthermore, we unveiled the localization of DPF3 at the basal body of the primary cilium in quiescent vertebrate cells. Depletion of DPF3 dramatically arrested primary ciliogenesis at the initial step of axoneme extension. Altogether, the results uncovered a previously unknown dual role for DPF3 in mitosis and ciliogenesis.

## RESULTS

### DPF3 is a centriolar satellite-associated protein

**Fig. 1.**
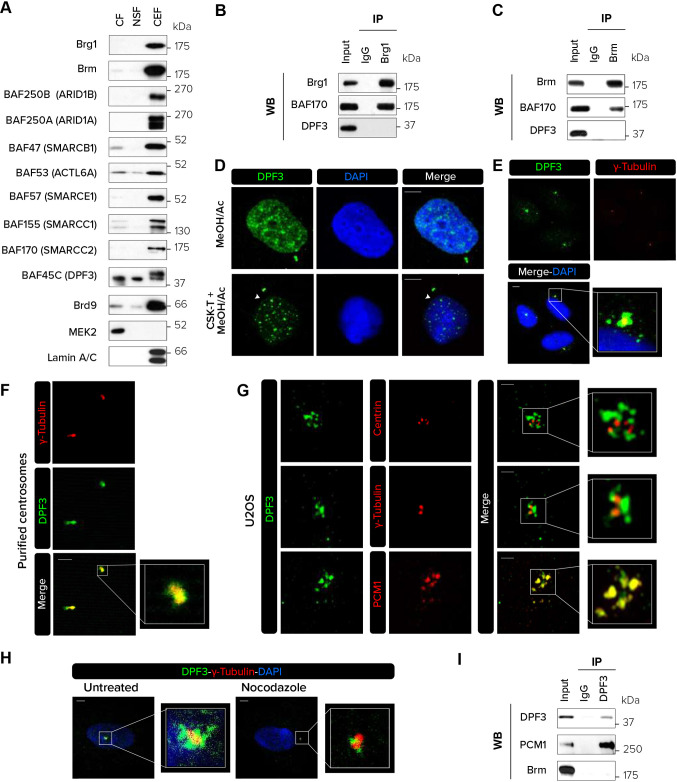
**DPF3 localizes to centriolar satellites in interphase.** (A) The cytosolic fraction (CF), nuclear soluble fraction (NSF) and chromatin-enriched fraction (CEF) were isolated from U2OS cells and analyzed by western blotting for the indicated proteins. (B,C) Immunoprecipitation (IP) of endogenous Brg1 (B) or Brm (C) from U2OS cells followed by western blotting (WB) for Brg1, Brm, BAF170 and DPF3. (D) U2OS cells were pre-extracted or not with CSK buffer containing Triton X-100 (CSK-T), fixed with methanol and acetone (80:20) and stained for DPF3. Individual channels and merged channels are shown. White arrowheads indicate centrosomes. (E,F) Co-staining for DPF3 (in green) and γ-tubulin (in red) in U2OS cells (E) or isolated centrosomes purified from KE37 cells (F). Individual channels, merged channels and magnifications of boxed regions are shown. (G) U2OS cells were co-stained for DPF3 (in green) and centrin, γ-tubulin or PCM1 (in red). Images were captured with Zeiss LSM 880 Airyscan high-resolution microscope. Individual channels, merged channels and magnifications of boxed regions are shown. (H) U2OS cells were treated with nocodazole (5 μg/ml) for 2 h to depolymerize the microtubule network. Cells were co-stained for DPF3 (in green) and γ-tubulin (in red). Merged channels with DAPI nuclear staining (in blue) and magnifications of boxed regions are shown. (I) Immunoprecipitation (IP) of endogenous DPF3 from U2OS cells followed by western blotting (WB) for DPF3, PCM1 and Brm. Western blotting and immunofluorescence images are representative of at least three independent experiments. Scale bars: 5 µm (D,E,H); 2 µm (F,G).

To identify the subcellular localization of DPF3, we first looked at its presence alongside individual BAF subunits in U2OS cells using chromatin-enriched fractionation. Although most of the subunits were predominantly present in the chromatin-enriched fraction, DPF3 was observed to be equally distributed in the cytoplasm and nuclear fraction (Fig. 1A). Two bands were observed in the chromatin-enriched fraction. As our DPF3 antibodies recognize both the DPF3a and DPF3b isoforms, we hypothesized that the lower band corresponds to the DPF3a isoform according to its theoretical molecular mass (38 kDa) and the upper band to DPF3b isoform with a molecular mass of 42 kDa. On the contrary, only the lower band was observed in the cytoplasmic and soluble nuclear fractions, suggesting that only DPF3a is present in these cellular compartments in this cell type.

To evaluate whether DPF3 interacts with the core ATPase BAF subunits Brg1 or Brm in U2OS cells, we performed co-immunoprecipitation (co-IP) experiments with antibodies against Brg1 or Brm (Fig. 1B,C). We found that although the core subunit BAF170 was physically associated with Brg1 and Brm, DPF3 was not present in the ATPase Brg1- or Brm-based complexes, suggesting that, at least in U2OS cells, DPF3 is not associated with the ATPase BAF subunits and might act independently.

In the light of the detected presence of DPF3 in the cytoplasmic fraction, we investigated the subcellular localization of endogenous DPF3 by immunofluorescence after fixation with cold methanol and acetone and staining with an antibody recognizing both DPF3a and DPF3b isoforms. In interphase, DPF3 was detected in the nuclear compartment (Fig. 1D; Fig. S1A). Surprisingly, a DPF3 staining pattern observed as small granules reminiscent of centrosome structure was also observed in the cytoplasm. To better visualize centrosomal localization of endogenous DPF3, cells were pre-incubated with cytoskeleton (CSK) extraction buffer prior to fixation with acetone and methanol to remove soluble proteins and enable detection of the insoluble proteins that are anchored to cellular structures. Immunofluorescence microscopy revealed DPF3 staining at centrosome-like structures, as well as bright foci in the nuclear compartment in interphasic cells (Fig. 1D). To validate the presence of DPF3 in the centrosomal compartment, endogenous DPF3 was co-stained with the common centrosome marker γ-tubulin. Confocal microscopy analysis revealed that DPF3 colocalized with γ-tubulin, confirming that DPF3 is indeed present in the centrosomal compartment (Fig. 1E). As an additional way to confirm the presence of endogenous DPF3 at centrosomes, we also performed immunofluorescence on purified centrosomes. The majority of centrosomes co-reacted with anti-DPF3 and anti-γ-tubulin antibodies, confirming the presence of DPF3 in centrosomes (Fig. 1F). Transfection of two different siRNAs (siDPF3 #1 and siDPF3 #2) targeting both the DPF3a and DPF3b isoforms significantly reduced DPF3 expression at both the mRNA and protein levels (Fig. S1B,C), and abrogated DPF3 staining in the vicinity of the centrosome (Fig. S1D). This result validates the specificity of the anti-DPF3 antibody used in immunofluorescence experiments. Interestingly, no centrosomal signal was detected for Brm or Brg1 and these were exclusively nuclear (Fig. S1E,F), suggesting that centrosomal localization of DPF3 was independent of the core BAF complex. In addition, DPF3 showed centrosomal localization in several other cell lines including HeLa, MDA-MB-231, T47D and MCF7 cells (Fig. S1G).

Centrosomes are composed of a pair of centrioles surrounded by amorphous pericentriolar material and small membrane-less granules called centriolar satellites. To more precisely determine the localization of DPF3 in these structures, immunofluorescence experiments were performed in both U2OS and HeLa cells using antibodies against centrin (CETN2), γ-tubulin or PCM1 to respectively label the centrioles (Salisbury et al., 2002), the pericentriolar matrix (Baumann, 2012) and the centriolar satellites (Hall et al., 2023). High-resolution microscopy analyses showed no colocalization of DPF3 with centrin (Fig. 1G; Fig. S2A,B), suggesting that DPF3 is not present in centrioles. A very faint colocalization with γ-tubulin was seen by high-resolution microscopy, suggesting that DPF3 is not highly abundant in the pericentriolar matrix. Remarkably, DPF3 and PCM1 colocalized almost perfectly, leading us to conclude that DPF3 is strictly a centriolar satellite protein.

The pericentrosomal distribution of centriolar satellite proteins depends on an intact microtubule network (Conkar et al., 2019). The disruption of the microtubule network by nocodazole treatment led to a substantial decrease in the amount of the granular DPF3 staining around the centrosome (Fig. 1H). This observation suggests that the major fraction of DPF3 localizes to centriolar satellites. Finally, co-IP experiments revealed that DPF3 interacts with PCM1 (Fig. 1I), further supporting DPF3 as a centriolar satellite protein.

### DPF3 is located in the centrosomal compartment, spindle midzones and bridging fiber area, and midbodies during mitosis

**Fig. 2.**
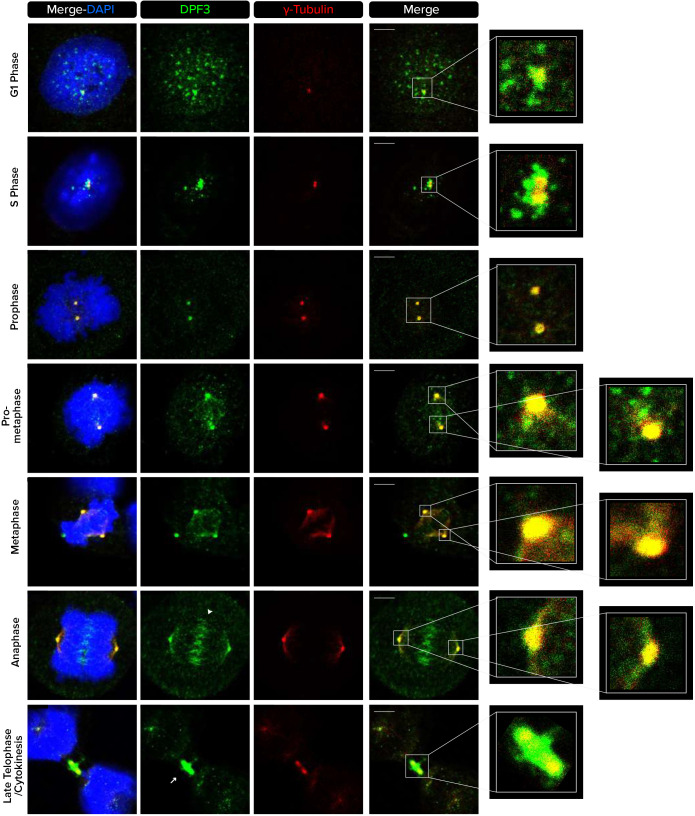
**DPF3 localizes to centrosomes and microtubule-based structures during mitotic cell division.** U2OS cells were co-stained for DPF3 (in green) and γ-tubulin (in red). Individual channels, merged channels with DAPI nuclear staining (in blue), and magnifications of boxed regions are shown. Nuclear condensation and the position of γ-tubulin foci identify cells in different cell cycle stages. The white arrowhead indicates the position of the spindle midzone in anaphase. The white arrow indicates the position of the midbody during cytokinesis. Images are representative of at least three independent experiments. Scale bars: 5 µm.

The centrosome is a dynamic structure that plays important cellular roles during mitosis. We therefore assessed whether DPF3 colocalizes with centrosomes throughout the stages of cell cycle. In the G1 and S phase, DPF3 staining was present around the centriole core in an irregular PCM1-like pattern (Fig. 2). Mitotic cells were identified based on nuclear condensation patterns representative of each mitotic stage as well as on the position of the centrosome using anti-γ-tubulin antibodies. DPF3 showed a centrosomal localization in prophase. In prometaphase and metaphase, DPF3 signals were detectable at the centrosome and remained associated with these structures throughout mitosis. At these stages, DPF3 also appeared to be located on microtubules. Remarkably, DPF3 staining clearly appeared in the spindle midzone in anaphase and was also found at the midbodies during late telophase and cytokinesis.

**Fig. 3.**
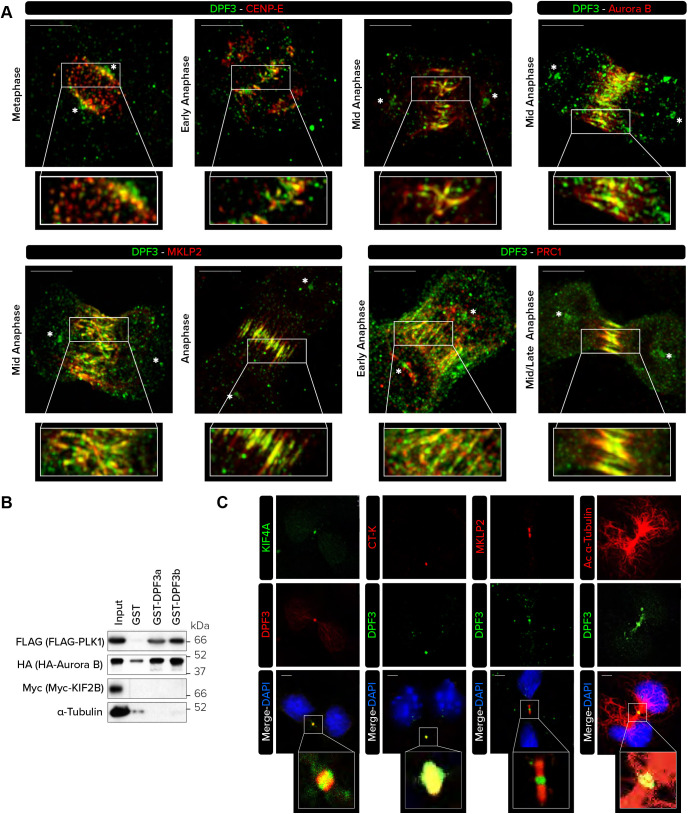
**DPF3 colocalizes with the chromosome passenger complex and bridging fibers during metaphase.** (A) HeLa cells were co-stained for DPF3 (in green) and CENP-E, Aurora B, MKLP2 (also known as KIF20A) or PRC1 (in red). Merged channels and magnifications of boxed regions are shown. Images were captured with Zeiss LSM 880 Airyscan high-resolution microscope. Asterisks (*) indicate centrosomes. Scale bars: 10 µm. (B) Extracts from U2OS cells mock-transfected or transfected with FLAG–PLK1, HA–Aurora B or Myc–KIF2B plasmids were incubated with glutathione-conjugated sepharose beads conjugated with GST alone, GST–DPF3a or GST–DPF3b. Input and pulled-down proteins were detected by western blotting using the indicated antibodies. (C) U2OS cells were co-stained for DPF3 (in green or red) and KIF4A (in green), citron kinase (CT-K), MKLP2 and acetylated α-tubulin (in red). Individual channels, merged channels with DAPI nuclear staining (in blue) and magnifications of boxed regions are shown. Scale bars: 5 µm. Immunofluorescence and western blotting images are representative of two independent experiments.

The spindle midzone plays a crucial role in regulating the completion of mitosis. To better characterize the localization of DPF3 in this multifunctional spindle midzone during anaphase, co-stainings were performed to more precisely detect CENP-E – a kinetochore-associated kinesin-like motor protein (Ciossani et al., 2018), Aurora B (AURKB) and MKLP2 (KIF20A) – two subunits of the chromosomal passenger complex (CPC) (Carmena et al., 2012; Serena et al., 2020), and PRC1 – a component of the bridging fibers, which are non-kinetochore microtubule structures that connect sister K-fibers and balance the tension on kinetochores (Conkar et al., 2019; Kajtez et al., 2016; Jagrić et al., 2021; Vukušić et al., 2017). In metaphase, a faint DPF3 signal was readily evident at the kinetochore, where it was slightly superimposed with that of CENP-E (Fig. 3A). At this stage, not all kinetochores that were occupied by CENP-E were found to contain DPF3, as the staining intensity of DPF3 varied among the kinetochores that displayed CENP-E staining. During early anaphase, DPF3 and CENP-E remained colocalized at kinetochores, although DPF3 localized to portions of the spindle midzone that did not overlap with CENP-E. By mid-anaphase, colocalization between DPF3 and CENP-E in the central spindle zone was more prominent. At this stage, we remarkably observed a perfect colocalization of DPF3 with Aurora B and MKLP2 as well as a partial overlap with the microtubule cross-linker PRC1. In late anaphase, when PRC1 is actively involved in midzone organization (Vukušić et al., 2021), a perfect colocalization was observed. These observations suggest that DPF3 might be part of the CPC, which overlaps bridging fiber structures during anaphase.

To identify potential interactions between DPF3 and CPC components such as Aurora B or PLK1 (Lee et al., 2021; Chu et al., 2011), GST pull-down experiments were performed. Incubation of protein extracts from U2OS cells expressing FLAG–PLK1 or HA–Aurora B constructs with GST alone or with GST–DPF3a or GST-DPF3b followed by western blotting analysis revealed positive interactions with both the DPF3a and DPF3b isoforms, whereas Myc–KIF2B and endogenous α-tubulin were not pulled down (Fig. 3B).

Moreover, because DPF3 was located in microtubule-enriched structures such as bridging fibers, we checked whether purified recombinant GST–DPF3a and/or GST–DPF3b directly interact with microtubules. Using a microtubule co-sedimentation assay, we found that a substantial amount of purified GST–DPF3a, and to a lesser extent GST–DPF3b, was pelleted with taxol-stabilized microtubules, whereas without microtubules, both DPF3a and DPF3b remained in the supernatant fraction (Fig. S3A,B). As shown in Fig. 3B, we found by the GST pull-down assay that neither DPF3a nor DPF3b was able to interact with α-tubulin, suggesting that DPF3a and DPF3b preferentially interact with microtubules over free α-tubulin monomers.

Finally, we also detected DPF3 in the central region of the cytoplasmic bridge – the midbody – during late telophase and cytokinesis, although a weak staining was still visible at the centrosomes (Fig. 3C). To more precisely identify the localization of DPF3 in the midbody compartments, co-immunofluorescence with antibodies against KIF4A (marker of midbody core), citron kinase (CT-K or CIT – marker of midbody ring) and MKLP2 (marker of midbody arms) was performed (Fig. 3C). DPF3 was mainly detected with both KIF4A and CT-K, suggesting that DPF3 is a component of the Flemming body, composed of the midbody ring and midbody core.

Taken together, these results showed that DPF3 is not only located in the centrosome throughout mitosis, but also appears in the spindle midzone and bridging fiber area during anaphase, and finally concentrates in the midbody during cytokinesis.

### DPF3 depletion induces G2/M blocking and apoptotic cell death

**Fig. 4.**
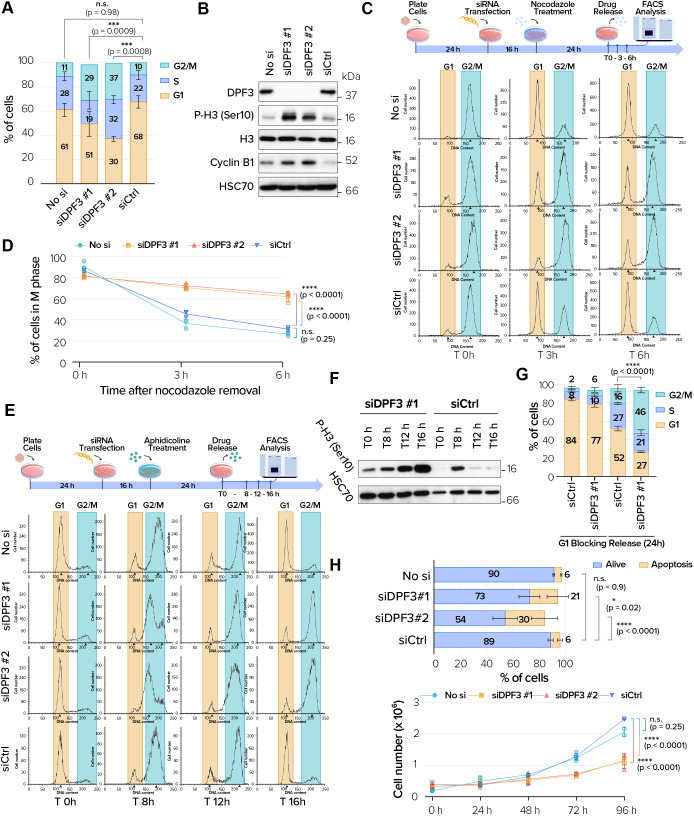
**DPF3 is required for faithful mitotic progression and genomic stability.** (A) Cell cycle analysis of U2OS cells mock-transfected (‘No si’) or transfected with one of two different DPF3 siRNAs (siDPF3 #1 and siDPF3 #2) or control siRNA (siCtrl). (B) Protein extracts from U2OS cells transfected as in A were analyzed by western blotting for the indicated proteins at the indicated time points. Blots are representative of three independent experiments. (C) U2OS cells were transfected as in A. After transfection, cells were incubated for 24 h with nocodazole (0.1 mg/ml). Cells were then released from the block by washing away the drug and changing to fresh complete medium. Samples were collected at the indicated times and processed for flow cytometry. FACS profiles are representative of one experiment performed in triplicate. (D) Quantification of FACS experiments as described in C. The graph shows the percentage of cells in G2/M at each of the indicated time points after nocodazole release. (E,F) U2OS cells were transfected as in A. After transfection, cells were incubated for 24 h with aphidicolin (1 μg/ml). Cells were then released from the block by washing away the drug and changing to fresh complete medium. Samples were collected at the indicated times and processed for flow cytometry. FACS profiles are representative of one experiment performed in triplicate (E). In parallel, protein extracts were prepared at the indicated times and analyzed by western blotting for the indicated proteins (F). Blots are representative of three independent experiments. (G) U2OS cells were transfected with control siRNA (siCtrl) or DPF3 siRNA (siDPF3 #1). After transfection, cells were incubated for 24 h with the CDK4 inhibitor CAS 546102-60-7 (5 µM). Cells were then released from the block by washing away the drug and changing to fresh complete medium. Samples were collected 24 h later and processed for flow cytometry. Quantification of FACS experiments shows the proportion of cells in each cell cycle stage 24 h after treatment with the CDK4 inhibitor and 24 h after the G1 release. (H) Quantification of apoptotic cell death in U2OS cells transfected as in A. (I) Growth curve analysis of HeLa cells transfected as in A. Graphs in A,D,G–I show mean±s.d. from three independent experiments. *P*-values were calculated using one-way ANOVA with Tukey's post hoc test (A,D,G,I) or two-way ANOVA with Dunnett's post hoc test (H). n.s., not significant; *P<0.05; ***P<0.001, ****P<0.0001.

DPF3 was located in key mitotic structures including the centrosome, the spindle midzone and bridging fiber area, and midbodies. Because such structures are important for orchestrating mitotic cell division, we assessed the impact of DPF3 knockdown on the cell cycle. Transfection with the two different siRNAs against DPF3a and DPF3b in asynchronous U2OS cells caused a dramatic increase in the G2/M cell population compared with that in cells transfected with an irrelevant Gl3 siRNA (siCtrl) (Fig. 4A). Interestingly, specific siRNA depletion of Brm and Brg1 did not block cells in G2/M (Fig. S4A,B), in agreement with a previous publication (Giles et al., 2021). Increased histone H3 phosphorylation on serine 10, a late G2/M marker, was also observed in total protein extracts of DPF3-depleted cells (Fig. 4B), supporting that the cells accumulated in the G2/M phase. In addition, higher expression of cyclin B1 was observed in protein extracts from DPF3-depleted cells (Fig. 4B), indicating an unsatisfied spindle assembly checkpoint. We tested the possibility that the G2/M blocking of DPF3-depleted cells could be rescued by overexpressing exogenous DPF3. Unfortunately, overexpression of the specific DPF3a and DPF3b isoforms in U2OS cell line triggered the formation and accumulation of protein like-aggregates (Fig. S4C). Because maintenance of protein solubility is a fundamental aspect of cellular homeostasis, DPF3 aggregation could not lead us to determine the effects of DPF3 re-expression.

To confirm that DPF3-depleted cells were arrested in M phase, we blocked control or DPF3-depleted U2OS cells at early mitosis using nocodazole and then released them into fresh medium. We found that 60% of DPF3-depleted cells were still in the mitotic phase 3 h after release, whereas more than 50% of control cells had already exited mitosis (Fig. 4C,D). To firmly validate that cell cycle blocking occurred in the M phase, we analyzed cell cycle progression after release from an early S phase block by aphidicolin treatment (often termed G1/S synchrony). Fluorescence-activated cell sorting (FACS) analysis showed that a large population of both control and DPF3-depleted cells reached G2/M phase 8 h after release from the aphidicolin block. Control cells could then progress further in the cell cycle and accumulated in the next G1 phase 16 h after the release from the aphidicolin block. In contrast, DPF3-depleted cells were still detected in the G2/M phase 16 h after release from early S phase (Fig. 4E). In parallel with FACS analysis, proteins were also extracted from DPF3-depleted cells at the indicated time intervals and were afterwards analyzed by western blotting using an antibody against phosphorylated serine 10 on histone H3. An increase of histone H3 phosphorylation on serine 10 was observed 8 h after release from the aphidicolin block. The levels decreased 12 and 16 h after release (Fig. 4F), when control cells started to leave the G2 phase and mitosis and accumulated in the next G1 phase. In contrast, the levels remained high in DPF3-depleted cells during the same time intervals, indicating that these cells were still blocked in the G2/M phase.

Because aphidicolin can induce DNA damage and activates a G2 checkpoint response (Arlt et al., 2004; Sánchez-Molina et al., 2011), it is possible that DPF3-depleted cells could not progress to mitosis due to G2 blocking. We performed another cell cycle analysis using CAS 546102-60-7, a validated inhibitor of CDK4/cyclin D1, which induces G1 cell cycle arrest (Zhu et al., 2003). FACS analysis showed that control cells could normally progress in the cell cycle 24 h after the G1 block induced by the CDK4 inhibitor and accumulated in the next phase (Fig. 4G). However, a significant number of DPF3-depleted cells were still detected in the G2/M phase 24 h after the G1 release, as similarly observed with aphidicolin.

As prolonged mitotic arrest can eventually result in cell death, we also assessed the induction of apoptosis upon DPF3 depletion. An annexin V/propidium iodide (PI) staining experiment clearly showed a higher proportion of apoptotic cells upon DPF3 depletion compared to that in control cells (Fig. 4H). Cell cycle blockage and apoptosis induction upon DPF3 depletion translated into a significant decrease in cell number *in vitro* (Fig. 4I). Finally, live-cell imaging observations (Movies 1 and 2) were in full agreement with these data and clearly demonstrated that DPF3 depletion resulted in mitotic blocking, followed by cell death.

### DPF3 depletion induces chromosome oscillation, chromosome misalignment and prolonged mitosis

**Fig. 5.**
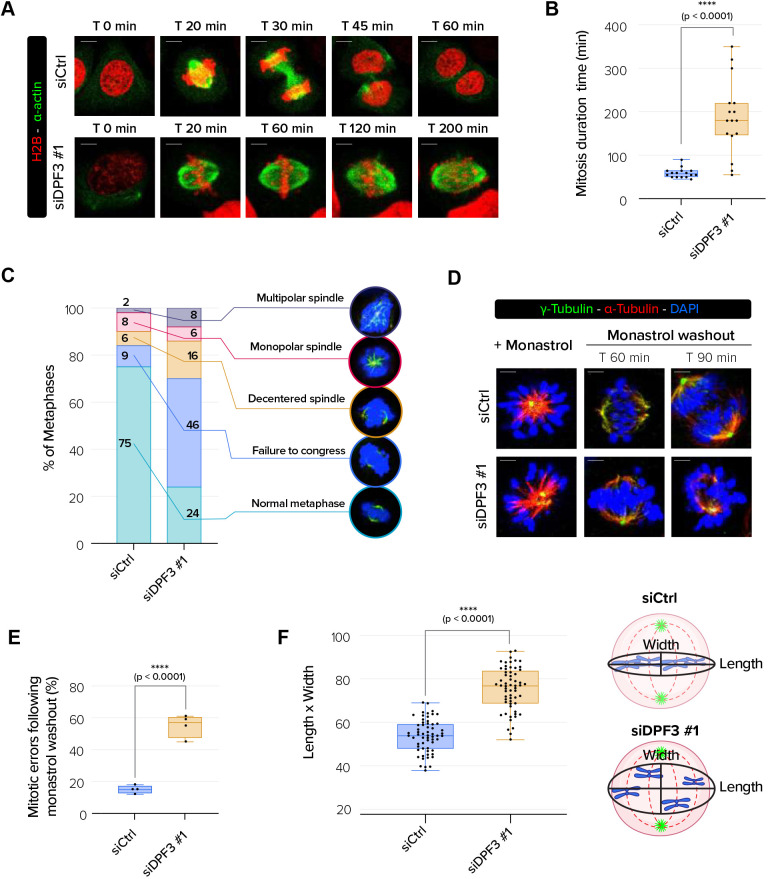
**DPF3 is required for chromosome alignment.** (A) Live-imaging experiments of H2B–RFP- and α-actin–GFP-expressing U2OS cells transfected with control siRNA (siCtrl) or DPF3 siRNA (siDPF3 #1) beginning 48 h post transfection. Scale bars: 5 µm. (B) Mitotic duration for H2AB–RFP- and α-actin–GFP-expressing U2OS cells transfected as in A (*n*=16 cells/condition from three independent experiments). (C) Quantification of the percentage of aberrant mitotic phenotype in U2OS cells transfected as in A (*n*=100 cells/conditions for one experiment). Representative images of abnormal mitotic phenotypes that were quantified are shown. (D) U2OS cells were transfected with control siRNA (siCtrl) or DPF3 siRNA (siDPF3 #1) and treated with monastrol (100 µM) for 3 h and then released in fresh culture medium. Cells were co-stained prior to monastrol washout and at 60 min or 90 min after washout. Mitotic spindles were labeled for α-tubulin (in red), γ-tubulin (in green) to mark centrosomes, and DAPI (in blue) to mark chromosomes. Merged channels with DAPI nuclear staining (in blue) are shown. Scale bars: 5 µm. (E) Quantification of the mitotic errors following monastrol washout from the experiment described in D (*n*=a total of 70 cells for each condition from three independent experiments). (F) Quantification of chromosome misalignment. The length/width ratio was determined as schematized in the right panel and calculated for each condition (*n*=20 cells/condition/experiment from three independent experiments). Boxes in B,E,F show the 25–75th percentiles, the line shows the median, and whiskers show the minimum and maximum values. *P*-values were calculated using unpaired two-tailed *t*-test (B,E,F). ****P<0.0001.

To further understand the cause of the mitotic blockage observed in DPF3-depleted cells, we performed fluorescence live-cell imaging experiments using U2OS cells expressing H2B–RFP and α-actin–GFP. Control cells showed proper mitotic division (Movie 3). In contrast, we observed that DPF3-depleted cells exhibited chromosome hyper-oscillations and spent an unusually long time in mitosis (Movies 4 and 5, Fig. 5A). Indeed, live-cell imaging revealed that DPF3-depleted cells were delayed in mitosis (180 min spent in mitosis for DPF3 siRNA#1-treated cells, compared to 60 min for Ctrl siRNA-treated cells) (Fig. 5B). After a delay at metaphase, a proportion of DPF3-depleted cells did undergo anaphase and subsequent cytokinesis, leading to asynchronous or unscheduled chromatid separation as well as formation of nuclei with irregular shapes (formation of bi- or multi-lobed nuclei) (Movies 6 and 7). Furthermore, we frequently observed induction of post-mitotic apoptotic cell death upon DPF3 depletion (Movies 2 and 8), which is indicative of genomic instability.

To better assess the type of mitotic profile defects upon DPF3 depletion, we fixed cells and determined the percentage of cells with aberrant mitotic profiles, including misaligned chromosomes, unaligned spindles and monopolar or multipolar spindles (Fig. 5C). Most control cells (>75%) showed normal metaphases. Consistent with the hyper-oscillating chromosomes, DPF3 depletion induced a significant proportion of cells (around 50%) harboring chromosome misalignment.

To further validate the impact of DPF3 depletion on chromosome alignment, we treated cells with the Eg5 inhibitor monastrol and then monitored the restoration of KT–MT attachments during spindle bipolarization in response to drug washout. When released in medium containing MG132, a proteasome inhibitor that causes metaphase arrest, control cells established bipolar spindles and most chromosomes aligned correctly, with less than 10% of these cells with subsequent mitotic defects (Fig. 5D,E). In contrast, numerous chromosomes remained unaligned and syntelic KT–MT attachment was clearly observed in more than 50% of DPF3-depleted cells. To further analyze the unaligned position of chromosomes at the metaphase plate, we took images of metaphase plates that were horizontally oriented with respect to the imaging plane (maximum-intensity projections of *z*-stack scans). We measured the distance of the DAPI-stained chromosomes that were furthest from the metaphase plate (length) and the spindle axis (width) and then determined the ratio (Fig. 5F). This quantification revealed that the ratio was significantly higher (by 30%) in DPF3-depleted cells than in control cells, suggesting that chromosomes were more dispersed around the metaphase plate upon DPF3 depletion. Altogether, these results demonstrate that DPF3 depletion induced chromosome misalignment.

### DPF3 depletion alters K-fiber stability and the establishment of stable KT–MT attachments

**Fig. 6.**
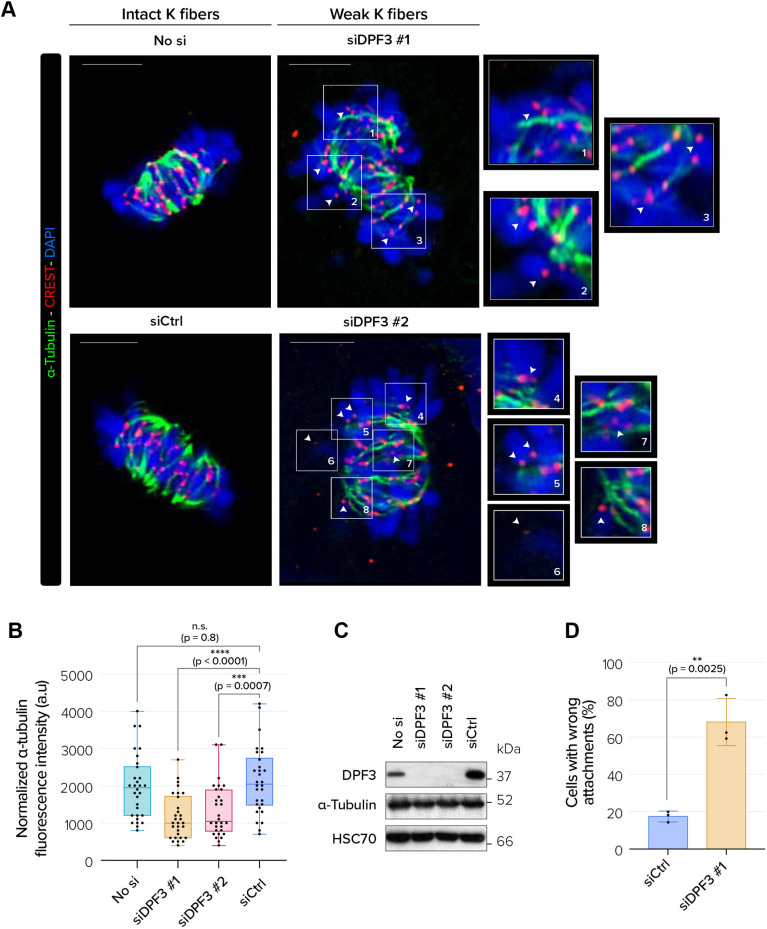
**DPF3 is required for stabilizing K-fibers and robust KT–MT attachments in metaphase.** (A) U2OS cells were mock-transfected (‘No si’) or transfected with one of two different DPF3 siRNAs (siDPF3 #1 and siDPF3 #2) or control siRNA (siCtrl), and then subjected to cold-stable microtubule assay after mitotic arrest in metaphase. Mitotic spindles were labeled for α-tubulin (in green), kinetochores with CREST (in red) and chromosomes with DAPI (in blue). Merged images of maximum-intensity projections of *z*-stack planes (0.25 µm intervals) are shown. White arrowheads and magnifications of boxed areas show less stable and robust kinetochore–microtubule (KT–MT) attachment. Scale bars: 5 µm. (B) Quantification of α-tubulin fluorescence intensity in U2OS cells mock-transfected (‘No si’) or transfected with one of two different DPF3 siRNAs (siDPF3 #1 and siDPF3 #2) or control siRNA (siCtrl). (*n*=10 cells/condition/experiment from three independent experiments). Boxes show the 25–75th percentiles, the line shows the median, and whiskers show the minimum and maximum values. a.u., arbitrary units. (C) Protein extracts from U2OS cells mock-transfected (‘No si’) or transfected with one of two different DPF3 siRNAs (siDPF3 #1 and siDPF3 #2) or control siRNA (siCtrl) were analyzed by western blotting for the indicated proteins. Blots are representative of three independent experiments. (D) Quantification of the percentage of defective KT–MT attachment in U2OS cells transfected as in A. The graph shows mean±s.d. from three independent experiments (*n*≥20 cells/condition/experiment). *P*-values were calculated using one-way ANOVA with Tukey's post hoc test (B) or unpaired two-tailed *t*-test (D). n.s., not significant; **P<0.01; ***P<0.001, ****P<0.0001.

Chromosome hyper-oscillations and failure in chromosome congression upon DPF3 depletion could arise from the destabilization of microtubules and/or loss of KT–MT attachments. To evaluate the effect of DPF3 depletion on stable end-on KT–MT attachments, we performed a functional assay termed the cold-stability assay. Unstable or weakened KT–MT attachments are disassembled when subjected to a low temperature for a short period, whereas stable and robust attachments are resistant to this condition. Control and DPF3-depleted U2OS cells were stained with anti-α-tubulin (marker for K-fibers) and CREST (a marker of kinetochores) antibodies. In control cells, K-fibers were stable because they were well defined and bright (Fig. 6A). On the contrary, K-fibers in DPF3-depleted cells were wavy and discontinuous, and exhibited a low density of α-tubulin staining, indicating a loss of K-fiber stability. To further quantify unstable K-fibers, we measured α-tubulin fluorescence (maximum-intensity projections of *z*-stack scans) (Zhou et al., 2019; Warren et al., 2020; Batman et al., 2022). DPF3 depletion resulted in a 40% decrease in α-tubulin fluorescence intensity compared to that in control cells (Fig. 6B). Western blot analyses of lysates prepared from control and DPF3-depleted cells showed that the intensity changes in spindle microtubules were not due to altered cellular abundance of α-tubulin (Fig. 6C). In DPF3-depleted cells, we frequently observed fewer cold-resistant K-fibers and several less stable and robust KT–MT attachments, inducing mal-oriented chromosomes (Fig. 6A, white arrowheads; Fig. 6D). Collectively, these results demonstrate that DPF3 depletion alters K-fiber stability and weakens KT–MT attachments, thereby compromising chromosome movement and alignment, which results in the aforementioned mitotic defect phenotype.

### DPF3 is a centriolar satellite protein involved in primary ciliogenesis and localizes at the apical surface in multiciliated cells

**Fig. 7.**
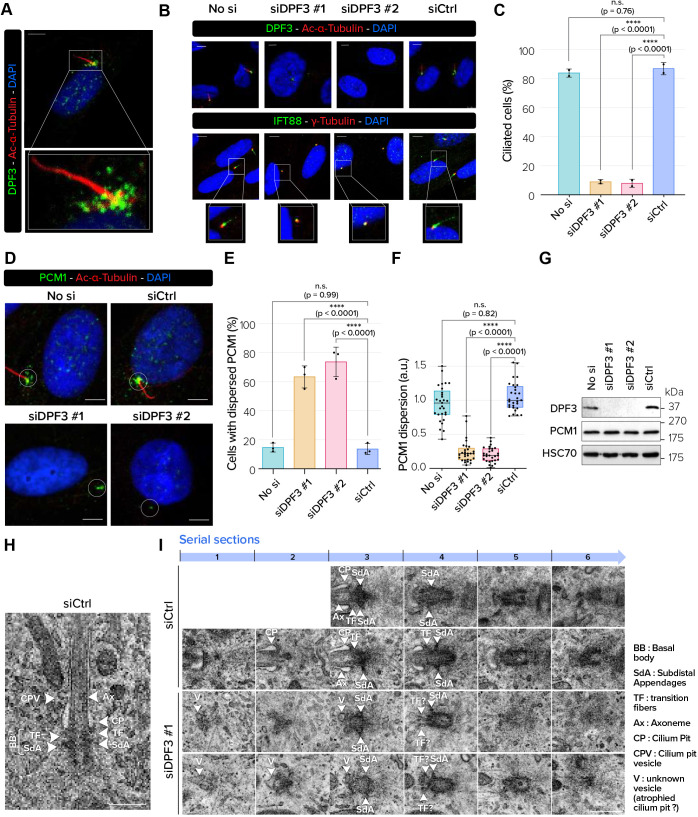
**DPF3 depletion disrupts centriolar satellite stability and primary cilium formation by blocking axoneme extension.** (A) Serum-starved ciliated hTERT-RPE-1 cells were co-stained for DPF3 (in green) and acetylated α-tubulin (in red). Merged channels with DAPI nuclear staining (in blue) and magnifications of boxed regions are shown. (B) hTERT-RPE-1 cells were mock-transfected (‘No si’) or transfected with one of two different DPF3 siRNAs (siDPF3 #1 and siDPF3 #2) or control siRNA (siCtrl), serum starved for 48 h, and co-stained for DPF3 or IFT88 (in green) and acetylated α-tubulin or γ-tubulin (in red). Merged channels with DAPI nuclear staining (in blue) are shown. (C) Quantification of the percentage of ciliated hTERT-RPE-1 cells after siRNA transfection as described in B. The graph shows the mean±s.d. from three independent experiments (*n*=50 cells/condition/experiment). (D) hTERT-RPE-1 cells were treated as in B and co-stained for PCM1 (in green) and acetylated α-tubulin (in red). Merged channels with DAPI nuclear staining (in blue) are shown. White circles indicate widely dispersed PCM1 granules in control conditions (top) and altered patterns upon siRNA treatment (bottom). (E) Quantification of hTERT-RPE-1 cells without dispersed PCM1 profiles after siRNAs transfection. The graph shows mean±s.d. from three independent experiments (*n*=100 cells/condition/experiment). (F) The pericentrosomal fluorescence intensity of PCM1 was measured in a circular area drawn around the centrosome, and the signal level was normalized to 1 in the ‘No si’ condition (*n*=10 cells/condition/experiment from three independent experiments). Boxes show the 25–75th percentiles, the line shows the median, and whiskers show the minimum and maximum values. a.u., arbitrary units. (G) Protein extracts from hTERT-RPE-1 cells transfected as described in B were analyzed by western blotting for the indicated proteins. Blots are representative of two independent experiments. (H,I) Transmission electron micrographs of mother centrioles and the axoneme (serial sections) in hTERT-RPE-1 cells transfected with control siRNA (siCtrl) or DPF3 siRNA (siDPF3 #1). White arrowheads indicate different ciliary structures described in the panel legend. Images are representative of two independent experiments. Ax, axoneme; BB, basal body; CP, cilium pit; CPV, cilium pit vesicle; SdA, subdistal appendage; TF, transition fibers; V, vesicle. Scale bars: 5 µm (A,B,D); 500 nm (H,I). *P*-values were calculated using one-way ANOVA with Tukey's post hoc test (C,E,F). n.s., not significant; ****P<0.0001.

Many proteins associated with centriolar satellites are involved in the formation and function of the primary cilium. To investigate whether DPF3 was also located in centriolar satellites during ciliogenesis, we performed DPF3 staining in hTERT-RPE-1 cells grown in serum-deprived conditions to assemble primary cilia. In ciliated hTERT-RPE-1 cells visualized with anti-acetylated α-tubulin, DPF3 staining was detected in granular structures and showed a similar staining profile to that of PCM1, suggesting that DPF3 localizes to centriolar satellites (Fig. 7A,D). To determine whether DPF3 depletion resulted in a defect in primary cilium formation, we transfected hTERT-RPE-1 cells with DPF3 siRNAs and cultured them in serum-free conditions to induce cilium formation. FACS analysis was first performed to validate that both control and DPF3-depleted cells were arrested in the G0/G1 phase of the cell cycle when cilium formation occurs (Fig. S5). Under these conditions, we found that a large majority of DPF3-depleted cells (91% and 82% for siDPF3#1 and siDPF3#2, respectively) showed a lack of acetylated α-tubulin signal in the axoneme (Fig. 7B, top panel; Fig. 7C). IFT88 staining along the ciliary axoneme was also dramatically decreased upon DPF3 depletion, whereas it was still detected at the mother centriole or basal body compartment (Fig. 7B, bottom panel). Taken together, these data provide evidence that the axoneme extension is impaired upon DPF3 depletion. Interestingly, the dispersion and distribution of PCM1 granules in DPF3-depleted hTERT-RPE-1 cells was changed (Fig. 7D–F). Centriolar satellite dispersion was not caused by a change in PCM1 protein expression level (Fig. 7G), indicating that centriolar satellites remain intact upon DPF3 depletion. Taken together, these data suggest that the inability of DPF3-depleted cells to ciliate is probably related to defective centriolar satellite organization or distribution around the centrosome rather than assembly per se. To better understand how DPF3 depletion impaired ciliogenesis, we examined centrioles and ciliary structures of serum-starved hTERT-RPE-1 cells depleted for DPF3 by transmission electron microscopy (TEM). Ciliogenesis is a multistep process that begins with the docking of small Golgi-derived ciliary vesicles to the distal appendages of mother centrioles leading to the formation of distal appendage vesicles (DAVs). Small DAVs fuse together and form larger primary ciliary vesicles, followed by the formation and extension of the ciliary axoneme (Zhao et al., 2023). Control cells displayed mother centrioles associated with the ciliary pockets with extended axonemal shafts (Fig. 7H,I). In DPF3-depleted cells (which represents around 90% of cells; see Fig. 7B, siDPF3 #1), the elongated ciliary axoneme was clearly absent. Interestingly, the docking of small ciliary vesicles to mother centrioles appeared to be affected upon DPF3 depletion. Docking of the mother centriole to the pre-ciliary vesicles and the initiation of axoneme extension requires centriolar satellites (Hall et al., 2023). We can therefore hypothesize that DPF3-depleted cells displayed reduced docking of the mother centriole to the ciliary vesicle and subsequent axoneme extension, probably due to destabilization or reorganization of centriolar satellites.

## DISCUSSION

Per our previous understanding, DPF3 was recognized solely as a subunit of the BAF complex, existing as two isoforms. The established function of the DPF3b isoform is binding to acetylated and/or methylated histones, thereby allowing the recruitment of the BAF complex to specific genomic regions during development (Lange et al., 2008; Zend et al. 2010). As for DPF3b, the DPF3a isoform can bind the BAF complex but mainly through bridge proteins such as HRP2 (Zhu et al., 2020). So far, both isoforms have therefore been considered as regulators of gene expression.

**Fig. 8.**
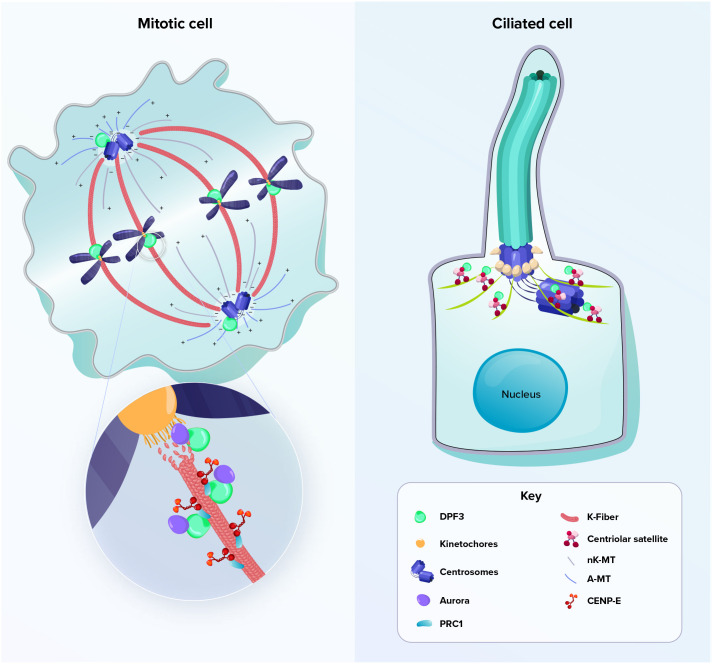
**Schematic representation of DPF3 localization during mitosis and ciliogenesis.** Left: DPF3 is located in the centrosomes and spindle midzone in mitotic cells. During the metaphase to anaphase transition, DPF3 is present at a microtubule-binding interface and might connect the chromosome passenger complex to the chromosomes or kinetochore structure. Right: in ciliated cells, DPF3 localizes to centriolar satellites. A-MT, astral microtubule; K-fiber, kinetochore fiber; nK-MT, non-kinetochore microtubule.

Here, we provide new evidence that DPF3 accumulates at different mitotic structures during mitosis, including the centrosomal compartment, spindle midzone and bridging fiber area, and midbodies. DPF3 depletion triggered aberrant outcomes of mitosis and apoptosis, thus causing genomic instability (Fig. 8, left). This unexpected localization of DPF3 in mitotic structures classifies DPF3 as a new moonlighting protein playing an important role in mitosis and completes the repertoire of chromatin-associated proteins, such as the ISWI, CHD4, INO80 ATPase, NuSAP or subunits of SRCAP and the p400–TIP60 chromatin remodeling complexes, that moonlight as mitotic regulators (Yokoyama et al., 2009, 2013; Raemaekers et al., 2003; Hur et al., 2010; Chambers et al., 2012; Messina et al., 2022). Different considerations support the hypothesis that the DPF3-dependent mitotic phenotype is due to its direct (moonlighting) role rather than to an indirect effect on transcription of genes encoding mitotic proteins. Firstly, mitotic alterations such as K-fiber instability, chromosome misalignment or multi-lobed cells are consistent with the localization of DPF3 to the mitotic apparatus and are frequently observed after the knockdown of multiple mitotic regulators (Zhou et al., 2019; Wei et al., 2011; Bendre et al., 2016). Secondly, the absence of interaction of DPF3 with the core ATPase BAF subunits Brg1 and Brm as well as the lack of G2/M blocking upon Brg1 or Brm depletion suggest that DPF3 exerts its mitotic function in a Brg1/Brm-independent manner. Moreover, the dissociation of Brm and Brg1 from mitotic chromatin without any direct function in spindle assembly or maintenance as evidenced in the literature (Muchardt et al., 1996) strengthens our hypothesis that DPF3 acts in a BAF-independent manner during mitosis.

From a mechanistic point of view, we observed that DPF3 is involved in K-fiber stability and correct chromosome positioning during metaphase. The metaphase plate appeared to form correctly upon DPF3 depletion. However, we observed alignments with hyper-oscillating chromosomes. Consistent with the hyper-oscillating chromosomes, DPF3 depletion induced an increased fraction of cells harboring chromosome misalignment. One consequence of hyper-oscillating chromosomes in DPF3-depleted cells is that they have a propensity to lose their attachment to the mitotic spindle and activate the spindle assembly checkpoint, thus explaining the mitotic delays. Similar observations have been reported in the context of SF3B1 mutation (Cusan et al., 2023) or upon KIF18A depletion (Janssen et al., 2018), a mitotic kinesin which is also found within the bridging fiber (Jagrić et al., 2021).

During mitosis, the CPC – a multiprotein complex composed of an enzymatic component (Aurora B) along with numerous regulatory proteins – dynamically localizes to different subcellular locations. It is located to the inner centromere region during metaphase and it moves to the central spindle microtubules during anaphase. Interaction and colocalization data support evidence that DPF3 might be a regulator of CPC localization and activity during the metaphase to anaphase transition. Interactions between the CPC and both the chromatin and microtubules ensure that chromosomes are properly connected to microtubules from opposite poles of the mitotic spindle. The dual chromatin–microtubule interactions of the CPC might, at least in part, depend on structural features of DPF3. Inner centromeric chromatin is enriched in epigenetic marks such as the dimethylation of lysine 4 on histone H3 (H3K4me2) (Sullivan and Karpen, 2004). Interestingly, this histone modification pattern can be read and bound by the double PHD finger of DPF3b (Lange et al., 2008), suggesting that DPF3b could act as a ‘reader’ of the centromere epigenetic code to recruit the CPC. The temporal regulation of CPC localization to the inner centromere is controlled by TIP60-dependent acetylation of lysine 4 on histone H3 (H3K4Ac) (Niedzialkowska et al., 2022), an epigenetic mark also recognized by DPF3b (Lange et al., 2008). Deciphering the repertoire of histone post-translational modifications that influence the recruitment of DPF3 to chromosomes to regulate the formation of the CPC deserves detailed investigations. In addition to its histone-reader function, we hypothesize that DPF3 might recruit or correctly position the CPC to the microtubule lattice via its microtubule-binding capacity. Indeed, we provide in this study new experimental evidence that DPF3a, and to a lesser extent DPF3b, has the ability to recognize polymerized microtubules.

Kinetochores and the CPC are considered as biomolecular condensates that are formed by liquid–liquid phase separation (LLPS) (Liu et al., 2020; Trivedi and Stukenberg, 2020; Niedzialkowska et al., 2024). LLPS is often mediated by IDPs. These IDPs are characterized by a lack of stable secondary and tertiary structures and possess high conformational flexibility. Due to their flexible structure, IDPs are able to interact specifically but transiently or weakly with multiple protein partners, hence acting as ‘hubs’ in protein–protein interaction networks that drive the formation of biocondensates. Using *in silico* predictions and experimental evidence, we have recently demonstrated that both DPF3a and DPF3b isoforms contain IDRs (Mignon et al., 2021, 2022). Through their IDRs, DPF3a and DPF3b would be able to dynamically interact with multiple protein partners, driving the formation of a large protein structure connecting the centromere or kinetochore and the CPC to the microtubule lattice.

The multiple and dynamic protein–protein interactions implicated in the assembly of huge protein complexes can be made possible and finely regulated by various post-translational modifications on IDPs such as phosphorylation. Schick et al. (2019) reported an uncharacterized interaction between DPF3 and PLK1, a crucial kinase being required for diverse mitotic functions (Combes et al., 2017). Remarkably, we also found that DPF3 and PLK1 are present in the same complex. Several studies demonstrated that accumulation of proteins such as MyoGEF (also known as PLEKHG6) (Asiedu et al., 2008), CLASP2 (Maia et al., 2012) or CEP55 (Fabbro et al., 2005) at the mitotic apparatus relies on phosphorylation by PLK1. As a model, we can therefore propose that PLK1-dependent phosphorylation of DPF3 might be important not only for its correct localization to mitotic structures, but also for its interaction with different mitotic partners. Conversely, it has also been described that the correct localization of PLK1 in mitotic structures as well as its kinase activity are tightly regulated. Various mitotic proteins and chromatin remodelers such as CLIP-170 (or CLIP1), NCAPG2, NudC or RSF1 (Amin et al., 2014; Kim et al., 2014; Nishino et al., 2006; Lee et al., 2021) are involved in the tethering of active PLK1 to mitotic structures. At centromeres, PLK1 phosphorylates borealin (CDCA8) and survivin (BIRC5) in the CPC, and this step is important for Aurora B docking (Lee et al., 2021) and activities such as the phosphorylation of BubR1 or CENP-E (Ditchfield et al., 2003; Kim et al., 2010). In this respect, we could also propose an alternative model according to which DPF3 would act as a recruiter and spatial regulator of PLK1, thus playing the role of a bridge protein for the interaction between PLK1 and the CPC. Regardless of the molecular mechanism, a better characterization of the DPF3–PLK1–Aurora B interaction could provide important molecular insights into the regulation of mitosis.

One key observation in our study was the localization of DPF3 in centriolar satellites in interphase. Despite disappearance of centriolar satellites during mitosis (Kubo et al., 1999), DPF3 remained at the centrosomal position, as similarly observed for other centriolar satellite proteins such as OFD1 or CEP290 (Lopes et al., 2011). The observation that DPF3 is still present at the centrosomes during mitosis suggests an as-yet-unidentified role in this compartment. K-fiber plus-end dynamics are set by kinetochores themselves, although they can be also influenced by centrosomes (Dudka et al., 2019). The localization of DPF3 at the two attachments sites during mitosis – the centrosomes and the spindle midzone – suggests that DPF3 exerts a tethering function between microtubule ends and either centrosomal or kinetochore proteins. A similar role and function have been attributed to the CEP57 protein, which is present at the attachment interfaces on both ends of mitotic microtubules (Emanuele and Stukenberg, 2007).

Because centriolar satellites are required for efficient ciliogenesis, it is not surprising that DPF3 also plays a crucial role in primary cilium formation, in addition to its function in mitosis. In a model of primary ciliogenesis, we observed that DPF3 localized to centriolar satellites that cluster at the base of the centrosome–cilium complex. More importantly, we demonstrated that DPF3 knockdown disrupted the organization or distribution of centriolar satellites and blocked axoneme extension *in vitro* (Fig. 8, right). Similar observations have been reported for centrosome-related proteins such as LRRC45 or MARK4, which participate in ciliogenesis through axoneme extension (Kurtulmus et al., 2018; Kuhns et al., 2013).

Centriolar satellites as well as centrosomes or the pericentriolar matrix are also membrane-less organelles that are formed by the LLPS phenomenon (Zhao et al., 2021; Woodruff et al., 2017; Jiang et al., 2021; Raff, 2019; Liu et al., 2020). IDPs are also crucial components of these organelles and determine their respective activities. Dispersion of centriolar satellites upon DPF3 depletion therefore indicates that DPF3 might be a key IDP driving the homeostasis of these membrane-less granules by interacting with multiple partners. Interactome profiling will certainly help to identify centriolar satellite partners. Although the precise molecular function of DPF3 in cilia should be further addressed, our study opens a new avenue of interpretation for previously published functions of DPF3. Lange et al. (2008) suggested that abnormal cardiac looping mediated by DPF3 knockdown was caused by BAF-dependent transcriptional deregulation of genes encoding heart proteins. Because disturbed heart looping is frequently associated with primary cilium dysfunction (Klena et al., 2017; Gabriel et al., 2021), it is possible that centriolar satellite-associated localization of DPF3 is crucial for assembly and function of cilia in developmental heart tissues. Moreover, it is becoming more and more evident that mutations in centriolar satellite proteins such as CEP290 or OFD1 can cause defects in ciliogenesis (Papon et al., 2010; Hannah et al., 2019). Whether deregulation of DPF3 is associated with ciliopathies deserves to be further explored. Indeed, a single nucleotide polymorphism (SNP) and overexpression or downregulation of DPF3 have been already reported in ciliopathy-related diseases such as tetralogy of Fallot (Kaynak et al., 2003; Lange et al., 2008), craniofacial ciliopathies (Vieira et al., 2008; Brugmann et al., 2010), Hirschsprung's disease (Liu et al., 2014; de Pontual et al., 2007) or glioblastoma (Hiramatsu et al., 2017; Yang et al., 2013; Hoang-Minh et al., 2018; Sarkisian and Semple-Rowland, 2019; Goranci-Buzhala et al., 2021). Interestingly, studies demonstrated that centriolar satellite proteins, such CEP131 or CCDC113, are required for sperm motility by regulating protein trafficking in sperm cells (Hall et al., 2013; Firat-Karalar et al., 2014). Mutation in the centriolar satellite protein CEP135 leads to the formation of aggregates in flagella and induces multiple morphological abnormalities of the sperm flagella, a rare disease associated with primary infertility (Sha et al., 2017). Interestingly, several clinical studies revealed that a SNP in DPF3 was highly correlated with low sperm count, accounting for motility defects and male infertility (Kosova et al., 2014; Liu et al., 2017; Sato et al., 2018). In addition, we have recently demonstrated that both DPF3a and DPF3b isoforms tend to spontaneously aggregate *in vitro* (Mignon et al., 2021, 2022; Leyder et al., 2022). Whether mutations in DPF3 would accelerate its aggregation, potentially affect the normal function of spermatozoids and consequently lead to male infertility should be further investigated.

In conclusion, our study reveals a moonlighting localization and function of the DPF3 protein during mitosis and ciliogenesis, supporting a previously unknown role in human development and diseases such as cancers and ciliopathies.

## MATERIALS AND METHODS

### Cell culture, synchronization and treatment

U2OS cells were maintained in McCoy's 5A medium (BE12-688F, Westburg). HeLa and MDA-MB-231 cancer cell lines and hTERT-RPE-1 cells were maintained in Dulbecco's modified Eagle's medium (DMEM; BWSTL0104-500, VWR). All media were supplemented with 10% heat-inactivated fetal bovine serum (FBS; A5256701, Gibco), and cells were incubated at 37°C in the presence of 5% CO_2_. T47D cells were cultured in RPMI 1640 supplemented with 10% FBS, and MCF7 cells were cultured in Minimum Essential Medium (MEM) supplemented with 10% FBS (10370021, Thermo Fisher Scientific). KE37 cells were cultured in Roswell Park Memorial Institute medium (RPMI 1640, BWSTL0500-500, VWR) supplemented with 7% FBS. All cell lines were from American Type Culture Collection. Each cell line was routinely examined by microscopy to check shape, growth conditions, proliferation and the presence of any contaminants. All cells were routinely (every 2 weeks) examined for mycoplasma contamination using the MycoAlertTM Mycoplasma Detection Kit (Lonza, LT07-118). Aphidicolin (A0781), nocodazole (M1404), RO-3306 (SML0569) and monastrol (M8515) drugs were from Sigma–Aldrich. MG132 was from MedChemExpress (HY-13259). The CDK4/cyclin D1 inhibitor CAS 546102-60-7 was from Calbiochem (219476).

### siRNA transfection

siRNAs were synthesized by Eurogentec and were used at a final concentration of 40 nM. Cells were plated 24 h before transfection and then transfected at a confluence of approximately 50% using the calcium phosphate method. Mock-transfected cells (‘No si’) and cells transfected with an irrelevant siRNA targeting Gl3 luciferase (siCtrl) were used as negative controls. Two different siRNAs targeting DPF3 were used unless indicated otherwise. The sequences of the siRNAs used in this study are listed in Table S1.

### Plasmids and plasmid DNA transfection

Transient plasmid DNA transfection was carried out using Lipofectamine 2000 transfection reagent (Thermo Fisher Scientific, 11668030) according to the manufacturer's instructions. Plasmids encoding DPF3a and DPF3b cDNA in fusion with the GST epitope were generated by e-Zyvec. pCMV3-KIF2B-Myc and pCMV3-hAURKB-HA were from Sino Biological. pTXB3-Flag PLK1 was from Addgene (plasmid #68271, RRID: Addgene 68271).

### Real-time quantitative PCR

Total RNA was extracted using the NucleoSpin RNA kit (Macherey–Nagel, 740955) and measured using a NanoDrop spectrophotometer (NanoDrop 1000, Thermo Fisher Scientific). Reverse transcription was performed with the RevertAid H minus first strand cDNA synthesis kit (Thermo Fisher Scientific, K163) according to the manufacturer's instructions. Quantitative PCR was performed with a LightCycler 480 instrument (Roche, 04707516001) with β-actin as the control. All measurements were realized in triplicate. Primer sequences are available in Table S2.

### Total protein extraction

For total protein extraction, adherent and floating cells were collected and lysed with a lysis buffer containing 1% SDS, 40 mM Tris-HCl (pH 7.5), 1 mM EDTA, complete EDTA-free protease inhibitor (Sigma-Aldrich, 11697498001) and phosphatase inhibitor (Sigma-Aldrich, 0490684500).

### Isolation of cytoplasmic, nucleoplasmic and chromatin-enriched fractions

Chromatin fractionation was carried out as previously described (Méndez and Stillman, 2000). Three different fractions were collected: the cytoplasmic fraction, the nuclear soluble fraction and the chromatin-enriched fraction. MEK2 and lamin A/C proteins were used as fractionation controls for the cytoplasmic and chromatin-enriched fractions, respectively.

### Purification of centrosomes from mammalian cells

Isolation and purification of centrosomes from KE37 cells was performed according to the procedure described by Gogendeau et al. (2015).

### Western blotting analysis

Proteins were quantified with the BCA protein assay kit (Pierce, 23225) and separated by SDS-PAGE to be transferred onto a polyvinylidene fluoride (PVDF) membrane. After blocking of non-specific binding sites, proteins were detected with primary antibodies and HRP-conjugated secondary antibodies (Table S3). Finally, membranes were developed using chemiluminescence detection. HSC70, HSP90 or β-actin was used as the loading control. BlueEasy Prestained Protein Marker (Nippon Genetics) was used as the molecular mass ladder. Blot transparency images are shown in Fig. S6.

### Co-IP analysis

U2OS cells were washed with ice-cold PBS, lysed with lysis buffer [20 mM Tris-HCl pH 7.5, 150 mM NaCl, 5 mM EDTA, 5 mM EGTA, 1% Triton X-100, complete EDTA-free protease inhibitor (Sigma-Aldrich, 11697498001) and phosphatase inhibitor (Sigma-Aldrich, 04906845001)]. Collected cells were sonicated and then incubated on ice for 30 min. The supernatant was clarified by centrifugation at 5000 ***g*** for 10 min at 4°C. After quantification, protein extracts were diluted to equal concentrations and then pre-cleared with protein A/G agarose beads (Santa Cruz Biotechnology, sc-2003). After centrifugation at 5000 ***g*** for 5 min at 4°C, the supernatants were incubated on the rotor overnight at 4°C with the indicated antibodies (Table S4). IgGs of the corresponding species were used as negative controls. A/G beads were then added to the lysates for 2 h at 4°C. After centrifugation (600 ***g*** for 10 min at 4°C), the beads were collected and washed four times with lysis buffer, resuspended in 2× Laemmli buffer, boiled for 10 min at 95°C and centrifuged at 5000 ***g*** for 5 min. Purified proteins were resolved by SDS-PAGE and subjected to western blotting analysis.

### Purification of GST fusion proteins

For GST fusion proteins, GST–DPF3a and GST–DPF3b were constructed by eZyvec and expressed in *Escherichia coli* BL21 cells (Stratagene, 200131). Logarithmic phase BL21 cells were induced by IPTG (1 mM) and cultured overnight at 16°C. Cells were harvested and then lysed in IPLS buffer (0.5% NP-40, 50 mM Tris-HCl, 120 mM NaCl, 0.5 mM EDTA, pH 8, and 10% glycerol) for 30 min at 4°C. Cell lysates were then subjected to centrifugation for 10 min at 10,000 ***g***. Fusion proteins were purified by glutathione-conjugated sepharose 4B beads (GE Healthcare).

### GST pull-down assay

Protein lysates from mock or transfected U2OS cell lysates were incubated with GST recombinant proteins immobilized on glutathione-conjugated sepharose beads at 4°C for 2 h. The beads were extensively washed and eluted proteins separated by SDS-PAGE were analyzed by western blotting using the indicated antibodies.

### Microtubule co-sedimentation assay

Microtubule co-sedimentation assays were performed using the Microtubule-Binding Protein Spin-down Assay Kit (Cytoskeleton, BK029) as described by the manufacturer. Briefly, preparations of GST–DPF3a and GST–DPF3b were centrifuged at 100,000 ***g*** (Beckman SW60Ti rotor) for 30 min at 4°C to remove aggregates of insoluble material. The supernatants were incubated with 2 µM taxol-stabilized microtubules prepared from 50 µM tubulin for 30 min at room temperature. The solutions were then subjected to centrifugation at 100,000 ***g*** for 40 min at room temperature to pellet microtubules with bound proteins. After solubilization of the pellet fraction, equal volumes of the pellet and supernatant fractions were resolved by SDS-PAGE, followed by Coomassie blue staining.

### Immunofluorescence

Cells were seeded on coverslips in 24-well plates. Cells were rinsed with PBS, permeabilized or not with ice-cold CSK buffer (300 mM sucrose, 3 mM MgCl_2_, 10 mM PIPES pH 7.0, 100 mM NaCl, 0.25% Triton X-100) to remove soluble protein and then fixed with cold methanol and acetone in a 80:20 ratio for 10 min at −20°C. After several washes, the coverslips were blocked in PBS with 1% BSA (PBS-BSA) for 30 min at room temperature and incubated with primary antibodies (Table S5) diluted in PBS-BSA for 2 h at room temperature. The coverslips were then washed and incubated with appropriate Alexa Fluor-conjugated secondary antibodies diluted in PBS-BSA for 1 h at room temperature. Nuclear cells were counterstained with 1 mg/ml DAPI (Life Technologies, D1306) for 10 min at room temperature in the dark. The coverslips were mounted onto microscope slides and images were obtained with a Leica TCS SP5 laser scanning confocal microscope or a Zeiss LSM 880 Airyscan 1 high-resolution microscope with a 63×/1.25 NA objective as indicated in the legend.

To quantify PCM1 dispersion, a circle at the basis of cilia was drawn in the control condition (‘No si’). In DPF3-depleted cells, the circle was placed in the area where the signal was still the brightest. The pixel density of anti-PCM1 staining in the corresponding area was measured using ImageJ. The fluorescence intensity was quantified and the signal level in control cells (‘No si’) was normalized to 1.

### Cold-stable microtubule assay

Briefly, U2OS cells were blocked in metaphase by treating them with RO-3306 (9 µM, 45 min), followed by MG132 (10 µM, 30 min). Cells were then incubated in ice-cold Leibovitz L-15 medium (Thermo Fisher Scientific, 21083) supplemented with 20 mM HEPES (pH 7.0) for 10 min. Cells were fixed with 4% paraformaldehyde in 100 mM PIPES, 25 mM HEPES, 10 mM EGTA, 1 mM MgCl_2_ and 0.2% Triton X-100. Immunofluorescence was performed as described above. Images were captured using the Zeiss LSM 880 Airyscan 1 high-resolution microscope with a 63×/1.25 NA objective controlled by Zeiss Zen imaging software. ImageJ was used to quantify fluorescence intensities.

### Live-cell imaging

After transfection, U2OS cells were plated in a cell culture dish with two individual compartments. Live imaging for 24 h with a time lapse of 5 min was performed with a Nikon A1R microscope. ImageJ was used for image analysis.

### TEM

Cells grown on glass slides were fixed in a 1.6% glutaraldehyde solution in 0.1 M sodium phosphate buffer (pH 7.4) at room temperature and then stored at 4°C. After three rinses in 0.1 M cacodylate buffer (pH 7.4) (15 min each), samples were postfixed in a 1% osmium tetroxide and 1% potassium ferrocyanide solution in 0.1 M cacodylate buffer for 1 h at room temperature. Cells were subsequently rinsed in double-distilled water and dehydrated in a series of ethanol baths (96%, 100% three times, 15 min each) and then progressively embedded in Agar 100 resin (1:1 ethanol:resin, 100% resin two times, 2 h for each bath). Resin blocs were finally left to harden at 60°C in an oven for 2 days. After removing the glass slides, ultrathin serial sections (70 nm) were obtained with a Reichert Ultracut S ultramicrotome equipped with a Diatome diamond knife and collected on copper slot grids with a formvar support film. Sections were stained with lead citrate and uranyl acetate. TEM observations were performed with a JEOL JEM-1400 transmission electron microscope, equipped with a Morada camera, at a 100 kV acceleration voltage.

### Cell cycle analysis

Cells were trypsinized and washed two times with 1× PBS. They were then fixed with ice-cold 70% ethanol and stored at −20°C until staining with propidium iodide (PI). Cells were centrifuged for 5 min at 800 ***g*** and washed two times with 1× PBS. Next, cells were resuspended in 300 µl of PI working solution (50 µg/ml of PI and 50 µg/ml of RNase A in PBS), then incubated for 30 min in the dark and analyzed by flow cytometry using a FACS Calibur II (BD Biosciences), Modfit ModFit LT software version 3.3 (Verity Software House) and CellQuest version 3.2 (BD Biosciences). A total of 10,000 live, single cells were recorded for each sample. Total cells were first gated according to their intrinsic size (forward scatter, FSC) and granularity (side scatter, SSC) properties on a log scale. The gate excluded cellular debris from living cells. Living cells were then gated based on the area (FL2-A) and the width (FL2-W) of the signal for DNA staining to exclude doublets.

### Apoptosis analysis

Apoptosis was measured with the FITC-Annexin V apoptosis Detection Kit I (BD Biosciences, 556547) according to the manufacturer's instructions. Flow cytometry was performed and data were analyzed with CellQuest software (BD Biosciences). Annexin V^+^/PI^−^ and Annexin V^+^/PI^+^ cells were considered apoptotic.

### Growth curve analysis

After transfection, U2OS cells were trypsinized and viable cells were plated at a density of 100,000 cells per well in a six-well plate. Cell counting was carried out every 24 h for 4 days, and the medium was replaced every 2 days.

### Statistical analysis

Unless otherwise indicated, data are expressed as mean±s.d. Statistical tests used were indicated in each figure legend. *P*-values less than 0.05 were considered statistically significant. *P*-values (relative to siCtrl) are displayed as following: *P<0.05; **P<0.01; ***P<0.001, ****P<0.0001. Exact *P*-values are indicated in each graph.

## Supplementary Material



10.1242/joces.261744_sup1 Supplementary information

## References

[JCS261744C1] Amin, M. A., Itoh, G., Iemura, K., Ikeda, M. and Tanaka, K. (2014). CLIP-170 recruits PLK1 to kinetochores during early mitosis for chromosome alignment. *J. Cell Sci.* 127, 2818-2824. 10.1242/jcs.150755 24777477

[JCS261744C2] Arlt, M. F., Xu, B., Durkin, S. G., Casper, A. M., Kastan, M. B. and Glover, T. W. (2004). BRCA1 is required for common-fragile-site stability via its G_2_/M checkpoint function. *Mol. Cell. Biol.* 24, 6701-6709. 10.1128/MCB.24.15.6701-6709.2004 15254237 PMC444841

[JCS261744C3] Asiedu, M., Wu, D., Matsumura, F. and Wei, Q. (2008). Phosphorylation of MyoGEF on Thr-574 by Plk1 promotes MyoGEF localization to the central spindle. *J. Biol. Chem.* 283, 28392-28400. 10.1074/jbc.M801801200 18694934 PMC2568926

[JCS261744C4] Batman, U., Deretic, J. and Firat-Karalar, E. N. (2022). The ciliopathy protein CCDC66 controls mitotic progression and cytokinesis by promoting microtubule nucleation and organization. *PLoS Biol.* 20, e3001708. 10.1371/journal.pbio.3001708 35849559 PMC9333452

[JCS261744C5] Baumann, K. (2012). Cell cycle: order in the pericentriolar material. *Nat. Rev. Mol. Cell Biol.* 13, 749. 10.1038/nrm3471 23095798

[JCS261744C6] Bendre, S., Rondelet, A., Hall, C., Schmidt, N., Lin, Y.-C., Brouhard, G. J. and Bird, A. W. (2016). GTSE1 tunes microtubule stability for chromosome alignment and segregation by inhibiting the microtubule depolymerase MCAK. *J. Cell Biol.* 215, 631-647. 10.1083/jcb.201606081 27881713 PMC5147003

[JCS261744C7] Brugmann, S. A., Cordero, D. R. and Helms, J. A. (2010). Craniofacial ciliopathies: a new classification for craniofacial disorders. *Am. J. Med. Genet. A* 152A, 2995-3006. 10.1002/ajmg.a.33727 21108387 PMC3121325

[JCS261744C8] Carmena, M., Wheelock, M., Funabiki, H. and Earnshaw, W. C. (2012). The chromosomal passenger complex (CPC): from easy rider to the godfather of mitosis. *Nat. Rev. Mol. Cell Biol.* 13, 789-803. 10.1038/nrm3474 23175282 PMC3729939

[JCS261744C9] Chambers, A. L., Ormerod, G., Durley, S. C., Sing, T. L., Brown, G. W., Kent, N. A. and Downs, J. A. (2012). The INO80 chromatin remodeling complex prevents polyploidy and maintains normal chromatin structure at centromeres. *Genes Dev.* 26, 2590-2603. 10.1101/gad.199976.112 23207916 PMC3521627

[JCS261744C10] Chu, Y., Yao, P. Y., Wang, W., Wang, D., Wang, Z., Zhang, L., Huang, Y., Ke, Y., Ding, X. and Yao, X. (2011). Aurora B kinase activation requires survivin priming phosphorylation by PLK1. *J. Mol. Cell Biol.* 3, 260-267. 10.1093/jmcb/mjq037 21148584 PMC3150119

[JCS261744C11] Ciossani, G., Overlack, K., Petrovic, A., Huis in ‘t Veld, P. J., Koerner, C., Wohlgemuth, S., Maffini, S. and Musacchio, A. (2018). The kinetochore proteins CENP-E and CENP-F directly and specifically interact with distinct BUB mitotic checkpoint Ser/Thr kinases. *J. Biol. Chem.* 293, 10084-10101. 10.1074/jbc.RA118.003154 29748388 PMC6028960

[JCS261744C12] Combes, G., Alharbi, I., Braga, L. G. and Elowe, S. (2017). Playing polo during mitosis: PLK1 takes the lead. *Oncogene* 36, 4819-4827. 10.1038/onc.2017.113 28436952

[JCS261744C13] Conkar, D., Bayraktar, H. and Firat-Karalar, E. N. (2019). Centrosomal and ciliary targeting of CCDC66 requires cooperative action of centriolar satellites, microtubules and molecular motors. *Sci. Rep.* 9, 14250. 10.1038/s41598-019-50530-4 31582766 PMC6776500

[JCS261744C14] Cui, H., Schlesinger, J., Schoenhals, S., Tönjes, M., Dunkel, I., Meierhofer, D., Cano, E., Schulz, K., Berger, M. F., Haack, T. et al. (2016). Phosphorylation of the chromatin remodeling factor DPF3a induces cardiac hypertrophy through releasing HEY repressors from DNA. *Nucleic Acids Res.* 44, 2538-2553. 10.1093/nar/gkv1244 26582913 PMC4824069

[JCS261744C15] Cusan, M., Shen, H., Zhang, B., Liao, A., Yang, L., Jin, M., Fernandez, M., Iyer, P., Wu, Y., Hart, K. et al. (2023). SF3B1 mutation and ATM deletion codrive leukemogenesis via centromeric R-loop dysregulation. *J. Clin. Invest.* 133, e163325. 10.1172/JCI163325 37463047 PMC10471171

[JCS261744C16] de Pontual, L., Pelet, A., Clement-Ziza, M., Trochet, D., Antonarakis, S. E., Attie-Bitach, T., Beales, P. L., Blouin, J.-L., Dastot-Le Moal, F., Dollfus, H. et al. (2007). Epistatic interactions with a common hypomorphic RET allele in syndromic Hirschsprung disease. *Hum. Mutat.* 28, 790-796. 10.1002/humu.20517 17397038

[JCS261744C17] Ditchfield, C., Johnson, V. L., Tighe, A., Ellston, R., Haworth, C., Johnson, T., Mortlock, A., Keen, N. and Taylor, S. S. (2003). Aurora B couples chromosome alignment with anaphase by targeting BubR1, Mad2, and Cenp-E to kinetochores. *J. Cell Biol.* 161, 267-280. 10.1083/jcb.200208091 12719470 PMC2172902

[JCS261744C18] Dudka, D., Castrogiovanni, C., Liaudet, N., Vassal, H. and Meraldi, P. (2019). Spindle-length-dependent HURP localization allows centrosomes to control kinetochore-fiber plus-end dynamics. *Curr. Biol.* 29, 3563-3578.e6. 10.1016/j.cub.2019.08.061 31668617

[JCS261744C19] Emanuele, M. J. and Stukenberg, P. T. (2007). Xenopus Cep57 is a novel kinetochore component involved in microtubule attachment. *Cell* 130, 893-905. 10.1016/j.cell.2007.07.023 17803911

[JCS261744C20] Fabbro, M., Zhou, B.-B., Takahashi, M., Sarcevic, B., Lal, P., Graham, M. E., Gabrielli, B. G., Robinson, P. J., Nigg, E. A., Ono, Y. et al. (2005). Cdk1/Erk2- and Plk1-dependent phosphorylation of a centrosome protein, Cep55, is required for its recruitment to midbody and cytokinesis. *Dev. Cell* 9, 477-488. 10.1016/j.devcel.2005.09.003 16198290

[JCS261744C21] Firat-Karalar, E. N., Sante, J., Elliott, S. and Stearns, T. (2014). Proteomic analysis of mammalian sperm cells identifies new components of the centrosome. *J. Cell Sci.* 127, 4128-4133. 10.1242/jcs.157008 25074808 PMC4179487

[JCS261744C22] Gabriel, G. C., Young, C. B. and Lo, C. W. (2021). Role of cilia in the pathogenesis of congenital heart disease. *Semin. Cell Dev. Biol.* 110, 2-10. 10.1016/j.semcdb.2020.04.017 32418658 PMC7906359

[JCS261744C23] Giles, K. A., Gould, C. M., Achinger-Kawecka, J., Page, S. G., Kafer, G. R., Rogers, S., Luu, P.-L., Cesare, A. J., Clark, S. J. and Taberlay, P. C. (2021). BRG1 knockdown inhibits proliferation through multiple cellular pathways in prostate cancer. *Clin. Epigenet.* 13, 37. 10.1186/s13148-021-01023-7 PMC7888175 33596994

[JCS261744C24] Gogendeau, D., Guichard, P. and Tassin, A.-M. (2015). Purification of centrosomes from mammalian cell lines. *Methods Cell Biol.* 129, 171-189. 10.1016/bs.mcb.2015.03.004 26175439

[JCS261744C25] Goranci-Buzhala, G., Mariappan, A., Ricci-Vitiani, L., Josipovic, N., Pacioni, S., Gottardo, M., Ptok, J., Schaal, H., Callaini, G., Rajalingam, K. et al. (2021). Cilium induction triggers differentiation of glioma stem cells. *Cell Rep.* 36, 109656. 10.1016/j.celrep.2021.109656 34496239

[JCS261744C26] Hall, E. A., Keighren, M., Ford, M. J., Davey, T., Jarman, A. P., Smith, L. B., Jackson, I. J. and Mill, P. (2013). Acute versus chronic loss of mammalian Azi1/Cep131 results in distinct ciliary phenotypes. *PLoS Genet.* 9, e1003928. 10.1371/journal.pgen.1003928 24415959 PMC3887133

[JCS261744C27] Hall, E. A., Kumar, D., Prosser, S. L., Yeyati, P. L., Herranz-Pérez, V., García-Verdugo, J. M., Rose, L., McKie, L., Dodd, D. O., Tennant, P. A. et al. (2023). Centriolar satellites expedite mother centriole remodeling to promote ciliogenesis. *eLife* 12, e79299. 10.7554/eLife.79299 36790165 PMC9998092

[JCS261744C89] Han, P., Hang, C. T., Yang, J. and Chang, C. P. (2011). Chromatin remodeling in cardiovascular development and physiology. *Circ. Res.* 108, 378-396. 10.1161/CIRCRESAHA.110.224287 21293009 PMC3079363

[JCS261744C28] Hannah, W. B., DeBrosse, S., Kinghorn, B. A., Strausbaugh, S., Aitken, M. L., Rosenfeld, M., Wolf, W. E., Knowles, M. R. and Zariwala, M. A. (2019). The expanding phenotype of *OFD1* -related disorders: Hemizygous loss–of–function variants in three patients with primary ciliary dyskinesia. *Mol. Genet. Genomic Med.* 7, e911. 10.1002/mgg3.911 31373179 PMC6732318

[JCS261744C29] Hiramatsu, H., Kobayashi, K., Kobayashi, K., Haraguchi, T., Ino, Y., Todo, T. and Iba, H. (2017). The role of the SWI/SNF chromatin remodeling complex in maintaining the stemness of glioma initiating cells. *Sci. Rep.* 7, 889. 10.1038/s41598-017-00982-3 28420882 PMC5429847

[JCS261744C30] Ho, L. and Crabtree, G. R. (2010). Chromatin remodelling during development. *Nature* 463, 474-484. 10.1038/nature08911 20110991 PMC3060774

[JCS261744C31] Hoang-Minh, L. B., Dutra-Clarke, M., Breunig, J. J. and Sarkisian, M. R. (2018). Glioma cell proliferation is enhanced in the presence of tumor-derived cilia vesicles. *Cilia* 7, 6. 10.1186/s13630-018-0060-5 30410731 PMC6219037

[JCS261744C32] Hur, S.-K., Park, E.-J., Han, J.-E., Kim, Y.-A., Kim, J.-D., Kang, D. and Kwon, J. (2010). Roles of human INO80 chromatin remodeling enzyme in DNA replication and chromosome segregation suppress genome instability. *Cell. Mol. Life Sci.* 67, 2283-2296. 10.1007/s00018-010-0337-3 20237820 PMC11115786

[JCS261744C33] Jagrić, M., Risteski, P., Martinčić, J., Milas, A. and Tolić, I. M. (2021). Optogenetic control of PRC1 reveals its role in chromosome alignment on the spindle by overlap length-dependent forces. *eLife* 10, e61170. 10.7554/eLife.61170 33480356 PMC7924949

[JCS261744C34] Janssen, L. M. E., Averink, T. V., Blomen, V. A., Brummelkamp, T. R., Medema, R. H. and Raaijmakers, J. A. (2018). Loss of Kif18A results in spindle assembly checkpoint activation at microtubule-attached kinetochores. *Curr. Biol.* 28, 2685-2696.e4. 10.1016/j.cub.2018.06.026 30122526

[JCS261744C35] Jiang, X., Ho, D. B. T., Mahe, K., Mia, J., Sepulveda, G., Antkowiak, M., Jiang, L., Yamada, S. and Jao, L.-E. (2021). Condensation of pericentrin proteins in human cells illuminates phase separation in centrosome assembly. *J. Cell Sci.* 134, jcs258897. 10.1242/jcs.258897 34308971 PMC8349556

[JCS261744C36] Kadoch, C. and Crabtree, G. R. (2015). Mammalian SWI/SNF chromatin remodeling complexes and cancer: Mechanistic insights gained from human genomics. *Sci. Adv.* 1, e1500447. 10.1126/sciadv.1500447 26601204 PMC4640607

[JCS261744C37] Kajtez, J., Solomatina, A., Novak, M., Polak, B., Vukušić, K., Rüdiger, J., Cojoc, G., Milas, A., Šumanovac Šestak, I., Risteski, P. et al. (2016). Overlap microtubules link sister k-fibres and balance the forces on bi-oriented kinetochores. *Nat. Commun.* 7, 10298. 10.1038/ncomms10298 26728792 PMC4728446

[JCS261744C38] Kaynak, B., von Heydebreck, A., Mebus, S., Seelow, D., Hennig, S., Vogel, J., Sperling, H.-P., Pregla, R., Alexi-Meskishvili, V., Hetzer, R. et al. (2003). Genome-wide array analysis of normal and malformed human hearts. *Circulation* 107, 2467-2474. 10.1161/01.CIR.0000066694.21510.E2 12742993

[JCS261744C39] Kim, Y., Holland, A. J., Lan, W. and Cleveland, D. W. (2010). Aurora kinases and protein phosphatase 1 mediate chromosome congression through regulation of CENP-E. *Cell* 142, 444-455. 10.1016/j.cell.2010.06.039 20691903 PMC2921712

[JCS261744C40] Kim, J. H., Shim, J., Ji, M.-J., Jung, Y., Bong, S. M., Jang, Y.-J., Yoon, E.-K., Lee, S.-J., Kim, K. G., Kim, Y. H. et al. (2014). The condensin component NCAPG2 regulates microtubule-kinetochore attachment through recruitment of Polo-like kinase 1 to kinetochores. *Nat. Commun.* 5, 4588. 10.1038/ncomms5588 25109385

[JCS261744C41] Klena, N. T., Gibbs, B. C. and Lo, C. W. (2017). Cilia and ciliopathies in congenital heart disease. *Cold Spring Harb. Perspect. Biol.* 9, a028266. 10.1101/cshperspect.a028266 28159874 PMC5538412

[JCS261744C42] Kosova, G., Hotaling, J. M., Ohlander, S., Niederberger, C., Prins, G. S. and Ober, C. (2014). Variants in DPF3 and DSCAML1 are associated with sperm morphology. *J. Assist. Reprod. Genet.* 31, 131-137. 10.1007/s10815-013-0140-9 24271036 PMC3933604

[JCS261744C43] Kubo, A., Sasaki, H., Yuba-Kubo, A., Tsukita, S. and Shiina, N. (1999). Centriolar satellites: molecular characterization, ATP-dependent movement toward centrioles and possible involvement in ciliogenesis. *J. Cell Biol.* 147, 969-980. 10.1083/jcb.147.5.969 10579718 PMC2169353

[JCS261744C44] Kuhns, S., Schmidt, K. N., Reymann, J., Gilbert, D. F., Neuner, A., Hub, B., Carvalho, R., Wiedemann, P., Zentgraf, H., Erfle, H. et al. (2013). The microtubule affinity regulating kinase MARK4 promotes axoneme extension during early ciliogenesis. *J. Cell Biol.* 200, 505-522. 10.1083/jcb.201206013 23400999 PMC3575539

[JCS261744C45] Kurtulmus, B., Yuan, C., Schuy, J., Neuner, A., Hata, S., Kalamakis, G., Martin-Villalba, A. and Pereira, G. (2018). LRRC45 contributes to early steps of axoneme extension. *J. Cell Sci.* 131, jcs223594. 10.1242/jcs.223594 30131441

[JCS261744C46] Lange, M., Kaynak, B., Forster, U. B., Tönjes, M., Fischer, J. J., Grimm, C., Schlesinger, J., Just, S., Dunkel, I., Krueger, T. et al. (2008). Regulation of muscle development by DPF3, a novel histone acetylation and methylation reader of the BAF chromatin remodeling complex. *Genes Dev.* 22, 2370-2384. 10.1101/gad.471408 18765789 PMC2532927

[JCS261744C47] Lee, H.-S., Min, S., Jung, Y.-E., Chae, S., Heo, J., Lee, J.-H., Kim, T. S., Kang, H.-C., Nakanish, M., Cha, S.-S. et al. (2021). Spatiotemporal coordination of the RSF1-PLK1-Aurora B cascade establishes mitotic signaling platforms. *Nat. Commun.* 12, 5931. 10.1038/s41467-021-26220-z 34635673 PMC8505570

[JCS261744C48] Lessard, J., Wu, J. I., Ranish, J. A., Wan, M., Winslow, M. M., Staahl, B. T., Wu, H., Aebersold, R., Graef, I. A. and Crabtree, G. R. (2007). An essential switch in subunit composition of a chromatin remodeling complex during neural development. *Neuron* 55, 201-215. 10.1016/j.neuron.2007.06.019 17640523 PMC2674110

[JCS261744C49] Leyder, T., Mignon, J., Mottet, D. and Michaux, C. (2022). Unveiling the metal-dependent aggregation properties of the C-terminal region of amyloidogenic intrinsically disordered protein isoforms DPF3b and DPF3a. *Int. J. Mol. Sci.* 23, 15291. 10.3390/ijms232315291 36499617 PMC9738585

[JCS261744C50] Liu, H., Luo, Y., Li, S., Wang, S., Wang, N. and Jin, X. (2014). Expression profiles of HA117 and its neighboring gene DPF3 in different colon segments of Hirschsprung's disease. *Int. J. Clin. Exp. Pathol.* 7, 3966-3974. 25120773 PMC4129008

[JCS261744C51] Liu, S.-Y., Zhang, C.-J., Peng, H.-Y., Sun, H., Lin, K.-Q., Huang, X.-Q., Huang, K., Chu, J.-Y. and Yang, Z.-Q. (2017). Strong association of SLC1A1 and DPF3 gene variants with idiopathic male infertility in Han Chinese. *Asian J. Androl.* 19, 486-492. 10.4103/1008-682X.178850 27232852 PMC5507099

[JCS261744C52] Liu, X., Liu, X., Wang, H., Dou, Z., Ruan, K., Hill, D. L., Li, L., Shi, Y. and Yao, X. (2020). Phase separation drives decision making in cell division. *J. Biol. Chem.* 295, 13419-13431. 10.1074/jbc.REV120.011746 32699013 PMC7521646

[JCS261744C53] Lopes, C. A. M., Prosser, S. L., Romio, L., Hirst, R. A., O'Callaghan, C., Woolf, A. S. and Fry, A. M. (2011). Centriolar satellites are assembly points for proteins implicated in human ciliopathies, including oral-facial-digital syndrome 1. *J. Cell Sci.* 124, 600-612. 10.1242/jcs.077156 21266464 PMC3031371

[JCS261744C54] Maia, A. R. R., Garcia, Z., Kabeche, L., Barisic, M., Maffini, S., Macedo-Ribeiro, S., Cheeseman, I. M., Compton, D. A., Kaverina, I. and Maiato, H. (2012). Cdk1 and Plk1 mediate a CLASP2 phospho-switch that stabilizes kinetochore–microtubule attachments. *J. Cell Biol.* 199, 285-301. 10.1083/jcb.201203091 23045552 PMC3471233

[JCS261744C91] Méndez, J. and Stillman, B. (2000). Chromatin association of human origin recognition complex, cdc6, and minichromosome maintenance proteins during the cell cycle: assembly of prereplication complexes in late mitosis. *Mol. Cell. Biol.* 20, 8602-8612. 10.1128/MCB.20.22.8602-8612.2000 11046155 PMC102165

[JCS261744C55] Messina, G., Prozzillo, Y., Monache, F. D., Santopietro, M. V. and Dimitri, P. (2022). Evolutionary conserved relocation of chromatin remodeling complexes to the mitotic apparatus. *BMC Biol.* 20, 172. 10.1186/s12915-022-01365-5 35922843 PMC9351137

[JCS261744C56] Mignon, J., Mottet, D., Verrillo, G., Matagne, A., Perpète, E. A. and Michaux, C. (2021). Revealing intrinsic disorder and aggregation properties of the DPF3a zinc finger protein. *ACS Omega* 6, 18793-18801. 10.1021/acsomega.1c01948 34337219 PMC8319922

[JCS261744C57] Mignon, J., Mottet, D., Leyder, T., Uversky, V. N., Perpète, E. A. and Michaux, C. (2022). Structural characterisation of amyloidogenic intrinsically disordered zinc finger protein isoforms DPF3b and DPF3a. *Int. J. Biol. Macromol.* 218, 57-71. 10.1016/j.ijbiomac.2022.07.102 35863661

[JCS261744C58] Muchardt, C., Reyes, J. C., Bourachot, B., Leguoy, E. and Yaniv, M. (1996). The hbrm and BRG-1 proteins, components of the human SNF/SWI complex, are phosphorylated and excluded from the condensed chromosomes during mitosis. *EMBO J.* 15, 3394-3402. 10.1002/j.1460-2075.1996.tb00705.x 8670841 PMC451903

[JCS261744C59] Niedzialkowska, E., Liu, L., Kuscu, C., Mayo, Z., Minor, W., Strahl, B. D., Adli, M. and Stukenberg, P. T. (2022). Tip60 acetylation of histone H3K4 temporally controls chromosome passenger complex localization. *Mol. Biol. Cell* 33, br15. 10.1091/mbc.E21-06-0283 35653296 PMC9582641

[JCS261744C60] Niedzialkowska, E., Truong, T. M., Eldredge, L. A., Ali, A., Redemann, S. and Stukenberg, P. T. (2024). Chromosomal passenger complex condensates generate parallel microtubule bundles in vitro. *J. Biol. Chem.* 300, 105669. 10.1016/j.jbc.2024.105669 38272221 PMC10876603

[JCS261744C61] Nishino, M., Kurasawa, Y., Evans, R., Lin, S.-H., Brinkley, B. R. and Yu-Lee, L.-Y. (2006). NudC is required for Plk1 targeting to the kinetochore and chromosome congression. *Curr. Biol.* 16, 1414-1421. 10.1016/j.cub.2006.05.052 16860740

[JCS261744C62] Papon, J. F., Perrault, I., Coste, A., Louis, B., Gérard, X., Hanein, S., Fares-Taie, L., Gerber, S., Defoort-Dhellemmes, S., Vojtek, A. M. et al. (2010). Abnormal respiratory cilia in non-syndromic Leber congenital amaurosis with CEP290 mutations. *J. Med. Genet.* 47, 829-834. 10.1136/jmg.2010.077883 20805370

[JCS261744C63] Raemaekers, T., Ribbeck, K., Beaudouin, J., Annaert, W., Van Camp, M., Stockmans, I., Smets, N., Bouillon, R., Ellenberg, J. and Carmeliet, G. (2003). NuSAP, a novel microtubule-associated protein involved in mitotic spindle organization. *J. Cell Biol.* 162, 1017-1029. 10.1083/jcb.200302129 12963707 PMC2172854

[JCS261744C64] Raff, J. W. (2019). Phase separation and the centrosome: a fait Accompli? *Trend. Cell Biol.* 29, 612-622. 10.1016/j.tcb.2019.04.001 31076235

[JCS261744C65] Salisbury, J. L., Suino, K. M., Busby, R. and Springett, M. (2002). Centrin-2 is required for centriole duplication in mammalian cells. *Curr. Biol.* 12, 1287-1292. 10.1016/S0960-9822(02)01019-9 12176356

[JCS261744C66] Sánchez-Molina, S., Mortusewicz, O., Bieber, B., Auer, S., Eckey, M., Leonhardt, H., Friedl, A. A. and Becker, P. B. (2011). Role for hACF1 in the G2/M damage checkpoint. *Nucleic Acids Res.* 39, 8445-8456. 10.1093/nar/gkr435 21745822 PMC3201854

[JCS261744C67] Sarkisian, M. R. and Semple-Rowland, S. L. (2019). Emerging roles of primary cilia in glioma. *Front. Cell Neurosci.* 13, 55. 10.3389/fncel.2019.00055 30842728 PMC6391589

[JCS261744C68] Sato, Y., Hasegawa, C., Tajima, A., Nozawa, S., Yoshiike, M., Koh, E., Kanaya, J., Namiki, M., Matsumiya, K., Tsujimura, A. et al. (2018). Association of TUSC1 and DPF3 gene polymorphisms with male infertility. *J. Assist. Reprod. Genet.* 35, 257-263. 10.1007/s10815-017-1052-x 28975488 PMC5845029

[JCS261744C69] Schick, S., Rendeiro, A. F., Runggatscher, K., Ringler, A., Boidol, B., Hinkel, M., Májek, P., Vulliard, L., Penz, T., Parapatics, K. et al. (2019). Systematic characterization of BAF mutations provides insights into intracomplex synthetic lethalities in human cancers. *Nat. Genet.* 51, 1399-1410. 10.1038/s41588-019-0477-9 31427792 PMC6952272

[JCS261744C70] Serena, M., Bastos, R. N., Elliott, P. R. and Barr, F. A. (2020). Molecular basis of MKLP2-dependent Aurora B transport from chromatin to the anaphase central spindle. *J. Cell Biol.* 219, e201910059. 10.1083/jcb.201910059 32356865 PMC7337490

[JCS261744C71] Sha, Y.-W., Xu, X., Mei, L.-B., Li, P., Su, Z.-Y., He, X.-Q. and Li, L. (2017). A homozygous CEP135 mutation is associated with multiple morphological abnormalities of the sperm flagella (MMAF). *Gene* 633, 48-53. 10.1016/j.gene.2017.08.033 28866084

[JCS261744C90] Staahl, B. T. and Crabtree, G. R. (2013). Creating a neural specific chromatin landscape by npBAF and nBAF complexes. *Curr. Opin. Neurobiol.* 23, 903-913. 10.1016/j.conb.2013.09.003 24090879 PMC3878911

[JCS261744C72] Sullivan, B. A. and Karpen, G. H. (2004). Centromeric chromatin exhibits a histone modification pattern that is distinct from both euchromatin and heterochromatin. *Nat. Struct. Mol. Biol.* 11, 1076-1083. 10.1038/nsmb845 15475964 PMC1283111

[JCS261744C73] Trivedi, P. and Stukenberg, P. T. (2020). A condensed view of the chromosome passenger complex. *Trends Cell Biol.* 30, 676-687. 10.1016/j.tcb.2020.06.005 32684321 PMC10714244

[JCS261744C74] Vieira, A. R., McHenry, T. G., Daack-Hirsch, S., Murray, J. C. and Marazita, M. L. (2008). Candidate gene/loci studies in cleft lip/palate and dental anomalies finds novel susceptibility genes for clefts. *Genet. Med.* 10, 668-674. 10.1097/GIM.0b013e3181833793 18978678 PMC2734954

[JCS261744C75] Vukušić, K., Buđa, R., Bosilj, A., Milas, A., Pavin, N. and Tolić, I. M. (2017). Microtubule sliding within the bridging fiber pushes kinetochore fibers apart to segregate chromosomes. *Dev. Cell* 43, 11-23.e6. 10.1016/j.devcel.2017.09.010 29017027 PMC5637169

[JCS261744C76] Vukušić, K., Ponjavić, I., Buđa, R., Risteski, P. and Tolić, I. M. (2021). Microtubule-sliding modules based on kinesins EG5 and PRC1-dependent KIF4A drive human spindle elongation. *Dev. Cell* 56, 1253-1267.e10. 10.1016/j.devcel.2021.04.005 33910056 PMC8098747

[JCS261744C77] Warren, J. D., Orr, B. and Compton, D. A. (2020). A comparative analysis of methods to measure kinetochore-microtubule attachment stability. *Methods Cell Biol.* 158, 91-116. 10.1016/bs.mcb.2020.01.004 32423652 PMC7727308

[JCS261744C78] Wei, R., Ngo, B., Wu, G. and Lee, W.-H. (2011). Phosphorylation of the Ndc80 complex protein, HEC1, by Nek2 kinase modulates chromosome alignment and signaling of the spindle assembly checkpoint. *Mol. Biol. Cell* 22, 3584-3594. 10.1091/mbc.e11-01-0012 21832156 PMC3183014

[JCS261744C79] Woodruff, J. B., Ferreira Gomes, B., Widlund, P. O., Mahamid, J., Honigmann, A. and Hyman, A. A. (2017). The centrosome is a selective condensate that nucleates microtubules by concentrating tubulin. *Cell* 169, 1066-1077.e10. 10.1016/j.cell.2017.05.028 28575670

[JCS261744C80] Yan, L., Xie, S., Du, Y. and Qian, C. (2017). Structural insights into BAF47 and BAF155 complex formation. *J. Mol. Biol.* 429, 1650-1660. 10.1016/j.jmb.2017.04.008 28438634

[JCS261744C81] Yang, Y., Roine, N. and Mäkelä, T. P. (2013). CCRK depletion inhibits glioblastoma cell proliferation in a cilium-dependent manner. *EMBO Rep.* 14, 741-747. 10.1038/embor.2013.80 23743448 PMC3736126

[JCS261744C82] Yokoyama, H., Rybina, S., Santarella-Mellwig, R., Mattaj, I. W. and Karsenti, E. (2009). ISWI is a RanGTP-dependent MAP required for chromosome segregation. *J. Cell Biol.* 187, 813-829. 10.1083/jcb.200906020 20008562 PMC2806316

[JCS261744C83] Yokoyama, H., Nakos, K., Santarella-Mellwig, R., Rybina, S., Krijgsveld, J., Koffa, M. D. and Mattaj, I. W. (2013). CHD4 is a RanGTP-dependent MAP that stabilizes microtubules and regulates bipolar spindle formation. *Curr. Biol.* 23, 2443-2451. 10.1016/j.cub.2013.09.062 24268414

[JCS261744C84] Zeng, L., Zhang, Q., Li, S. D., Plotnikov, A. N., Walsh, M. J. and Zhou, M.-M. (2010). Mechanism and regulation of acetylated histone binding by the tandem PHD finger of DPF3b. *Nature* 466, 258-262. 10.1038/nature09139 20613843 PMC2901902

[JCS261744C85] Zhao, H., Chen, Q., Li, F., Cui, L., Xie, L., Huang, Q., Liang, X., Zhou, J., Yan, X. and Zhu, X. (2021). Fibrogranular materials function as organizers to ensure the fidelity of multiciliary assembly. *Nat. Commun.* 12, 1273. 10.1038/s41467-021-21506-8 33627667 PMC7904937

[JCS261744C92] Zhao, H., Khan, Z. and Westlake, C. J. (2023). Ciliogenesis membrane dynamics and organization. *Semin. Cell Dev. Biol.* 133, 20-131. 10.1016/j.semcdb.2022.03.021 35351373 PMC9510604

[JCS261744C86] Zhou, H., Zheng, T., Wang, T., Li, Q., Wang, F., Liang, X., Chen, J. and Teng, J. (2019). CCDC74A/B are K-fiber crosslinkers required for chromosomal alignment. *BMC Biol.* 17, 73. 10.1186/s12915-019-0694-9 31521166 PMC6744678

[JCS261744C87] Zhu, G., Conner, S. E., Zhou, X., Shih, C., Li, T., Anderson, B. D., Brooks, H. B., Campbell, R. M., Considine, E., Dempsey, J. A. et al. (2003). Synthesis, structure-activity relationship, and biological studies of indolocarbazoles as potent cyclin D1-CDK4 inhibitors. *J. Med. Chem.* 46, 2027-2030. 10.1021/jm0256169 12747775

[JCS261744C88] Zhu, X., Lan, B., Yi, X., He, C., Dang, L., Zhou, X., Lu, Y., Sun, Y., Liu, Z., Bai, X. et al. (2020). HRP2-DPF3a-BAF complex coordinates histone modification and chromatin remodeling to regulate myogenic gene transcription. *Nucleic Acids Res.* 48, 6563-6582. 10.1093/nar/gkaa441 32459350 PMC7337902

