# Supplementary material for: Non-canonical role for the BAF complex subunit DPF3 in mitosis and ciliogenesis
Source: J Cell Sci. 2024 May 13;137(9):jcs261744. doi: 10.1242/jcs.261744 (PMC11166463; doi:10.1242/jcs.261744)
Supplement: Supplementary information [file joces-137-261744-s1.pdf]

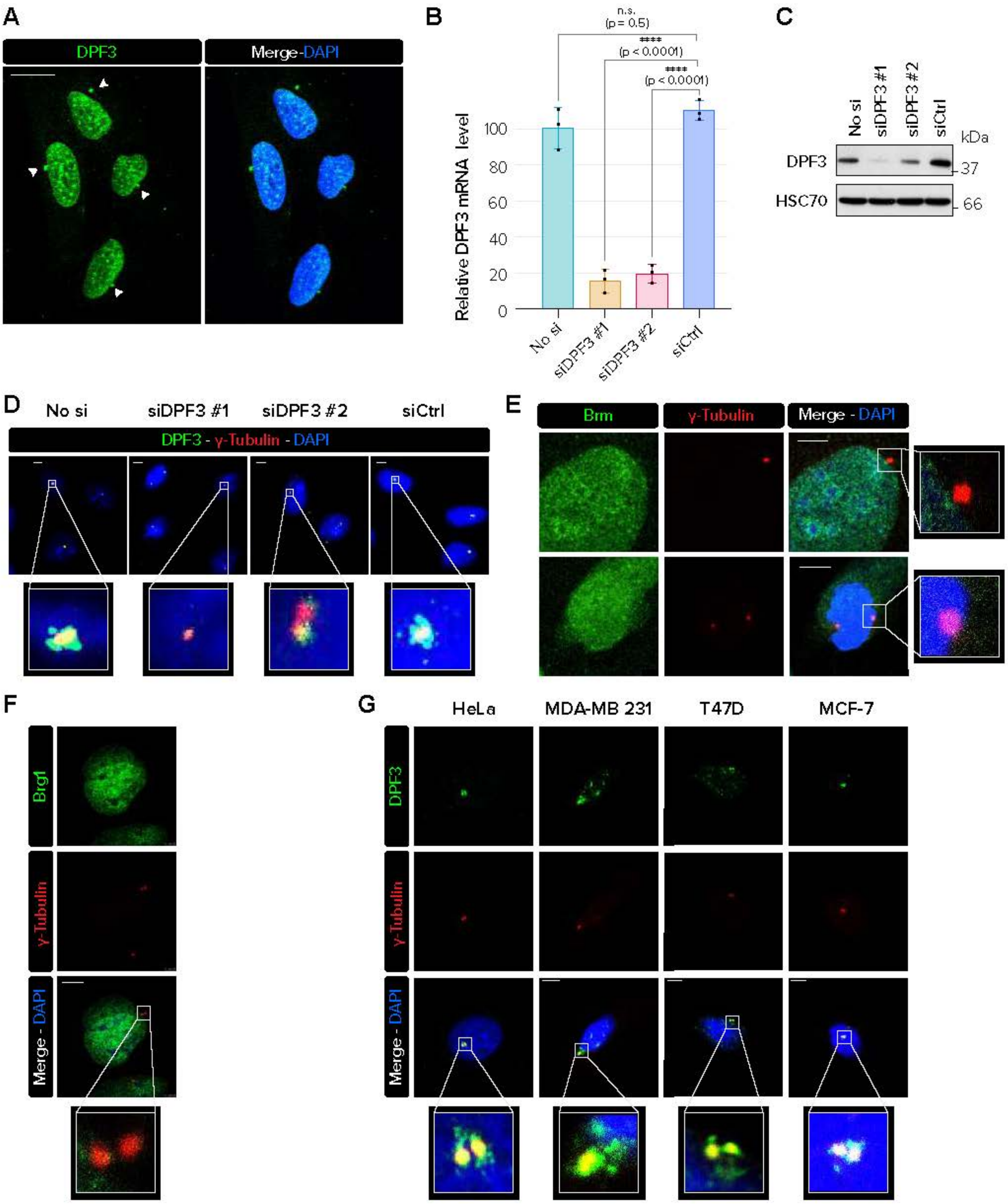

Fig. S1. Localization of DPF3 and other BAF subunits and specificity of the DPF3 staining at centrosome

(A) U2OS were fixed with methanol/acetone (80/20) and stained for DPF3. Several cells are shown and are representative of the total population. Individual channels for DPF3 (in green) and merged channels with DAPI nuclear staining (in blue) are shown. Scale bar = 10 µm. (**B-C**) U2OS

Journal of Cell Science • Supplementary information

cells were mock-transfected (No si) or transfected with one of two different DPF3 siRNAs (siDPF3 #1 and siDPF3 #2) or control siRNA (siCtrl). RNA and protein extracts were prepared in parallel and analyzed by RT-qPCR (**B**) or western blotting (**C**), respectively. (**D**) U2OS cells were mock-transfected (No si) or transfected with one of two different DPF3 siRNAs (siDPF3 #1 and siDPF3 #2) or control siRNA (siCtrl) and co-stained for DPF3 (in green) and γ-tubulin (in red). Merged channels with DAPI nuclear staining (in blue) and magnification of boxed regions are shown. Scale bar = 5 µm. (**E**) U2OS cells co-stained for Brm (in green) and γ-tubulin (in red). Individual channels, merged channels with DAPI nuclear staining (in blue) and magnification of boxed regions are shown. Scale bar = 10 µm. (**F**) U2OS cells co-stained for Brg1 (in green) and γ-tubulin (in red). Individual channels, merged channels with DAPI nuclear staining (in blue) and magnification of boxed regions are shown. Scale bar = 5 µm. (**G**) DPF3 (in green) and γ-tubulin (in red) were co-stained in HeLa, MDA-MB 231, T47D and MCF-7 (from top to bottom). Individual channels, merged channels with DAPI nuclear staining (in blue) and magnification of boxed regions are shown. Scale bar = 5 µm.

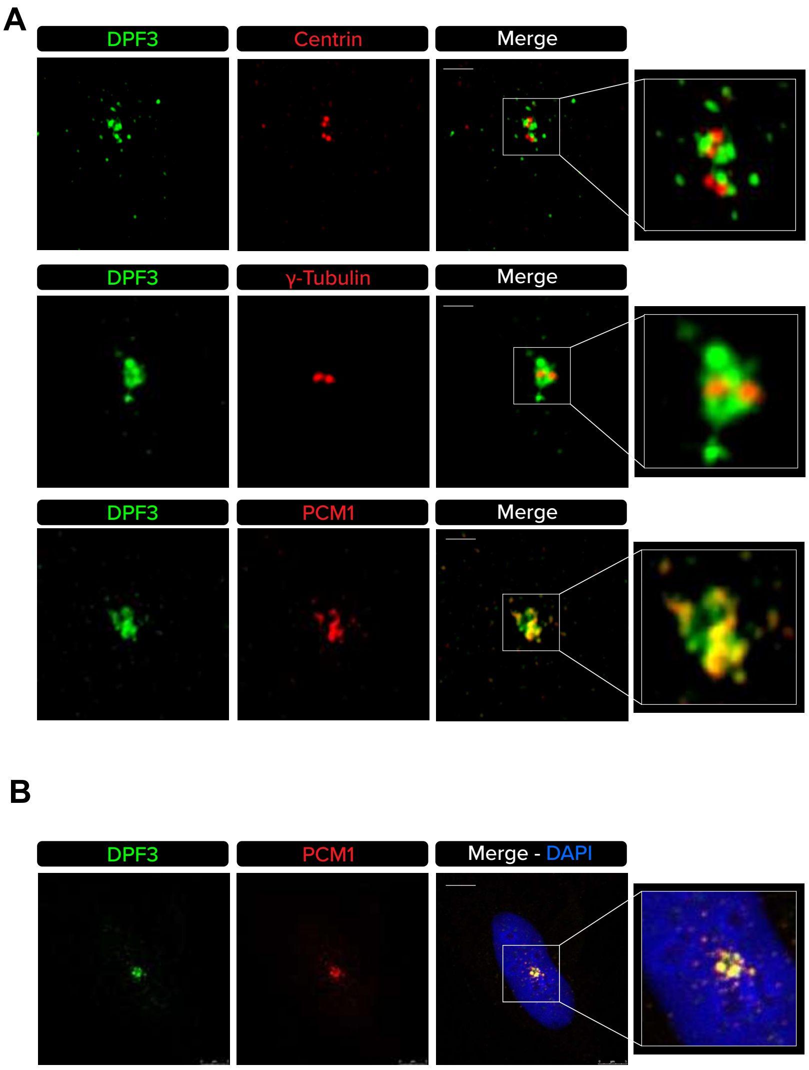

Fig. S2. Co-localization of DPF3 with centriolar satellites in HeLa cells

(**A**) HeLa cells co-stained for DPF3 (in green) and centrin, γ-tubulin or PCM1 (in red). Individual channels, merged channels and magnification of boxed regions are shown. Scale bar = 2 μm. Image was captured with Zeiss LSM 880 Airyscan High Resolution (HR) microscope. (**B**) HeLa cells were co-stained for DPF3 (in green) and PCM1 (in red). Individual channels, merged channels with DAPI nuclear staining (in blue), and magnification of boxed regions are shown. Scale bar = 5 μm. Image was captured with Leica TCS SP5 laser scanning confocal microscope.

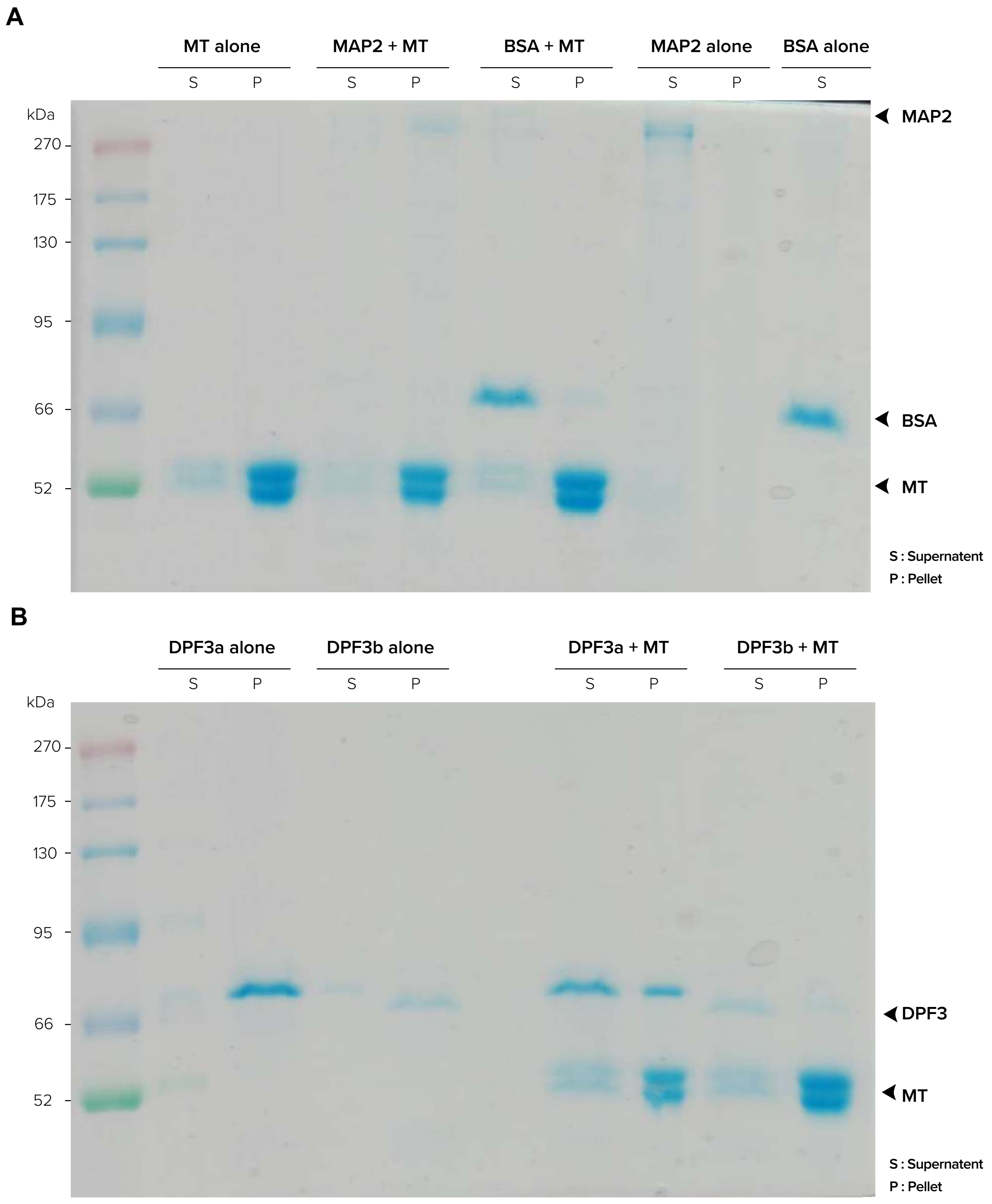

Fig. S3. DPF3a and to a lesser extend DPF3b interact with microtubules

(**A**) Taxol-stabilized microtubules were incubated or not with purified MAP2 (positive control) or BSA (negative control) and sedimented by ultracentrifugation. The supernatant (S) and pellet (P) fractions were run on a SDS gel and stained with Coomassie blue. **(B)** Taxol-stabilized microtubules were incubated or not with purified GST-DPF3a or GST-DPF3b and sedimented by ultracentrifugation. The supernatant (S) and pellet (P) fractions were analyzed by SDS-PAGE followed by Coomassie blue staining.

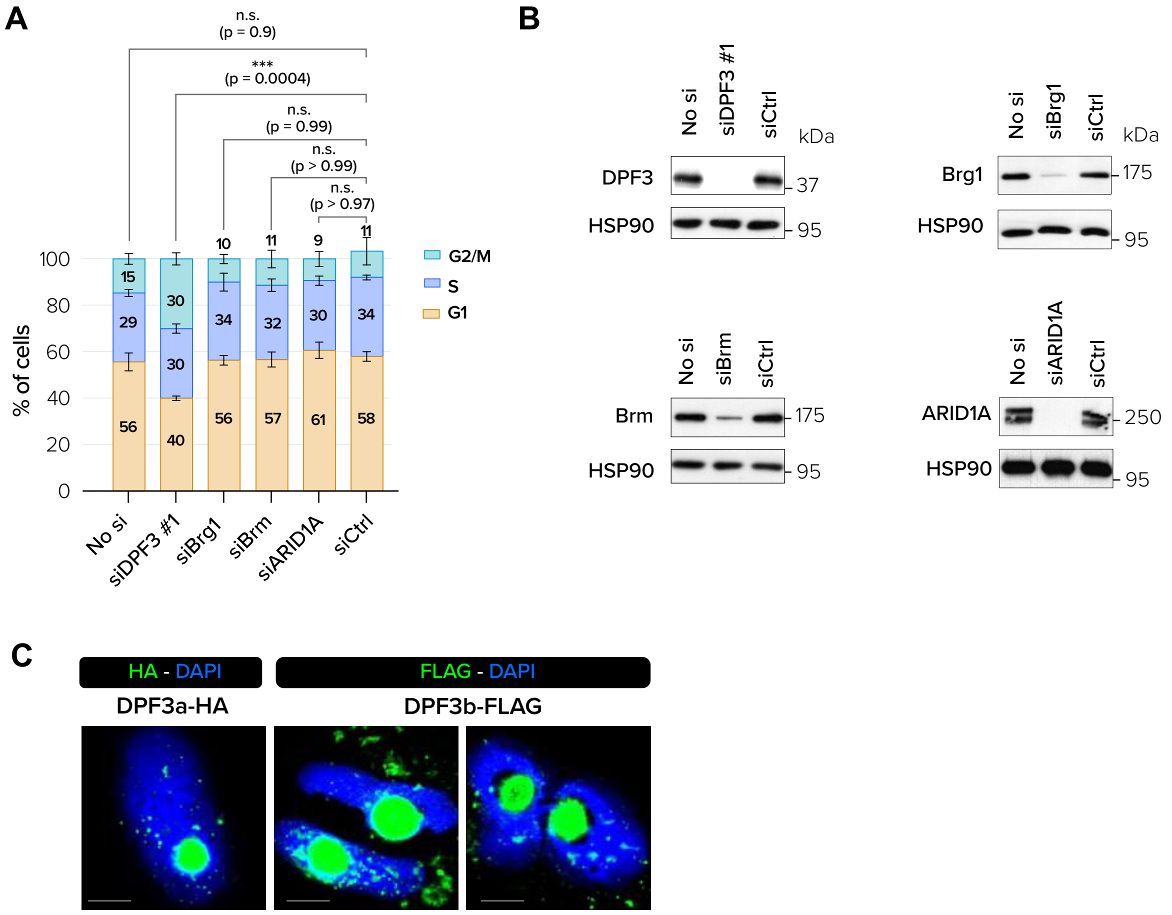

Fig. S4. Depletion of DPF3 but not the core subunits of the BAF complex blocks cells in G2/M (**A**) Cell cycle analysis of U2OS cells transfected with control siRNA (siCtrl), DPF3 siRNA (siDPF3 #1), Brm siRNA, Brg1 siRNA, or ARID1A siRNA. Graph shows means +/- S.D. from 3 independent experiments. P-values were calculated using one-way ANOVA with Tukey post-hoc test. (**B**) In parallel, protein from U2OS cells transfected as in A were extracted and analyzed by western blotting for the indicated proteins. (**C**) U2OS cells were transfected with DPF3-HA or DPF3B-FLAG plasmids for 24 hours and processed for immunofluorescence staining using HA or FLAG antibodies (in green). Merged channels with DAPI nuclear staining (in blue) are shown. Scale bar = 5 µm

Journal of Cell Science • Supplementary information

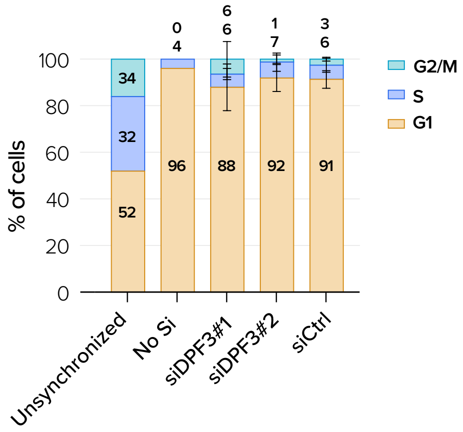

Fig. S5. Serum starvation blocks DPF3-depleted hTEÒRT-RPE-1 cells in G0/G1

Cell cycle analysis of asynchronous hTERT-RPE-1 cells or hTERT-RPE-1 cells mock-transfected (No si) or transfected with one of two different DPF3 siRNAs (siDPF3 #1 and siDPF3 #2) or control siRNA (siCtrl) and serum starved for 48 hours. Results are representative of one experiment performed twice.

Blots in Fig. 1

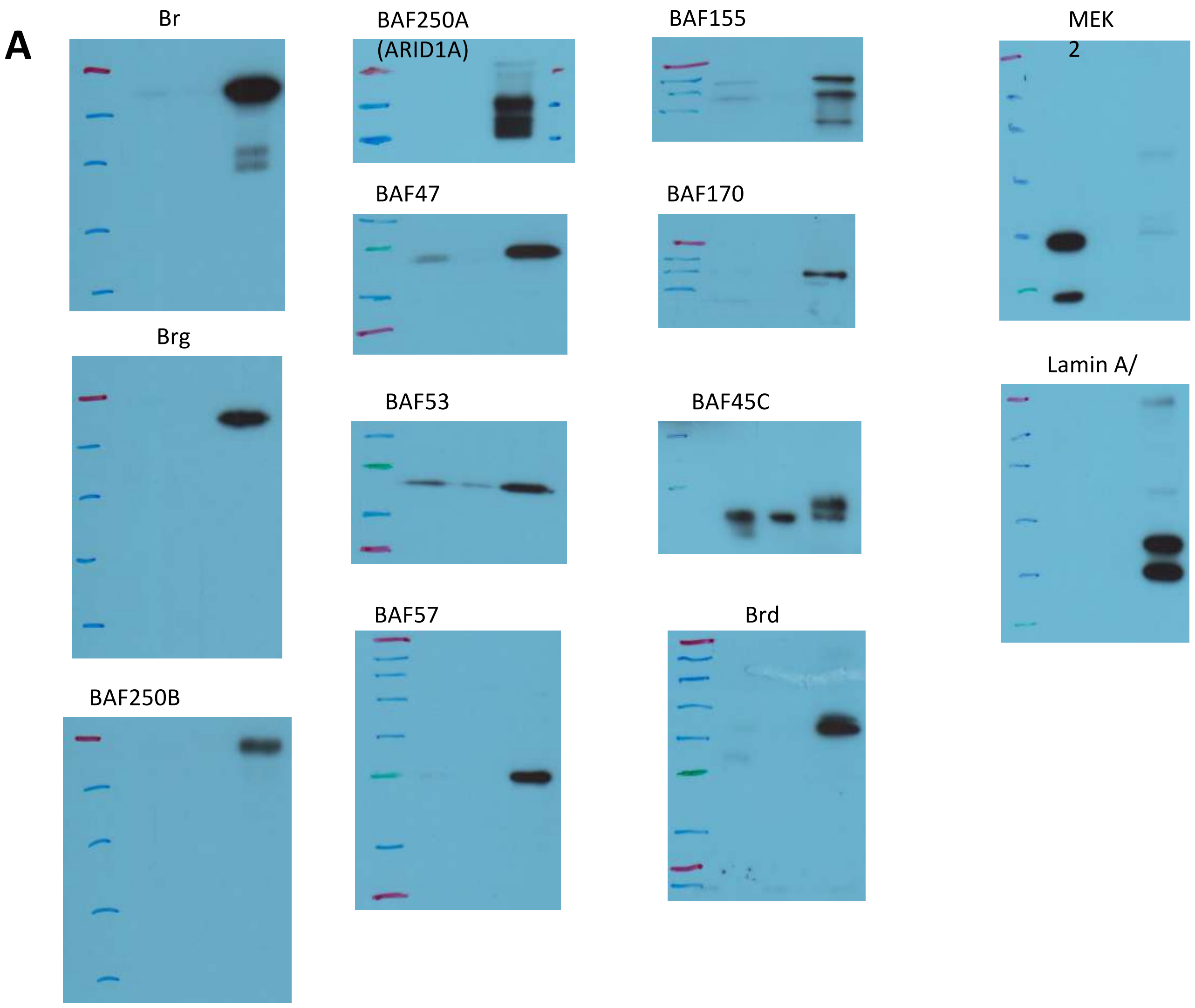

Journal of Cell Science • Supplementary information

Blots in Fig. 1

B

BRG1 (IP BRG1)

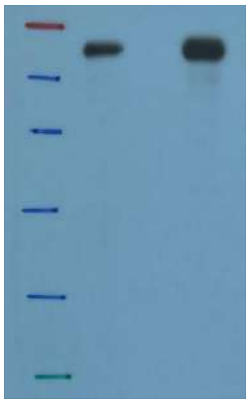

BAF170 (IP BRG1)

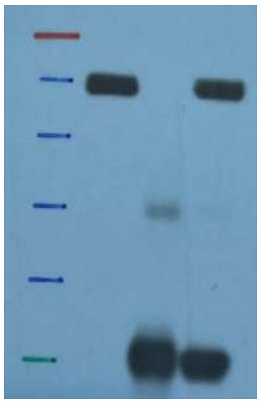

DPF3 (IP BRG1)

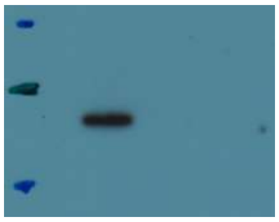

C

BRM (IP BRM)

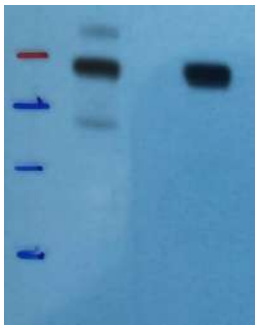

BAF 170 (IP BRM)

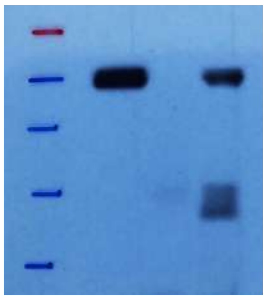

H

BRM(IP DPF3)

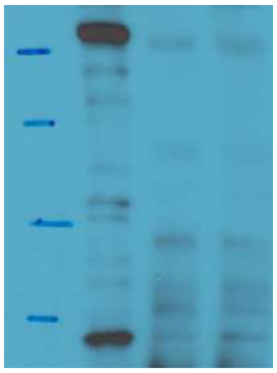

PCM1(IP DPF3)

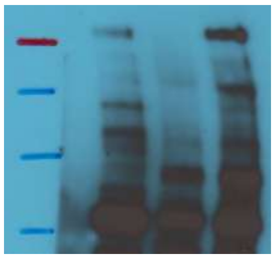

DPF3 (IP DPF3)

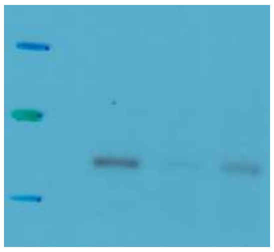

Journal of Cell Science • Supplementary information

Blots in Fig. 3

B

PLK1 (FLAG)

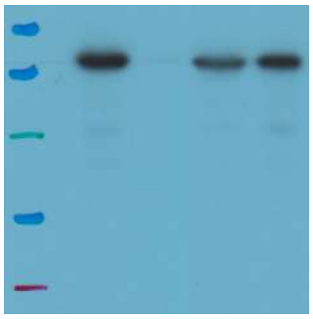

Aurora A (HA)

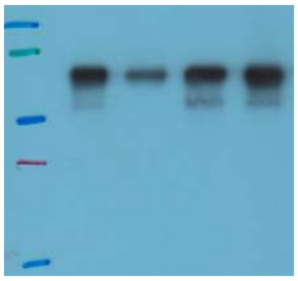

KIF2B (Myc)

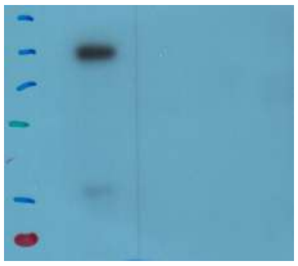

α-Tubulin

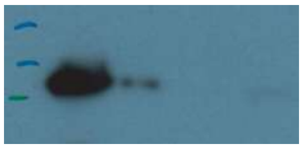

Journal of Cell Science • Supplementary information

Blots in Fig. 4

B

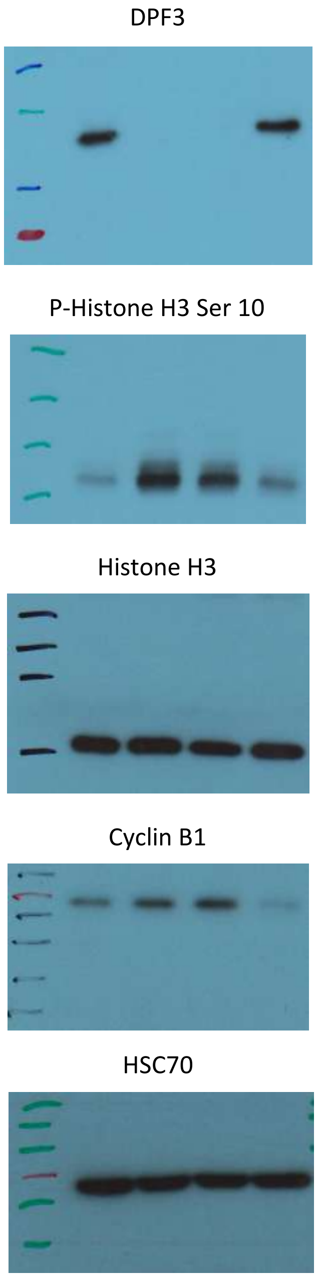

F

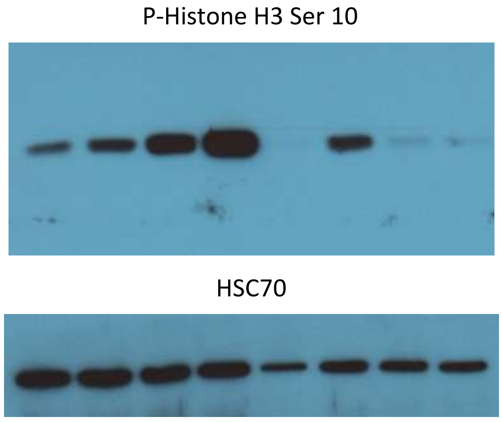

Journal of Cell Science • Supplementary information

Blots in Fig. 6

C

DPF3

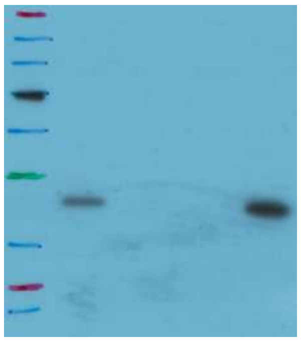

Alpha-Tubulin

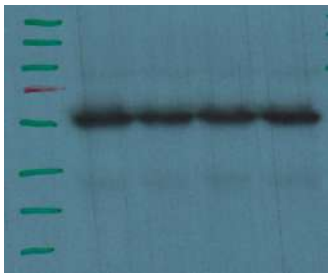

HSC70

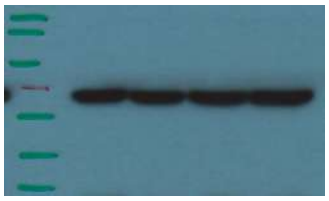

Journal of Cell Science • Supplementary information

Blots in Fig. 7

G

DPF3

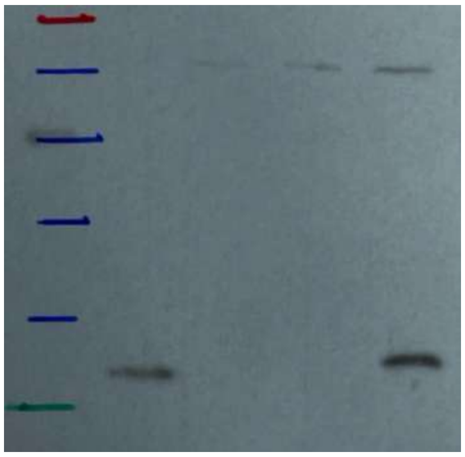

PCM1

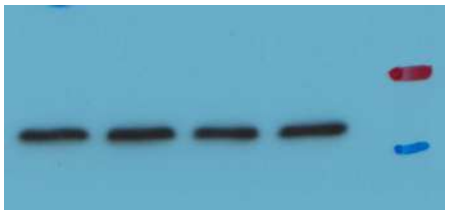

HSC70

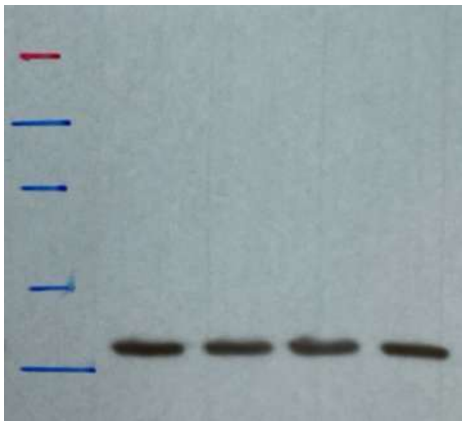

Journal of Cell Science • Supplementary information

Blots in Fig. S1

C

DPF3

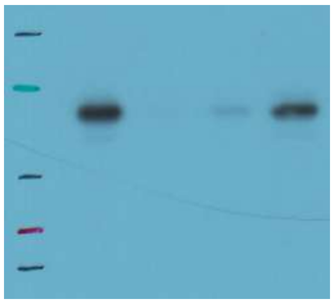

HSC70

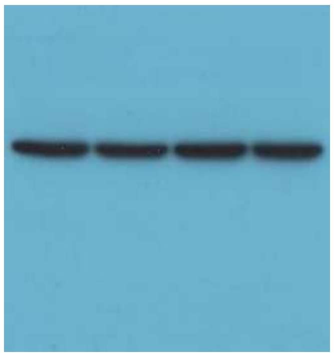

Journal of Cell Science • Supplementary information

Blots in Fig. S4

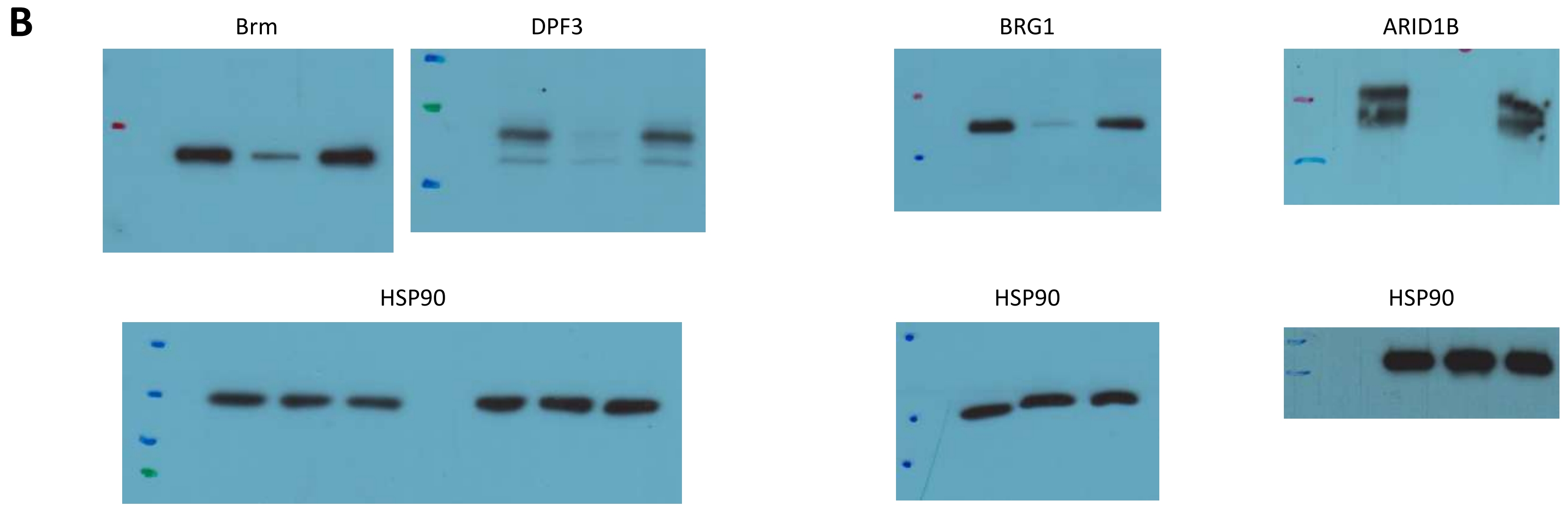

Fig. S6. Blot Transparency

Journal of Cell Science • Supplementary information

Table S1. siRNA sequences

siRNA	Sense Sequence 5' → 3'	Anti-sense Sequence 3' → 5'
ARID1A	GCCCUGAACAAUAACCUCA	UGAGGUUAUUGUUCAGGGC
Brm	GUCCUGGACCUCCAAGUGUCU	AGACACUUGGAGGUCCAGGAC
Brg1	Santa Cruz Biotechnology (#sc-29827)	
DPF3 #1	CCCAGAACAACUGCUACAUTT	AUGUAGCAGUUGUUCUGGGTT
DPF3 #2	GGAGGAAAGCAUCCAGGAATT	UUCCUGGAUGCUUUCCUCCTT
Gl3 (siCtrl)	CUUACGCUGAGUACUUCGAU	AAUCGAAGUACUCAGCGUAAU

Table S2. Sequences of primers used in qPCR experiments

mRNA	Reverse sequence 5' → 3'	Forward Sequence 5' → 3'
DPF3a/b	TTCCTGGATGCTTTCCTCCT	GGCTGCTGGAGATAAAACCTGA
β-actin	AGAGGCGTACAGGGATAGCA	AGAAAATCTGGCACCACACC

Table S3. Antibodies used in western blotting experiments

Antibody	Company	Catalogue number	RRID	Working dilution
α-tubulin (11H10)	Cell Signaling	#2125	AB_2619646	1:5000
β-actin	Santa Cruz	#sc-69879	AB_2714189	1:5000
ARID1A	Abcam	#Ab182560		1:1000
ARID1B	Novus a bio-techne brand	#NBP1-89358	AB_11032492	1:1000
BAF47	Cell Signaling	#91735	AB_2800172	1:1000
BAF53	Cell Signaling	#43910	AB_2799251	1:1000
BAF57	Bethyl	#A300-810A-T	AB_2779472	1:1000
BAF155	Cell Signaling	#11956	AB_2797776	1:1000
BAF170	Cell Signaling	#12760	AB_2798017	1:1000
Brd9	Cell Signaling	#71232	AB_2799798	1:1000
Brg1	Cell Signaling	#3508	AB_2193944	1:1000

Journal of Cell Science • Supplementary information

Brg1 (E8V5B)	Cell Signaling	#72182	AB_2799815	1:1000
Brm	Novus a bio-techne brand	#NBP1-90015	AB_11031434	1:1000
Brm	BD transduction laboratories	#610389	AB_397772	1:2000
Cylin B1	Santa Cruz Biotechnology	#sc-752	AB_2072134	1:1000
DPF3 (E7F7N)	Cell Signaling	#82788		1:500
FLAG	Sigma	#F1804	AB_262044	1:2000
HA (C29F4)	Cell Signaling	#3724	AB_1549585	1:1000
Histone H3	Cell Signaling	#9715	AB_331563	1:2000
Phospho-H3 (Ser10)	Cell Signaling	#3377	AB_1549592	1:2000
HSC70	Santa Cruz	#sc-7298	AB_627761	1:5000
Lamin A/C	Millipore	#MAB3211	AB_94752	1:1000
Mek2	Santa Cruz	#sc-13159	AB_627923	1:1000
c-Myc(Y69)	Abcam	#ab32072	AB_731658	1:1000
PCM1	Cell Signaling	#5213	AB_10556960	1:1000
Secondary antibodies conjugated with horseradish peroxidase (HRP)	Anti-*Rabbit HRP* Antibody<br>Anti-Mouse HRP Antibody<br>Rabbit anti-goat IgG HRP-linked	Cell Signaling *#7074*<br>Dako #P0260<br>Life Technologies #31402	AB_2099233<br>AB_2636929<br>AB_228395	All used 1:5000 to 1:10,000

Table S4. Antibodies used in immunoprecipitation experiments

Antibody	Company	Catalogue number	RRID	Working dilution
Brm (D9E8B)	Cell Signaling	#11966	AB_2797783	2-4 μg/ IP
Brg1 (D1Q7F)	Cell Signaling	#49360	AB_2728743	2-4 μg/ IP
DPF3 (E7F7N)	Cell Signaling	#82788		2 μg/ IP

Journal of Cell Science • Supplementary information

Table S5. Antibodies used in immunofluorescence experiments

Antibody	Company	Catalogue number	RRID	Working dilution
α-tubulin (11H10)	Cell Signaling	#2125	AB_2619646	1:500
α-tubulin (DM1A)	Cell Signaling	#3873	AB_1904178	1:500
Ɣ-tubulin	Santa Cruz	#sc-51715	AB_630410	1:500
Ɣ-tubulin	Genetex	#GTX113286	AB_1952442	1:500
Acetylated α-tubulin	Santa Cruz	#sc-23950	AB_628409	1:500
Aurora B	Abcam	#ab3609	AB_449204	1:100
CENP-A	Invitrogen	#MA1-20832	AB_2078763	1:500
CENP-E	Active Motif	# 39619	AB_2793278	1:200
Centrin-2	Millipore	#04-1624	AB_10563501	1:100
CREST	Immunovision	#HCT-0100	*AB_2744669*	1:1000
CT-K	BD transduction laboratories	#61376		1:500
DPF3	Biorbyt	#Orb182556		1:200
FLAG	Sigma	#F1804	AB_262044	1:500
HA	Cell Signaling	#3724	AB_1549585	1:500
IFT88	Proteintech	#13967-1-AP	AB_2121979	1:500
KIF4A	Novus a bio-techne brand	#NBP2-56589		1:500
MKPL2/KIF20A	Abnova	#H00010112-B01	AB_1146023	1:500
PCM1	Santa Cruz Biotechnology	#sc-398365	AB_2827155	1:500
PRC1	Invitrogen	# **MA1-846**	AB_1086772	1:200
Secondary antibodies	Thermo Fisher Scientific	Goat anti Rabbit IgG Alexa Fluor 488 conjugate #A-	AB_2576217	

conjugated with Alexa dyes		11034<br>Donkey anti-rabbit IgG, Alexa Fluor 488 conjugate #A-21206<br>Goat anti-Mouse IgG, Alexa Fluor 488 conjugate #A-11001<br>Donkey anti-Mouse IgG, Alexa Fluor 546 conjugate #A-10036<br>Goat anti-rabbit IgG, Alexa Fluor 546 conjugate #A-11035	AB_2535792<br>AB_2534069<br>AB_2534012<br>AB_143051	All used 1:2000

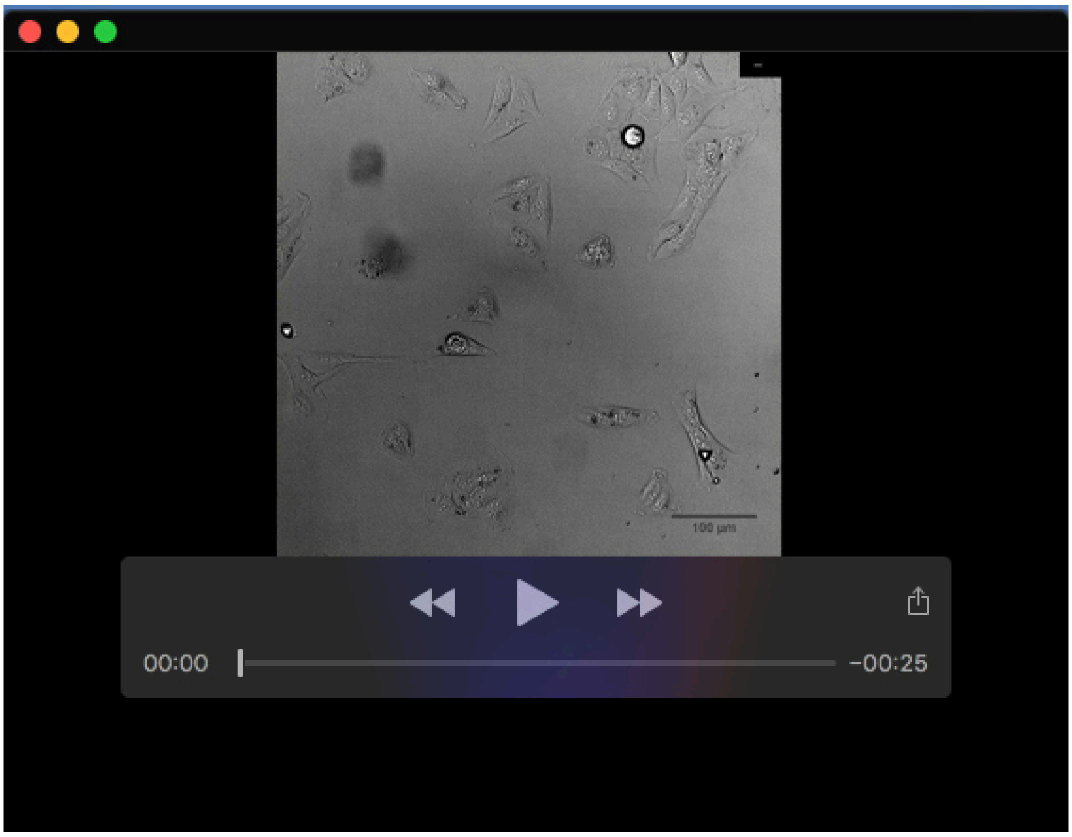

Movie 1. Live-cell imaging of U2OS cells transfected with control siRNA (siCtrl). Cells were transfected for 16 hours using calcium phosphate precipitation method and recovered for 8 hours. Live imaging was then started. Images were acquired every 10 min for a total of 24 hours.

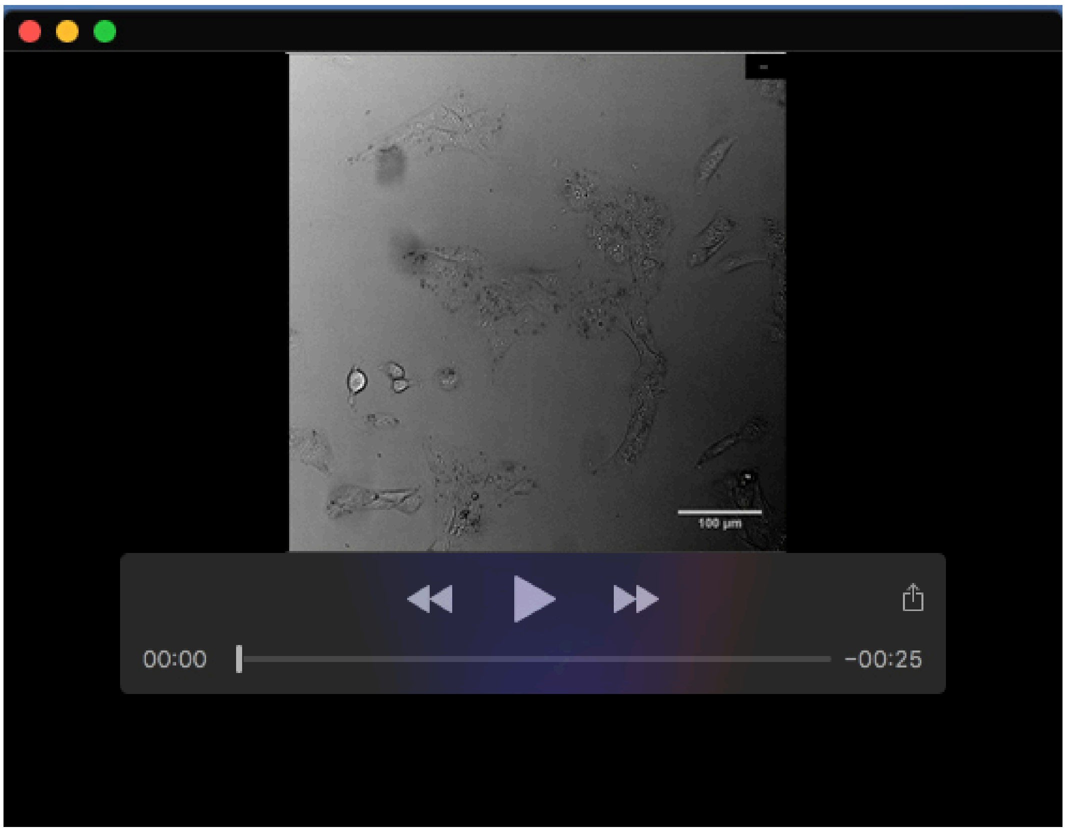

Movie 2. Live-cell imaging of U2OS cells transfected with DPF3 siRNA (si DPF3#1). Cells were transfected for 16 hours using calcium phosphate precipitation method and recovered for 8 hours. Live imaging was then started. Images were acquired every 10 min for a total of 24 hours. DPF3-depleted cells spent an unusually long time in mitosis. They did undergo mitosis and showed induction of post-mitotic apoptotic cell death.

Journal of Cell Science • Supplementary information

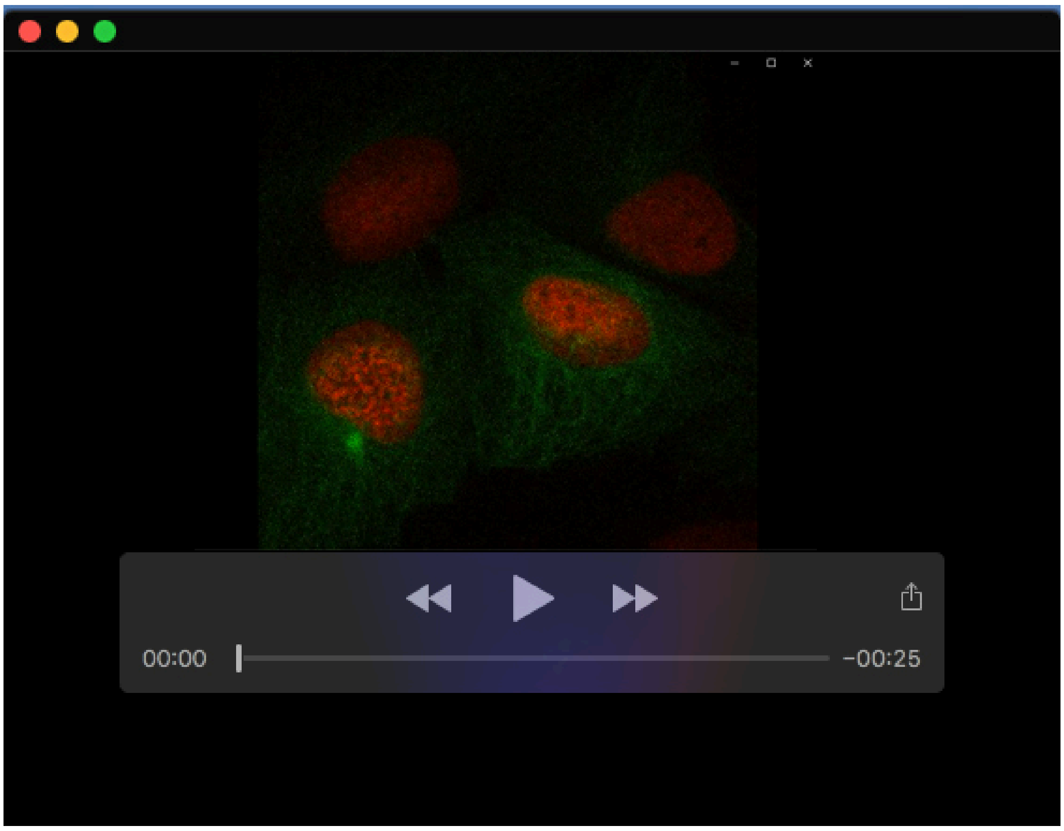

Movie 3. Fluorescent live-cell imaging of H2B-RFP/α-actin-GFP U2OS cells transfected with control siRNA (siCtrl).

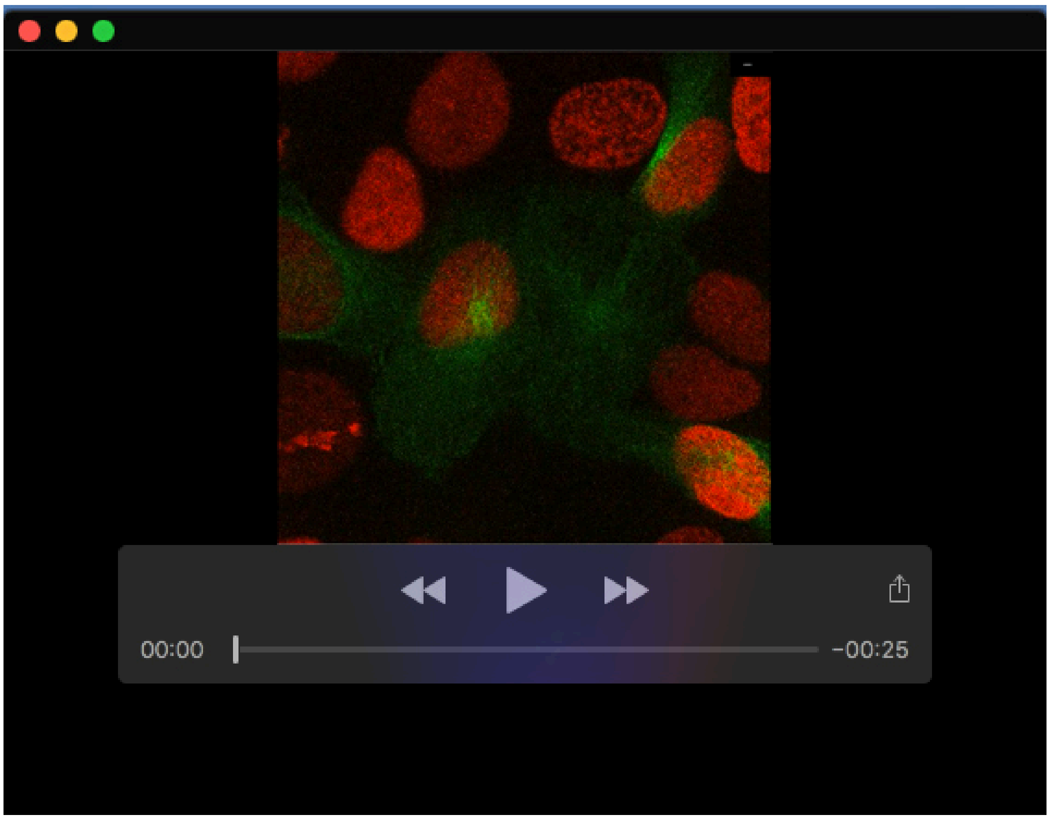

Movie 4. Fluorescent live-cell imaging of H2B-RFP/α-actin-GFP U2OS cells transfected with DPF3 siRNA (si DPF3#1). DPF3-depleted cells exhibited chromosome hyper-oscillations and spent an unusually long time in mitosis.

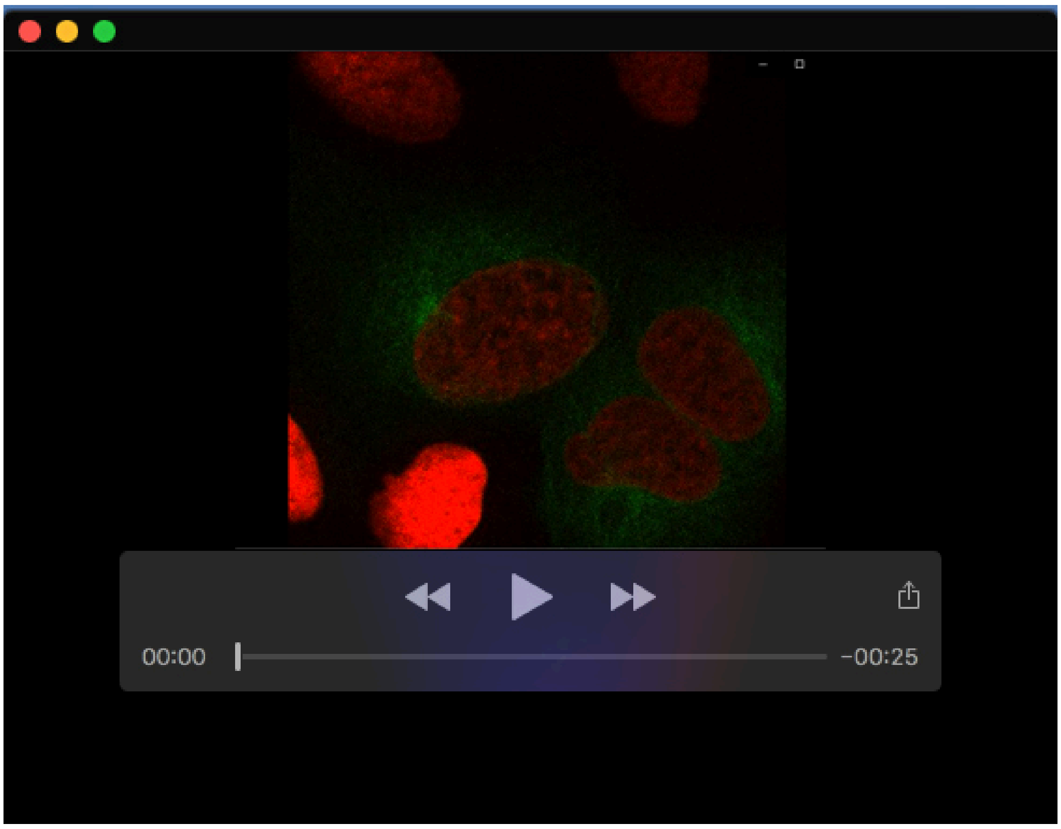

Movie 5. Fluorescent live-cell imaging of H2B-RFP/α-actin-GFP U2OS cells transfected with DPF3 siRNA (si DPF3#1). DPF3-depleted cells exhibited chromosome hyper-oscillations and spent an unusually long time in mitosis.

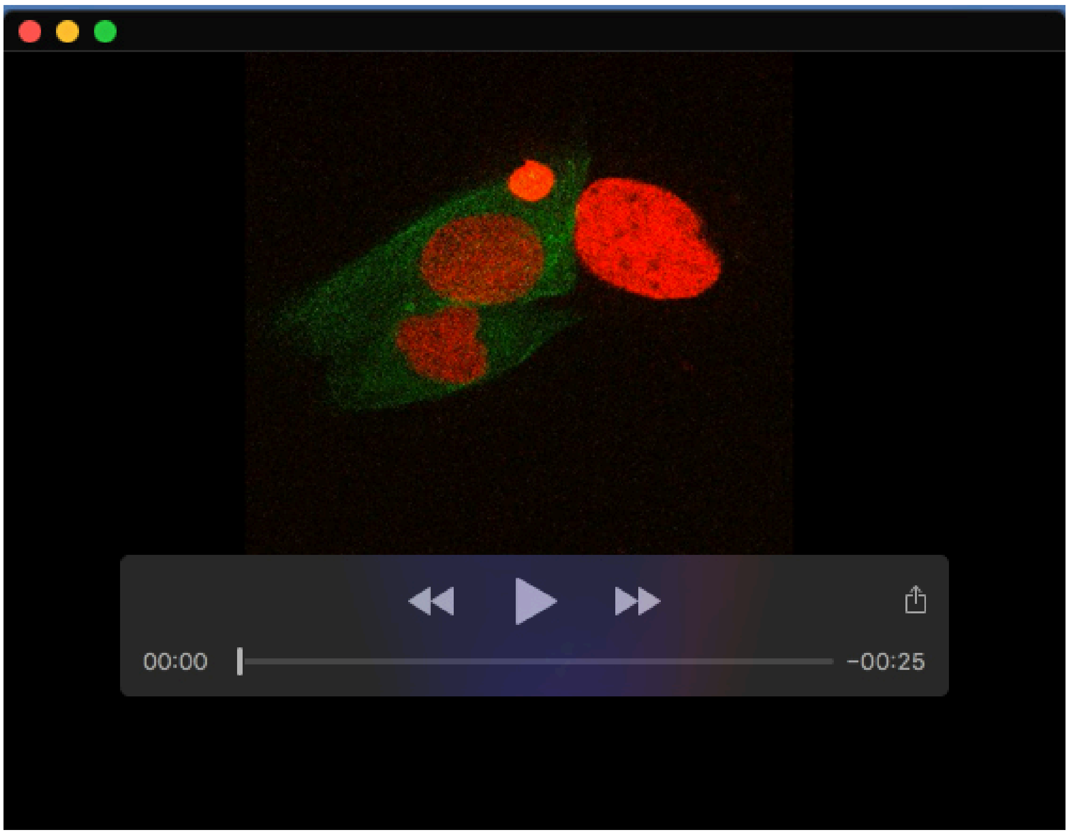

Movie 6. Fluorescent live-cell imaging of H2B-RFP/α-actin-GFP U2OS cells transfected with DPF3 siRNA (si DPF3#1). DPF3-depleted cells did undergo anaphase and subsequent cytokinesis, leading to asynchronous/unscheduled chromatid separation.

Journal of Cell Science • Supplementary information

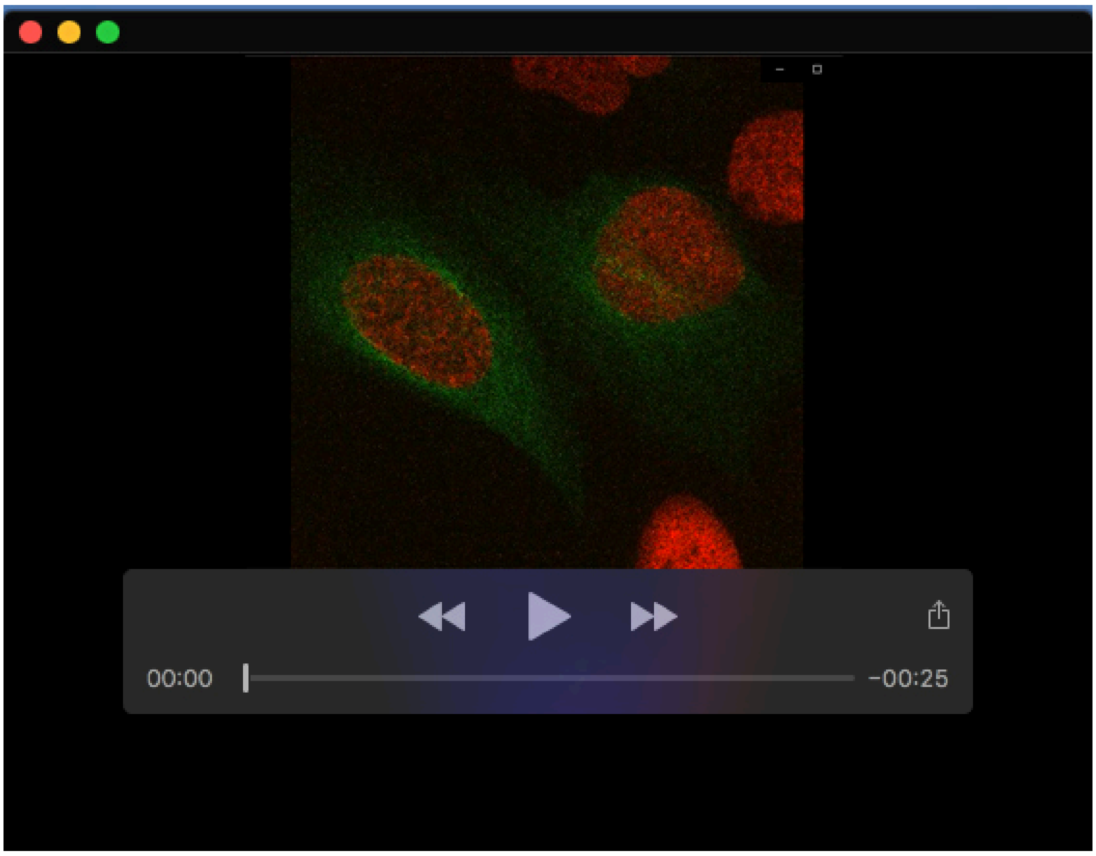

Movie 7. Fluorescent live-cell imaging of H2B-RFP/α-actin-GFP U2OS cells transfected with DPF3 siRNA (si DPF3#1). DPF3-depleted cells did undergo anaphase and subsequent cytokinesis, leading to formation of nuclei with irregular shapes (formation of bi/multi-lobed nuclei).

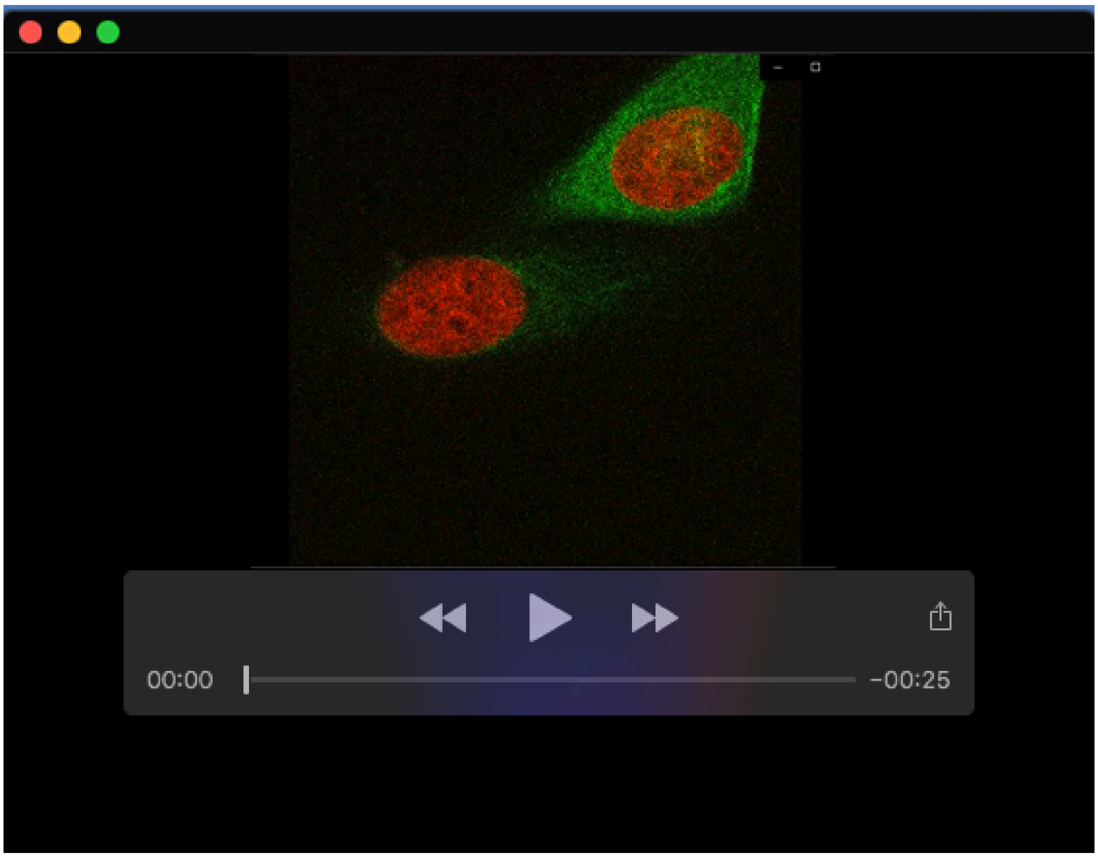

Movie 8. Fluorescent live-cell imaging of H2B-RFP/α-actin-GFP U2OS cells transfected with DPF3 siRNA (si DPF3#1). DPF3-depleted cells showed induction of post-mitotic apoptotic cell death.
