# Supplementary material for: Non-canonical role for the BAF complex subunit DPF3 in mitosis and ciliogenesis
Source: J Cell Sci. 2024 May 13;137(9):jcs261744. doi: 10.1242/jcs.261744 (PMC11166463; doi:10.1242/jcs.261744)
Supplement: Supplementary file 1 [file joces-137-261744_review_history.pdf]

Non-canonical role for the BAF complex subunit DPF3 in mitosis and ciliogenesis

Giulia Verrillo, Anna Maria Obeid, Alexia Genco, Jacopo Scrofani, Francois Orange, Sarah Hanache, Julien Mignon, Tanguy Leyder, Catherine Michaux, Celine Kempeneers, Noemie Bricmont, Stephanie Herkenne, Isabelle Vernos, Maud Martin and Denis Mottet
DOI: 10.1242/jcs.261744

Editor: Renata Basto

Review timeline

Original submission:	27 October 2023
Editorial decision:	24 November 2023
First revision received:	20 February 2024
Editorial decision:	21 March 2024
Second revision received:	2 April 2024
Accepted:	4 April 2024

Original submission

First decision letter

MS ID#: JOCES/2023/261744

MS TITLE: Non-canonical role for the BAF complex subunit DPF3 in mitosis and ciliogenesis

AUTHORS: Giulia Verrillo, Alexia Genco, Anna Maria Obeid, Jacopo Scrofani, Francois Orange, Sarah Hanache, Julien Mignon, Tanguy Leyder, Catherine Michaux, Celine Kempeneers, Noemie Bricmont, Isabelle Vernos, Maud Martin, and Denis Mottet
ARTICLE TYPE: Research Article

We have now reached a decision on the above manuscript.

To see the reviewers' reports and a copy of this decision letter, please go to: https://submit-jcs.biologists.org and click on the 'Manuscripts with Decisions' queue in the Author Area. (Corresponding author only has access to reviews.)

As you will see, the reviewers were interested in your manuscript and the novel findings raised by this study. They raise a number of substantial criticisms that prevent me from accepting the paper at this stage. They suggest, however, that a revised version might prove acceptable, if you can address their concerns. If you think that you can deal satisfactorily with the criticisms on revision, I would be pleased to see a revised manuscript. We would then return it to the reviewers.

Please ensure that you clearly highlight all changes made in the revised manuscript. Please avoid using 'Tracked changes' in Word files as these are lost in PDF conversion.

I should be grateful if you would also provide a point-by-point response detailing how you have dealt with the points raised by the reviewers in the 'Response to Reviewers' box. Please attend to all of the reviewers' comments. If you do not agree with any of their criticisms or suggestions please explain clearly why this is so.

© 2024. Published by The Company of Biologists under the terms of the Creative Commons Attribution License (https://creativecommons.org/licenses/by/4.0/).

Reviewer 1

Advance summary and potential significance to field

Verrillo, Mottet and colleagues here explore potential roles for the BAF complex component DPF3 in centrosome and ciliary dynamics, as well as in mitosis. They present centrosome- and kinetochore-proximal localisations for DPF3 and show that its depletion causes significant problems during mitosis, along with impaired primary ciliogenesis. The data provide strong evidence that DPF3 has functions in allowing ciliogenesis and successful mitosis, as per the title, but where these functions arise is not entirely clarified here. The mitotic phenotypes are well described, with a great deal of detail, but the localisation experiments are not entirely convincing, so that the functional or mechanistic conclusions are limited.

Comments for the author

There are some important aspects of the study that should be revised in order to provide a more definitive set of findings about DPF3's roles.

1. The mitotic phenotypes can potentially be explained by different kinetochore-related activities of DPF3, which are not entirely clear from the findings presented here. The current dataset is not sufficient to draw conclusions regarding the localisation or function of DPF3 on (mitotic) chromosomes. The CENP-A localisation is not well resolved in that the paired centromeres are not distinct and it is unclear whether all CENP-A signals contain DPF3. The CREST and PLK1 stainings are also somewhat limited. These issues question the authors' conclusions regarding DPF3's chromosomal localisation and a more complete analysis should be provided to define this. Mitotic chromosomes should be examined with additional markers to determine whether DPF3 is localising to the centromere, the kinetochore, the inner centromere, or with the chromosomal passengers. Some additional biochemical data would also be useful to consider the kinetochore roles of DPF3.

2. Line 169: PCM1 is mainly considered a centriolar satellite protein (see, e.g. Hall et al. PMID: 36790165), rather than a marker for the pericentriolar material (despite the name). The data should be reinterpreted or the interpretation rephrased with this point in mind, as the localisation of DPF3 seems to be more clearly a satellite localisation than a pericentriolar material protein. The loss of DPF3 signal in mitosis is very supportive of such a localisation. This seems to be the authors' principal conclusion as well, so the manuscript might be reviewed to make this clearer.

3. Line 194: In a related point, centrosome purification will generally separate centrioles and pericentriolar material away from the satellites, although residue will remain. As the pericentriolar material has a specific arrangement that can be visualised with antibodies to pericentrin (for example), it would be appropriate to determine whether DPF3 is a pericentriolar matrix protein or a centriolar satellite component by other analyses.

4. It should be tested in the various human cancer cell lines examined (HeLa, MDA-MB 231, T47D and MCF7) whether DPF3 colocalises with satellites.

5. A rescue control must be provided for the siRNA experiments; this need not duplicate all the mitotic analyses, but it should be demonstrated that the main intra-M delay and the ciliogenesis can be rescued.

6. Line 165: Gamma-tubulin is not solely localised to centrioles; this is not an ideal centriolar marker and the authors should use an additional label to test the specific localisation at centrioles.

7. The impact of DPF3 depletion on PCM1 localisation is potentially very significant in the context of the ciliary phenotype and the presumptive satellite functions of DPF3. However, it is not clear how the dispersion of the PCM1 was determined (as there is no marker specified for where the dispersion arises, if the satellites are disrupted). This should be clarified.

Minor points

© 2024. Published by The Company of Biologists under the terms of the Creative Commons Attribution License (https://creativecommons.org/licenses/by/4.0/).

8. Line 114: The centrosome is distinct from the spindle pole body; the terms should not be separated by a slash. As the authors do not examine DPF3 localisation in yeast cells, the inclusion of the spindle pole body here is not accurate and should be removed.

9. Line 145: This strong conclusion is not supported by the absence of the DPF3 from the Brg1/BRM immunoprecipitates and should be rephrased.

10. The resolution of the blow-up images should be improved, so that single-channel images are shown blown up. The merged image can be at large scale, so that the general cell image is visible, but the single-channel images are key for the understanding of the localisation and the relevant structures are not very visible in the larger images. Individual cells in each large-scale image would be sufficient. This is relevant to Figs. 1F, 1I, 2A-C, S1D-G.

11. It is desirable for all micrographs to be presented at the same scale- this does not seem to be the case in the blow-ups for Figs. S1D and 1G, for example.

12. Figure 6H should show clearer blow-up detail. With the very broad localisation of DPF3 in these cells, it is not convincing that there is a localisation at the ciliary base.

13. Abbreviations should be provided for non-standard labels within Figures (e.g., the legend to Fig 1A).

<u>Reviewer 2</u>

Advance summary and potential significance to field

In this study, Verillo et al., describe the unexpected function of the DPF3 component of the chromatin remodeling complex BAF in mitosis and ciliogenesis.

They show in cell culture that DPF3 is localized at different loci during mitosis and interphase in addition to the nucleus. They show that DPF3 localizes to the centrosome/spindle pole body, the spindle midzone/bridging fiber area, and the midbody during mitosis. In quiescent cells, DPF3 also localizes to the basal body and associates with centriolar satellites. Using siRNA inactivation, they show that DPF3 is required for mitotic progression, chromosome alignment/distribution during mitosis, and cilium assembly in postmitotic cells. Taken together, the data are compelling and reveal a novel role for this protein that is unlikely to be related to its role in chromatin remodeling. Thus, this work adds a novel function to the chromatin remodeling class of proteins and should have a wide audience in the field of cell science.

Comments for the author

I have just a few points that need to be addressed before publication:

-The authors state that DPF3 is involved in the "regulation of dynamic PCM1 distribution". This conclusion is based on IF observations (Figure 6D). The staining does not show the centrioles, so it is difficult to really determine how the satellites are distributed after DPF3 depletion. Could the authors quantify PCM1 around centrioles in control and DPF3 depleted cells? Could the authors also show that the overall expression of PCM1 is not affected, which would better support a role in satellite distribution or assembly?

-The authors clearly show that the axoneme does not grow in DPF3-depleted cells and conclude that "the cilary vesicles dock to the plasma membrane as judged by the appearance of appendages". This sentence is unclear to me because distal appendages are formed before docking. In the EM images, only very small CVs are seen in DPF3-depleted cells, and none are actually docked to the TF. The CV does not seem to form properly, suggesting a very early defect in centriole to basal body conversion. The sections do not really allow to show that the centriole is normal as stated by the authors. Therefore, the conclusions must be qualified.

Minor points:

© 2024. Published by The Company of Biologists under the terms of the Creative Commons Attribution License (https://creativecommons.org/licenses/by/4.0/).

Apparently a similar role of chromatin remodeling complexes at the mitotic apparatus has been recently described in addition to other cited references (Messina et al. BMC Biology (2022) 20:172 https://doi.org/10.1186/s12915-022-01365-5). The authors should discuss their results also in light of this study, in particular for researchers outside the chromatin field.

Figure 5A: the different classes of defects are not easy to see, for example it is not clear to me how the authors determine kinetochores with lateral attachment. A zoom on a control kinetochore/KF attachment could be useful.

Line 261: the levels of pH (Ser10) already decreases 12h post release.

Line 270: “altogether these data demonstrate that DPF3 ……, therefore maintaining genomic stability” this last part of the sentence is overstated here. This is only suggested in the next section.

Figure 2A: I do not see white arrows or arrowheads on the figure

Figure 6F (Change FT to TF).

Reviewer 3

Advance summary and potential significance to field

The manuscript entitled “non-canonical role for the BAF complex subunit DPF3 in mitosis and ciliogenesis” by Verrillo et al. demonstrates new roles for the BAF chromatin remodelling complex subunit DPF3 in ciliogenesis and kinetochore-microtubule attachments, which have never been described before.

Comments for the author

The data provided are of good quality, well designed, explained and support the new roles proposed for the DPF3 protein. The manuscript is also well written and illustrated. I recommend it for publication when the authors clarify the points listed, provide some supplementary control experiments and correct some mistakes of cell cycle analysis in the manuscript.

Major points:

1. Subcellular localization of DPF3 at the centrosome could be better characterized in combination with the centriole distal marker centrin and bona fide markers of the pericentriolar material cdk5rap2 or pericentrin, as PCM1 is a better marker of satellites. In multiciliated cells, centrin could also be used as an alternative to Plk4 staining.

2. Perturbations of DPF3 function were performed after siRNA-based experiments. Rescue experiments are required to strengthen the specificity of the siRNA-based loss of function.

3. In the whole manuscript, the authors could not discriminate between the two isoforms DPF3a and DPF3b in terms of sub-cellular localization and function because of the tools available. It is an important point to address as the two isoforms might be redundant or alternatively have distinct roles in ciliogenesis and/or kinetochore-microtubule attachments. One possibility would be to perform rescue experiments with the two isoforms separately to clarify this point.

4. The authors cannot discriminate from the data provided in Figure 3 whether cells are blocked in G2 phase of the cell cycle and/or in mitosis. Therefore, they cannot conclude after Figure 3 that “DPF3 is required for mitotic progression and cell survival, therefore maintaining genomic stability” (lines 269-270).
Indeed, it is the phosphorylation of cyclin B1 and not the level of expression per se which can be used as a marker of mitosis (Figure 3B). Cyclin B1 level is increasing from G2 phase onwards in cells. The same is true for histone 3 phosphorylation on Ser10, which starts in G2 phase of the cell cycle and accumulates in mitosis (Figure 3B). FACS analysis (Figure 3C and E) does not enable to

© 2024. Published by The Company of Biologists under the terms of the Creative Commons Attribution License (https://creativecommons.org/licenses/by/4.0/).

discriminate between G2 and mitosis either. Aphidicolin treatment does not block cells in G1/S (line 251), but in S phase and is a source of DNA damage (Figure 3E). The authors should repeat the experiment using cdk4/6 inhibitors to block cells at the G1/S checkpoint without inducing DNA damage.

5. In the movies provided (movies 2 and 3), it seems that in a first-place chromosomes do not fail to align and even form a metaphase plate, but that in a second place only they start oscillating and leaving the metaphase plate. Can it be that it is the maintenance of kinetochore-microtubule attachments which is mainly impaired and not the establishment (as proposed in the main text, lines 276 or 308)?

6. In Figure 5A, it is difficult to appreciate whether cells after no si, siCtrl or siDPF3 are at the same stage of mitosis for analysis. One possibility would be to incubate cells with a MG132 inhibitor to favour the accumulation of cells in metaphase by preventing anaphase onset before the cold-depolymerization assay.

Minor points:
1. The technique used to quantify is not always mentioned in the figure legend, like in Fig3A and 3G
2. Abbreviations are not explained in Fig1A (CF, NSF, CSF)
3. Material and methods section should include the procedures for quantifications (Figures 5B, 5D, 6E)
4. In figure 5A, kinetochore fibre width and density are not clearly visualized after only merged panels. The authors should provide in black and white microtubule staining as well for better visualization. They should also provide examples of normal kinetochore-microtubule attachments for comparison. High magnification panels are not necessarily clearly illustrating the defects spotted.
5. Kinetochore fibre width should also be quantified to better evaluate K fibre stability.
6. The discussion should be shortened and refocused on the potential function of DPF3 at the centrosome, in axoneme growth and in kinetochore-microtubule stability maintenance instead of listing the pathologies related to cilia defects.

First revision

Author response to reviewers' comments

Answers to reviewer’s comments are in blue

Reviewer#1
Verrillo, Mottet and colleagues here explore potential roles for the BAF complex component DPF3 in centrosome and ciliary dynamics, as well as in mitosis. They present centrosome- and kinetochore-proximal localizations for DPF3 and show that its depletion causes significant problems during mitosis, along with impaired primary ciliogenesis. The data provide strong evidence that DPF3 has functions in allowing ciliogenesis and successful mitosis, as per the title, but where these functions arise is not entirely clarified here. The mitotic phenotypes are well described, with a great deal of detail, but the localization experiments are not entirely convincing, so that the functional or mechanistic conclusions are limited. There are some important aspects of the study that should be revised in order to provide a more definitive set of findings about DPF3’s roles.

Answer: We are pleased that the reviewer found an interest in our study. We thank this reviewer for her/his comments and suggestions on localization experiments. We have addressed all of them. We hope that our manuscript is now strengthened and can be accepted for publication in JCS.

Major Comments

Comment#1: The mitotic phenotypes can potentially be explained by different kinetochore-related activities of DPF3, which are not entirely clear from the findings presented here. The current

© 2024. Published by The Company of Biologists under the terms of the Creative Commons Attribution License (https://creativecommons.org/licenses/by/4.0/).

dataset is not sufficient to draw conclusions regarding the localization or function of DPF3 on (mitotic) chromosomes. The CENP-A localization is not well resolved in that the paired centromeres are not distinct and it is unclear whether all CENP-A signals contain DPF3. The CREST and PLK1 stainings are also somewhat limited. These issues question the authors' conclusions regarding DPF3's chromosomal localization and a more complete analysis should be provided to define this. Mitotic chromosomes should be examined with additional markers to determine whether DPF3 is localizing to the centromere, the kinetochore, the inner centromere, or with the chromosomal passengers. Some additional biochemical data would also be useful to consider the kinetochore roles of DPF3.
Answer: As requested by this reviewer, the localization of DPF3 in the multifunctional spindle midzone during anaphase has been further examined using specific markers for different chromosomal compartments including kinetochore-associated protein (CENP-E), chromosomal passenger complex (Aurora B and MKLP2/KIF20A) and bridging fiber area (PRC1). New Figure 3A in the revised form of our manuscript shows that DPF3 co-localizes with kinetochore component as well as with chromosomal passenger complex (CPC) and bridging fiber proteins in anaphase cells. The conclusion and discussion section has been modified according to these new data. Moreover, additional biochemical experiments have been done to further characterize the kinetochore roles of DPF3 as requested (see Comment #5 below, last paragraph). We hope that our conclusion regarding the chromosomal localization of DPF3 during mitosis is now more complete.

Comment#2: Line 169: PCM1 is mainly considered a centriolar satellite protein (see, e.g. Hall et al. PMID: 36790165), rather than a marker for the pericentriolar material (despite the name). The data should be reinterpreted or the interpretation rephrased with this point in mind, as the localization of DPF3 seems to be more clearly a satellite localization than a pericentriolar material protein. The loss of DPF3 signal in mitosis is very supportive of such a localization. This seems to be the authors' principal conclusion as well, so the manuscript might be reviewed to make this clearer.
Answer: This reviewer is right. We agree that PCM1 is centriolar satellite marker. We apologize for this mistake. We have reviewed the manuscript according to this pertinent comment. Moreover, we have performed additional co-staining between DPF3 and Centrin (centriole marker) and γ-tubulin (pericentriolar matrix marker). High- resolution microscopy analysis (Figure 1G in the revised version of our manuscript) shows that DPF3 did not co-localize with centrin or γ-tubulin; suggesting that DPF3 can be strictly considered as a centriolar satellite protein.

Comment#3: Line 194: In a related point, centrosome purification will generally separate centrioles and pericentriolar material away from the satellites, although residue will remain. As the pericentriolar material has a specific arrangement that can be visualized with antibodies to pericentrin (for example), it would be appropriate to determine whether DPF3 is a pericentriolar matrix protein or a centriolar satellite component by other analyses.
Answer: We have performed additional immunofluorescence experiments followed by high-resolution microscopy analysis to better determine whether DPF3 is also present in the pericentriolar matrix and/or centrioles. As shown in new Figure 1G of the revised version of our manuscript, we did not observe co-staining between DPF3 and centrin (marker of centriole) and γ-tubulin (marker of pericentriolar matrix). In contrast a perfect co-localization with PCM1 was observed. These data suggest that DPF3 can be strictly considered as a centriolar satellite protein.

Comment#4: It should be tested in the various human cancer cell lines examined (HeLa, MDA-MB 231, T47D and MCF7) whether DPF3 co-localizes with satellites.
Answer: Confocal microscopy shows that DPF3 seems to co-localize with γ-tubulin in different cell types (Figure S1G). This data suggests that DPF3 is similarly co-localized with centrosome compartment in various cell lines. To further determine whether DPF3 co-localizes with centriolar satellites, pericentriolar matrix and centrioles in those cell types, we used U2OS and HeLa cells as representative cellular models. Immunofluorescence staining followed by high-resolution microscopy analysis shows that DPF3 perfectly co-localizes with PCM1. In contrast neither centrin nor γ-tubulin co- localizes with DPF3 (New Figures 1G and S2 in the revised version of our manuscript), suggesting that the centriolar satellite localization of DPF3 is not cell type specific.

Comment#5: A rescue control must be provided for the siRNA experiments; this need not duplicate all the mitotic analyses, but it should be demonstrated that the main intra- M delay and the ciliogenesis can be rescued.

© 2024. Published by The Company of Biologists under the terms of the Creative Commons Attribution License (https://creativecommons.org/licenses/by/4.0/).

Answer: We fully agree that rescue experiments would be useful for confirming siRNA specificity but also to decipher which exogenously expressed DPF3 isoforms - DPF3a and/or DPF3b - could rescue the loss-of-function phenotype. However, we are actually facing technical challenge to address this question. Indeed, the overexpressed DPF3a or DPF3b cause formation of protein-like aggregates in eukaryotic cells (see Figures below). Because maintenance of protein solubility is a fundamental aspect of cellular homeostasis, DPF3 aggregation might be associated with undesired/artefactual cell phenotypes and cannot lead us to properly conclude about the effect of DPF3 re- expression.
However, although the overexpression profile of DPF3 seems to be aberrant, a faint staining is observed between DPF3a and γ-tubulin, making us confident about the centrosomal localization of DPF3 in a more appropriated (over)expressing system.

NOTE: We have removed unpublished data that had been provided for the referees in confidence.

To avoid erroneous conclusions when interpreting data from overexpressing experiment, we are actually developing a tetracycline-inducible gene expression system (TET-On system), which will afford tunable level of overexpressed DPF3 protein. Using this system we will re-express the siRNA-resistant wild type forms of DPF3 or DPF3 cDNA deleted for different domains. This will help us not only to determine the importance of each isoform but also to identify which functional domains(s) of DPF3a/b is (are) necessary for mitotic progression. These cellular models are currently under development and they will be used in a future project. Although rescue experiments would have been informative, we do believe that, at this stage, those rescue experiments are not highly mandatory for the first demonstration of a role for DPF3 in mitosis and ciliogenesis.

However, we have been able to address some specific role/difference for DPF3a and DPF3b using purified recombinant proteins. For instance, microtubule co- sedimentation and GST pull-down assays revealed that recombinant DPP3a protein, and to le a lesser extend, recombinant DPF3b protein can bind polymerized microtubules but none of them interacts with monomer α-tubulin. These data have been added in the revised version of our manuscript (Supplemental Figures S3 in the revised version of our manuscript). Moreover, by GST pull-down assay we have also demonstrated that both DPF3a and DPF3b can interact with PLK1 and Aurora B kinases (new Figure 3B in the revised manuscript), which is consistent with the co- localization with CPC complex during anaphase (new Figure 3A in the revised manuscript). Thanks to these new biochemical data, we bring at some points evidences about the differences/similarities for DPF3a and DPF3b.

Comment#6: Line 165: Gamma-tubulin is not solely localized to centrioles; this is not an ideal centriolar marker and the authors should use an additional label to test the specific localization at centrioles.
Answer: As suggested by this reviewer (*see* comment#3), we have performed an additional co-staining between DPF3 and Centrin, which is a more pertinent centriole marker. High-resolution microcopy (Figures 1G and S2 in the revised version of our manuscript) shows that DPF3 did not co-localize with centrin, suggesting that DPF3 is not localized to centrioles.

Comment#7: The impact of DPF3 depletion on PCM1 localization is potentially very significant in the context of the ciliary phenotype and the presumptive satellite functions of DPF3. However, it is not clear how the dispersion of the PCM1 was determined (as there is no marker specified for where the dispersion arises, if the satellites are disrupted). This should be clarified.
Answer: Thank you for this suggestion. The accumulation of PCM1 in centriolar satellites was apparent in control-depleted cells, but it was considerably diminished upon DPF3 depletion. We have now quantified the signal intensity of PCM1 staining around the centrosome in both control and DPF3-depleted cells as previously published (PMID: 31023719).
To quantify PCM1 dispersion, a circle at the basis of cilia was drawn in control condition (No si). In DPF3-depleted cells, the circle was place in the area where the signal is still brightest (*see* figure 7D in the revised manuscript). The pixel density of anti-PCM1 staining in this area was measured using Image J software. Fluorescence intensity was quantified and signal level in control cells (No si) was normalized to 1.
Analysis of control cells and DPF3-depleted cells from three different experiments is presented in new Figure 7F of the revised manuscript. A decrease in integrated anti- PCM1 immunofluorescence intensities confirms PCM1 redistribution in DPF3-depleted cells.
In addition, we have analyzed the expression of PCM1 upon DPF3 depletion by western blot (new

© 2024. Published by The Company of Biologists under the terms of the Creative Commons Attribution License (https://creativecommons.org/licenses/by/4.0/).

Figure 7G in the revised manuscript). PCM1 expression is not affected in DPF3-depleted cells. Together these results suggest that DPF3 is more likely involved in the regulation of centriolar satellite distribution/organization rather the assembly *per se*.

Minor Comments

Comment#8: Line 114: The centrosome is distinct from the spindle pole body; the terms should not be separated by a slash. As the authors do not examine DPF3 localization in yeast cells, the inclusion of the spindle pole body here is not accurate and should be removed.
Answer: Thank you very much for this suggestion. We have removed spindle pole body as requested.

Comment#9: Line 145: This strong conclusion is not supported by the absence of the DPF3 from the Brg1/ BRM immunoprecipitates and should be rephrased.
Answer: We fully agree with this reviewer's comments. We have changed the conclusion of this section in the revised version of our manuscript.

Comment#10: The resolution of the blow-up images should be improved, so that single-channel images are shown blown up. The merged image can be at large scale, so that the general cell image is visible, but the single-channel images are key for the understanding of the localization and the relevant structures are not very visible in the larger images. Individual cells in each large-scale image would be sufficient. This is relevant to Figs. 1F, 1I, 2A-C, S1D-G.
Answer: As requested by this reviewer we have modified the corresponding Figures as following:

- Figure 1F: The entire figure has been removed. New IF experiments followed by high-resolution microscope analysis have been done and are now presented in Figure 1G.
- Figure 1I (now figure 1H in the revised version): We have just kept DPF3/γ-tubulin staining. We have enlarged each single-channel images to better visualize purified centrosomes.
- Figure 2A (now Figure 2 in the revised version): We have enlarged each single- channel images to better visualize the DPF3 localization in relevant mitotic structures.
- Figure 2B: The entire figure has been removed. New IF experiments and high- resolution microscopy analysis have been performed and images are now presented in Figure 3A in the revised version of our manuscript.
- Figure 2B (now Figure 3C in the revised version): We have the pictures.
- Figure S1D: *see* comment#11 below
- Figure S1G: *see* comment#11 below

Comment#11: It is desirable for all micrographs to be presented at the same scale- this does not seem to be the case in the blow-ups for Figs. S1D and 1G, for example.
Answer: We double-checked and images in the supplemental Figures S1D and S1G are presented at the same scale bar. Please note that for HeLa cells a new set of IF data are now presented in Figure S2 according to comment#4.

Comment#12: Figure 6H should show clearer blow-up detail. With the very broad localization of DPF3 in these cells, it is not convincing that there is a localization at the ciliary base.
Answer: Regarding staining in multiciliated cells, we were limited by the number of different primary antibody species to perform triple immunofluorescence staining. We could not stain DPF3 with axoneme marker (acetylated α-tubulin) and centrin due to limited availability of antibodies from different species working in IF.
We are aware that localization of DPF3 in multiciliated cells requires deeper characterization, which might be the scope of another study. We have therefore decided to remove those data and make more space for additional data requested by the three reviewers.

Comment#13: Abbreviations should be provided for non-standard labels within Figures (e.g., the legend to Fig 1A).
Answer: Sorry for this inconvenience. We have carefully checked all abbreviations in the revised version of our manuscript.

Reviewer#2
In this study, Verillo *et al.*, describe the unexpected function of the DPF3 component of the

© 2024. Published by The Company of Biologists under the terms of the Creative Commons Attribution License (https://creativecommons.org/licenses/by/4.0/).

chromatin remodeling complex BAF in mitosis and ciliogenesis. They show in cell culture that DPF3 is localized at different loci during mitosis and interphase in addition to the nucleus. They show that DPF3 localizes to the centrosome/spindle pole body, the spindle midzone/bridging fiber area, and the midbody during mitosis. In quiescent cells, DPF3 also localizes to the basal body and associates with centriolar satellites. Using siRNA inactivation, they show that DPF3 is required for mitotic progression, chromosome alignment/distribution during mitosis, and cilium assembly in post-mitotic cells. Taken together, the data are compelling and reveal a novel role for this protein that is unlikely to be related to its role in chromatin remodeling. Thus, this work adds a novel function to the chromatin remodeling class of proteins and should have a wide audience in the field of cell science. I have just a few points that need to be addressed before publication.

Answer: We sincerely thank this reviewer for his/her positive opinion on our manuscript as well as for all of his/her pertinent suggestions. We have successfully addressed all of them.

Major Comments

Comment#1: The authors state that DPF3 is involved in the "regulation of dynamic PCM1 distribution". This conclusion is based on IF observations (Figure 6D). The staining does not show the centrioles, so it is difficult to really determine how the satellites are distributed after DPF3 depletion. Could the authors quantify PCM1 around centrioles in control and DPF3 depleted cells? Could the authors also show that the overall expression of PCM1 is not affected, which would better support a role in satellite distribution or assembly?
Answer: Thank you for this suggestion. The accumulation of PCM1 in centriolar satellites was apparent in control-depleted cells, but it was considerably diminished upon DPF3 depletion. We have now quantified the signal intensity of PCM1 staining around the centrosome in both control and DPF3-depleted cells as previously published (PMID: 31023719).
To quantify PCM1 dispersion, a circle at the basis of cilia was drawn in control conditions (No si). In DPF3-depleted cells, the circle was place in the area where the signal is still brightest (*see* figure 1D in the revised version of our manuscript). The pixel density of anti-PCM1 staining in this area was measured using Image J software. Fluorescence intensity was quantified, and signal level in control cells was normalized to 1. Analysis of control cells and DPF3-depleted cells from three different experiments is presented in new Figure 7F of the revised manuscript. A decrease in integrated anti- PCM1 immunofluorescence intensities confirms PCM1 redistribution in DPF3-depleted cells.
In addition, we have analyzed the expression of PCM1 upon DPF3 depletion by western blot (new Figure 7G in the revised version of our manuscript). The expression of PCM1 is not affected in DPF3-depleted cells. Together these results suggesting that DPF3 is more likely involved in the regulation of centriolar satellite distribution/organization rather the assembly *per se*.

Comment#2: The authors clearly show that the axoneme does not grow in DPF3-depleted cells and conclude that "the ciliary vesicles dock to the plasma membrane as judged by the appearance of appendages". This sentence is unclear to me because distal appendages are formed before docking. In the EM images, only very small CVs are seen in DPF3-depleted cells, and none are actually docked to the TF. The CV does not seem to form properly, suggesting a very early defect in centriole to basal body conversion. The sections do not really allow to show that the centriole is normal as stated by the authors. Therefore, the conclusions must be qualified.
Answer: We agree with this reviewer's comment. We have modified the interpretation and conclusion of this EM experiment (*see* page 14 of our revised manuscript).

Minor Comments

Comment#3: Apparently a similar role of chromatin remodeling complexes at the mitotic apparatus has been recently described in addition to other cited references (Messina et al. BMC Biology (2022) 20:172 https://doi.org/10.1186/s12915-022-01365-5). The authors should discuss their results also in light of this study, in particular for researchers outside the chromatin field.
Answer: We missed this interesting publication and we apologize for that. We have now added this reference in the new version of our manuscript.

Comment#4: Figure 5A: the different classes of defects are not easy to see, for example it is not clear to me how the authors determine kinetochores with lateral attachment. A zoom on a

© 2024. Published by The Company of Biologists under the terms of the Creative Commons Attribution License (https://creativecommons.org/licenses/by/4.0/).

control kinetochore/KF attachment could be useful.
Answer: This reviewer is right. The conclusion on kinetochore with lateral attachment requires deeper investigation. We have therefore decided to only interpret and discuss cold-resistant K-fibers and Kinetochore-Microtubule (KT-MT) mis-attachments as published in several reports (PMID: 21397845, PMID: 28441529, PMID: 33322077, PMID: 34885726) Please note that this section has been entirely rephrased in the new version of our manuscript.

Comment#5: Line 261: the levels of pH (Ser10) already decrease 12h post release.
Answer: Thank you for this correction. We have mentioned this observation in the revised version of our manuscript.

Comment#6: Line 270: "altogether these data demonstrate that DPF3 ……, therefore maintaining genomic stability" this last part of the sentence is overstated here. This is only suggested in the next section.
Answer: We fully agree with this reviewer's suggestions. We have changed the conclusion Line 270.

Comment#7: Figure 2A: I do not see white arrows or arrowheads on the figure
Answer: We are sorry for this mistake. This has been corrected in the revised version of our manuscript.

Comment#8: Figure 6F (Change FT to TF).
Answer: This has been changed in the revised version of our manuscript.

Reviewer#3
The manuscript entitled "non-canonical role for the BAF complex subunit DPF3 in mitosis and ciliogenesis" by Verrillo et al. demonstrates new roles for the BAF chromatin remodelling complex subunit DPF3 in ciliogenesis and kinetochore- microtubule attachments, which have never been described before. The data provided are of good quality, well designed, explained and support the new roles proposed for the DPF3 protein. The manuscript is also well written and illustrated. I recommend it for publication when the authors clarify the points listed, provide some supplementary control experiments and correct some mistakes of cell cycle analysis in the manuscript.

Answer: We are pleased that the reviewer found an interest in our study and recommend it for publication in JCS. We thank this reviewer for her/his comments and suggestions. We have addressed all of them. We hope that our manuscript is now strengthened and can be accepted for publication in JCS.

Major Comments

Comment#1: Subcellular localization of DPF3 at the centrosome could be better characterized in combination with the centriole distal marker centrin and *bona fide* markers of the pericentriolar material cdk5rap2 or pericentrin, as PCM1 is a better marker of satellites. In multiciliated cells, centrin could also be used as an alternative to Plk4 staining.
Answer: This reviewer is right. We agree that PCM1 is centriolar satellite marker. We apologize for this mistake. We have reviewed the entire manuscript according to this comment.
Moreover, we have performed additional immunofluorescence experiments and high- resolution microscope analysis to better determine whether DPF3 was also present in the pericentriolar matrix and centrioles as well. As shown in Figure 1G and Figure S2 of the revised version of our manuscript, we observed that DPF3 does not co-localize with centrin (marker of centriole) and γ-tubulin (marker of pericentriolar matrix). However, we validate that DPF3 perfectly co-localizes with PCM1 centriolar satellite protein.

Regarding staining in multiciliated cells, we were limited by the number of different primary antibody species to perform triple immunofluorescence staining. We could not perform triple staining to detect DPF3 with axoneme marker (acetylated α-tubulin) and centrin due to limited availability of antibodies from different species working in IF experiment. We are aware that localization of DPF3 in multiciliated cells requires deeper characterization, which might be the scope of another study. We have therefore decided to remove those data and make more space for

© 2024. Published by The Company of Biologists under the terms of the Creative Commons Attribution License (https://creativecommons.org/licenses/by/4.0/).

additional data requested by the three reviewers

Comments #2 & #3: Perturbations of DPF3 function were performed after siRNA- based experiments. Rescue experiments are required to strengthen the specificity of the siRNA-based loss of function. In the whole manuscript, the authors could not discriminate between the two isoforms DPF3a and DPF3b in terms of sub-cellular localization and function because of the tools available. It is an important point to address as the two isoforms might be redundant or alternatively have distinct roles in ciliogenesis and/or kinetochore-microtubule attachments. One possibility would be to perform rescue experiments with the two isoforms separately to clarify this point.

Answer: We fully agree that rescue experiments would have been useful for confirming siRNA specificity but also to decipher which exogenously expressed DPF3 isoforms - DPF3a and/or DPF3b - could rescue the loss-of-function phenotype. However, we are actually facing technical challenge to address this question. Indeed, the overexpressed DPF3a or DPF3b cause formation of protein-like aggregates in eukaryotic cells (see Figure below). The maintenance of protein solubility is a fundamental aspect of cellular homeostasis. DPF3 aggregation might be associated with wide undesired/artifact cell phenotypes and therefore, cannot lead us to properly conclude about the effect of DPF3 re-expression

However, although the overexpression profile of DPF3 seems to be aberrant, a faint staining for DPF3a is observed with γ-tubulin, making us confident about the centrosomal localization of DPF3 in a more appropriated overexpressing system.

NOTE: We have removed unpublished data that had been provided for the referees in confidence.

To avoid erroneous conclusions when interpreting data from overexpressing experiment, we are actually developing a tetracycline-inducible gene expression system (TET-On system), which will afford tunable level of overexpressed DPF3 protein. Using this system we will re-express the siRNA-resistant wild type or deleted forms of DPF3 cDNA. This will help us not only to determine the importance of each isoform but also to identify which functional domains of DPF3a/b are responsible for the mitotic phenotype. These cellular models are currently under development and they will be used in a future project. Although we agree that rescue experiments might have been very informative, we believe that those rescue experiments are not highly mandatory for the first demonstration of a role for DPF3 in mitosis and ciliogenesis.

However, we have been able to address some specific role/difference for DPF3a and DPF3b using recombinant proteins. For instance, microtubule co-sedimentation and GST pull-down assays revealed that recombinant DPP3a protein and to a lesser extend recombinant DPF3b protein can bind polymerized microtubules but none of them interacts with monomer α-tubulin. These data have been added in the revised version of our manuscript (Supplemental Figures S3). Moreover, by GST pull-down assay we have also demonstrated that both DPF3a and DPF3b can interact with PLK1 and Aurora B kinases, which is consistent with the co-localization of DPF3 with CPC during mitosis (new Figures 3A and 3B in the revised version of our manuscript). Thanks to these new biochemical data, we bring at some points some evidences about the differences/similarities for DPF3a and DPF3b.

Comment#4: The authors cannot discriminate from the data provided in Figure 3 whether cells are blocked in G2 phase of the cell cycle and/or in mitosis. Therefore, they cannot conclude after Figure 3 that “DPF3 is required for mitotic progression and cell survival, therefore maintaining genomic stability” (lines 269-270). Indeed, it is the phosphorylation of cyclin B1 and not the level of expression per se which can be used as a marker of mitosis (Figure 3B). Cyclin B1 level is increasing from G2 phase onwards in cells. The same is true for histone 3 phosphorylation on Ser10, which starts in G2 phase of the cell cycle and accumulates in mitosis (Figure 3B). FACS analysis (Figure 3C and E) does not enable to discriminate between G2 and mitosis either. Aphidicolin treatment does not block cells in G1/S (line 251), but in S phase and is a source of DNA damage (Figure 3E). The authors should repeat the experiment using cdk4/6 inhibitors to block cells at the G1/S checkpoint without inducing DNA damage.

Answer: We fully agree that FACS analysis cannot discriminate between G2 and M phase. However, fluorescent live imaging analysis (Movies 3 to 8 in the revised version of manuscript) clearly demonstrated that DPF3-depleted cells are blocked at the metaphase-anaphase transition but not in early stages. New phase-contrast live imaging experiments (additional Movies 1 and 2 in the

© 2024. Published by The Company of Biologists under the terms of the Creative Commons Attribution License (https://creativecommons.org/licenses/by/4.0/).

revised version of our manuscript) also show that DPF3-depleted cells can reach mitosis. However, the cell division is aborted at this stage, consequently leading to cell death. Altogether these observations can assume that cell cycle progression of DPF3-depleted cells is blocked at the mitotic stage and not in G2 phase.
Finally, we have performed a new cell cycle analysis using the validated inhibitor of CDK4/cyclin D1, CAS 546102-60-7 (PMID: 12747775) as requested by this reviewer. FACS analysis showed that control cells can progress in the cell cycle 24 hours after the G1 block induced by CDK4 inhibitor, and accumulated in the next phase. However, a significant number of DPF3-depleted cells are not able to exit from mitosis and are still detected in M phase 24 hours after the G1/S release as similarly observed with aphidicolin. Due to space limitations the results of this new experiment are not presented in the revised version of our manuscript.

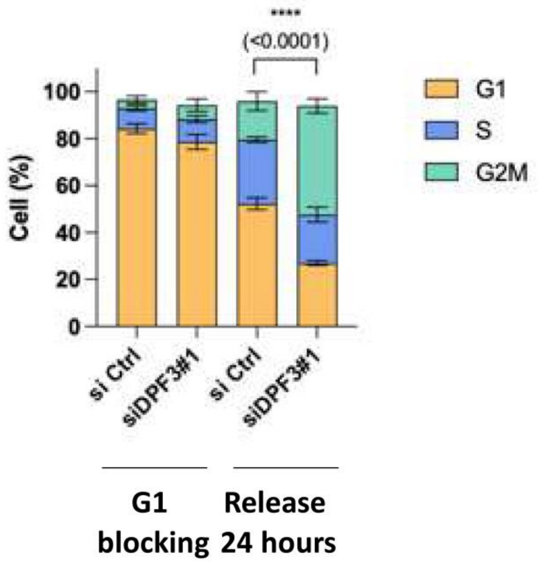

Comment#5: In the movies provided (movies 2 and 3), it seems that in a first-place chromosomes do not fail to align and even form a metaphase plate, but that in a second place only they start oscillating and leaving the metaphase plate. Can it be that it is the maintenance of kinetochore-microtubule attachments which is mainly impaired and not the establishment (as proposed in the main text, lines 276 or 308)?
Answer: The reviewer's is right. Metaphase plate seems to form correctly upon DPF3 depletion (Movies 4 to 8 in the revised version of our manuscript). However, we observed alignments with hyper-oscillating chromosomes. Consistent with the hyper- oscillating chromosomes, DPF3 depletion induced an increased fraction of cells harboring chromosome misalignment (quantification of experiments shown in Figures 5C, 5E and 5F in the revised manuscript). One consequence of hyper-oscillating chromosomes in DPF3-depleted cells is that they have a propensity to lose their attachment to the mitotic spindle (as evidenced by *Cold-stable microtubule assay')* and activate the spindle assembly checkpoint, thus explaining the mitotic delays we observed. Similar observations have been reported in the context of SF3B1 mutation (PMID: 37463047) or KIF18A depletion (PMID: 30122526); the later one was found within the bridging fiber area (PMID: 33480356) as similarly observed for DPF3.

Comment#6: In Figure 5A, it is difficult to appreciate whether cells after no si, siCtrl or siDPF3 are at the same stage of mitosis for analysis. One possibility would be to incubate cells with a MG132 inhibitor to favor the accumulation of cells in metaphase by preventing anaphase onset before the cold-depolymerization assay.
Answer: This is a valid point. This information was omitted in the first version of our manuscript and we sincerely apologize for that. This has been now added in the Material and Methods Section - '*Cold-stable microtubule assay*'

Minor Comments

Comment#7: The technique used to quantify is not always mentioned in the figure legend, like

© 2024. Published by The Company of Biologists under the terms of the Creative Commons Attribution License (https://creativecommons.org/licenses/by/4.0/).

in Figs 3A and 3G
Answer: We have described the FACS quantification method in the Material and Methods section - *'Cell cycle analysis' and 'Apoptosis analysis'*.

Comment#8: Abbreviations are not explained in Fig1A (CF, NSF, CSF)
Answer: We apologize for this lack of information. Here are the abbreviations for CF = Cytosolic fraction, NSF = Nuclear Soluble Fraction, CEF = Chromatin-enriched Fraction. These abbreviations have been also added in the Material & Methods section - *'Isolation of cytoplasmic, nucleoplasm and chromatin-enriched fractions'* as well as in the corresponding legend.

Comment#9: Material and methods section should include the procedures for quantifications (Figures 5B, 5D, 6E).
Answer: We have now included additional information for Figures 5B, 5D, 6E in the Material and Methods section - *'Cold-Stable microtubule assay'* .

For quantification in Figure 6, please note that we have performed an additional quantification as requested by this reviewer as well as Reviewer #2. Indeed, the accumulation of PCM1 in centriolar satellites was apparent in control-depleted cells, but it was considerably diminished upon DPF3 depletion. We have now quantified the signal intensity of PCM1 staining around the centrosome in both control and DPF3- depleted cells. To assess PCM1 dispersion, a circle at the basis of cilia was drawn in control condition (No si). In DPF3-depleted cells, the circle was place in the area where the signal is still brightest (see Figure 1D in the revised manuscript). The pixel density of anti-PCM1 staining in these areas was measured using Image J software. The signal level in control cells was normalized to 1. Analysis of control cells and DPF3-depleted cells from three different experiments is presented in new Figure 7F of the revised manuscript. A decrease in integrated anti-PCM1 immunofluorescence intensities confirms PCM1 redistribution in DPF3-depleted cells.
In addition, we have assessed the expression of PCM1 upon DPF3 depletion by western blot (new Figure 7G in the revised version of our manuscript). The expression of PCM1 is not affected in DPF3-depleted cells. Together these results suggesting that DPF3 is more likely involved in the regulation of centriolar satellite distribution/organization rather the assembly *per se*.

Comment#10: In figure 5A, kinetochore fibre width and density are not clearly visualized after only merged panels. The authors should provide in black and white microtubule staining as well for better visualization. They should also provide examples of normal kinetochore-microtubule attachments for comparison. High magnification panels are not necessarily clearly illustrating the defects spotted.
Answer: Unfortunately, due to some technical issues we have not been able to provide black and white tubulin staining. The conclusion on kinetochore with lateral attachment probably requires deeper investigation. We have therefore decided to only interpret and discuss cold-resistant K-fibers and Kinetochore-Microtubule (KT-MT) mis- attachments as published in several reports (PMID: 21397845, PMID: 28441529, PMID: 33322077, PMID: 34885726) Please note that this section has been entirely rephrased in the new version of our manuscript.

Comment#11: Kinetochore fibre width should also be quantified to better evaluate K fibre stability.
Answer: Quantification of K-fiber stability has been evaluated by measuring $\propto$-tubulin intensity as published in many publications (PMID: 32423652) (PMID: 31521166) (PMID: 35849559).
Since α-tubulin acetylation is considered as a marker of microtubule (MT) stability and mitotic K-fibers are acetylated (PMID: 34440628), the effect of DPF3 depletion on mitotic MT stability was determined by assessing this post-translational modification of α-tubulin. Western blot analysis was performed on soluble and insoluble tubulin fractions extracted from metaphase-arrested cells transfected or not with DPF3 siRNAs. As shown below, we observed a significant reduction of acetylated α-tubulin in the insoluble fraction of metaphase-arrested DPF3-depleted cells compared to control conditions. These data are preliminary and should be validated. They are part of another current DPF3 project in our lab, and will therefore not be included in the present manuscript.

NOTE: We have removed unpublished data that had been provided for the referees in confidence.

Comment#12: The discussion should be shortened and refocused on the potential function of DPF3 at the centrosome, in axoneme growth and in kinetochore- microtubule stability maintenance

© 2024. Published by The Company of Biologists under the terms of the Creative Commons Attribution License (https://creativecommons.org/licenses/by/4.0/).

instead of listing the pathologies related to cilia defects.
Answer: The discussion has been entirely reviewed. We have further discussed the role of DPF3 in mitotic structures as suggested by this reviewer.

Second decision letter

MS ID#: JOCES/2023/261744

MS TITLE: Non-canonical role for the BAF complex subunit DPF3 in mitosis and ciliogenesis

AUTHORS: Giulia Verrillo, Anna Maria Obeid, Alexia Genco, Jacopo Scrofani, Francois Orange, Sarah Hanache, Julien Mignon, Tanguy Leyder, Catherine Michaux, Celine Kempeneers, Noemie Bricmont, Stephanie Herkenne, Isabelle Vernos, Maud Martin, and Denis Mottet
ARTICLE TYPE: Research Article

We have now reached a decision on the above manuscript.

To see the reviewers' reports and a copy of this decision letter, please go to: https://submit-jcs.biologists.org and click on the 'Manuscripts with Decisions' queue in the Author Area. (Corresponding author only has access to reviews.)

As you will see, the reviewers gave favourable reports but raised some critical points that will require amendments to your manuscript. I hope that you will be able to carry these out because I would like to be able to accept your paper, depending on further comments from reviewers.

Please ensure that you clearly highlight all changes made in the revised manuscript. Please avoid using 'Tracked changes' in Word files as these are lost in PDF conversion.

I should be grateful if you would also provide a point-by-point response detailing how you have dealt with the points raised by the reviewers in the 'Response to Reviewers' box. Please attend to all of the reviewers' comments. If you do not agree with any of their criticisms or suggestions please explain clearly why this is so.

Reviewer 1

Advance summary and potential significance to field

Verrillo et al. here demonstrate interesting new roles for DPF3, a component of the BAF complex, in centrosome satellites and cilia, as well as in chromosome dynamics during mitosis. The authors have convincingly addressed the points that I raised in my initial review and I consider that the data presented now support the key conclusions drawn in the manuscript. I am enthusiastic about this study. It will be of interest to a range of readers in different areas of cell cycle biology.

Comments for the author

I have one remaining concern, which relates to points made in the previous round of review regarding the rescue controls. The authors explain clearly in their rebuttal why it was not technically feasible to perform robust rescue experiments with DPF3. This explanation should be included in the Results section, both to explain why this key control is not included and to help future workers in the area. A figure showing the aggregates might be included in Supplemental Data, depending on the authors' preferences, but one would not insist on that.

Reviewer 2

Advance summary and potential significance to field

© 2024. Published by The Company of Biologists under the terms of the Creative Commons Attribution License (https://creativecommons.org/licenses/by/4.0/).

In this study, Verillo et al., describe the unexpected function of the DPF3 component of the chromatin remodeling complex BAF in mitosis and ciliogenesis.

They show in cell culture that DPF3 is localized at different loci during mitosis and interphase in addition to the nucleus. They show that DPF3 localizes to centriolar satellites, the spindle midzone/bridging fiber area, and the midbody during mitosis. Using siRNA inactivation, they show that DPF3 is required for mitotic progression, chromosome alignment/distribution during mitosis, and cilium assembly in postmitotic cells. Taken together, the data are compelling and reveal a novel role for this protein that is unlikely to be related to its role in chromatin remodeling. Thus, this work adds a novel function to the chromatin remodeling class of proteins.

Comments for the author

The authors have responded positively to my previous requests. Furthermore, the authors have provided additional observations using higher resolution imaging and more markers to better define the cellular compartments where DPF3 localises. They also added some biochemical assays: WB, GST pull-down and co-sedimentation assays, which together reinforce their observations and their main conclusion about the function of DPF3 in mitosis and ciliogenesis.
I have no further requests or comments prior to publication.

Reviewer 3

Advance summary and potential significance to field

The authors propose for the first time new functions in ciliogenesis and mitosis of the BAF complex subunit DPF3. They provide convincing evidence of a centriolar satellite localization of the protein. They also show that the depletion of DPF3 in human cell lines is sufficient to impair the growth of axonemes in interphase and perturb spindle formation and function in mitosis that lead to cell death. This work will be of interest for the cell biology community working on the microtubule cytoskeleton.

Comments for the author

I acknowledge the efforts made by the authors to clarify the localization of DPF3 and to provide better quality images. They have now addressed my questions regarding the sub-cellular localization of DPF3 and the data provided in the manuscript now support a centriolar satellite localization of the protein.
Nonetheless, I feel that the functional assays still require some control experiments and quantifications of some new data provided, as well as more explanations in the Material and Method sections for the analysis. I therefore consider that the manuscript should be further revised.

1) I still have concerns regarding the function of DPF3. The title of the manuscript claims novel functions for DPF3 in ciliogenesis and mitosis based on functional assays relying only on siRNA approaches, but the authors do not provide any rescue experiments, which I consider as mandatory to validate such a claim. Even if I understand that the authors face technical issues, the tetracyclin-inducible gene expression system to tune the level of DPF3 that they present should solve their problem and results included in the present manuscript.

2) I was concerned by the fact that the authors cannot discriminate between a block in G2 or M phase. The new phase-contrast movies 1 and 2 provided clearly show that cells enter mitosis and die before dividing after DPF3 siRNA as compared to the control. Nonetheless, can the authors quantify the proportion of cells entering mitosis and dying before cell division in control and DPF3 siRNA conditions? It seems from the movie provided that this mitotic block and death phenotypes are more obvious at the end of the movie. It is the case, and why? Can the authors provide a timer on the movies?

© 2024. Published by The Company of Biologists under the terms of the Creative Commons Attribution License (https://creativecommons.org/licenses/by/4.0/).

3) The use of the CDK4/cyclin D1 inhibitor to synchronize cells at the G1/S transition is very convincing and should be included in the manuscript, instead of the aphidicolin experiment in Figure 4E, in case of space limitation. Indeed, aphidicolin treatment does not block cells at the G1/S transition (this should be corrected in the text lines 260 and 262) but impair S phase progression while inducing DNA damage able to activate the checkpoint at the G2/M transition. Therefore, DPF3 siRNA cells could be blocked at the G2/M transition before entering M phase as a result of the aphidicolin treatment. This experiment does not fuel the mitotic block observed after DPF3 siRNA but adds another level of complexity.

4) “the beginning of mitosis was confirmed by an increase of phosphorylated histone H3 on Serine 10…” (line 269). This phosphorylation starts at the end of G2 phase, so can the authors reformulate this sentence, as accumulation of cells at the end of G2 would also lead to an increase in PH3-S10P?

5) The authors explain very well in the rebuttal letter how they proceed with the quantifications of PCM dispersion in Fig7F. Can they add these explanations in the Mat et Met section of the manuscript?

Second revision

<u>Author response to reviewers' comments</u>

Answers to reviewer’s comments are in blue

Reviewer#1
Verrillo et al. here demonstrate interesting new roles for DPF3, a component of the BAF complex, in centrosome satellites and cilia, as well as in chromosome dynamics during mitosis. The authors have convincingly addressed the points that I raised in my initial review and I consider that the data presented now support the key conclusions drawn in the manuscript. I am enthusiastic about this study. It will be of interest to a range of readers in different areas of cell cycle biology.
<u>Answer</u>: We sincerely thank this reviewer for his/her positive opinion on our manuscript. We are very happy that he/she is enthusiastic about our work and considers our work of interest for different areas of research

<u>Comment#1</u>
I have one remaining concern, which relates to points made in the previous round of review regarding the rescue controls. The authors explain clearly in their rebuttal why it was not technically feasible to perform robust rescue experiments with DPF3. This explanation should be included in the Results section, both to explain why this key control is not included and to help future workers in the area. A figure showing the aggregates might be included in Supplemental Data, depending on the authors’ preferences, but one would not insist on that
<u>Answer</u>: We thank this reviewer for her/his comments. As requested by this reviewer we have now mentioned in our manuscript that we have tried rescue experiments and explained the technical challenges we faced with overexpressing DPF3 system. Sata showing the aggregates is shown in Figure S4C.

Reviewer#2

In this study, Verrillo et al., describe the unexpected function of the DPF3 component of the chromatin remodeling complex BAF in mitosis and ciliogenesis.

They show in cell culture that DPF3 is localized at different loci during mitosis and interphase in addition to the nucleus. They show that DPF3 localizes to centriolar satellites, the spindle midzone/bridging fiber area, and the midbody during mitosis. Using siRNA inactivation, they show that DPF3 is required for mitotic progression, chromosome alignment/distribution during mitosis, and cilium assembly in postmitotic cells. Taken together, the data are compelling and reveal a novel role for this protein that is unlikely to be related to its role in chromatin remodeling. Thus,

© 2024. Published by The Company of Biologists under the terms of the Creative Commons Attribution License (https://creativecommons.org/licenses/by/4.0/).

this work adds a novel function to the chromatin remodeling class of proteins.

The authors have responded positively to my previous requests. Furthermore, the authors have provided additional observations using higher resolution imaging and more markers to better define the cellular compartments where DPF3 localises. They also added some biochemical assays: WB, GST pull-down and co-sedimentation assays, which together reinforce their observations and their main conclusion about the function of DPF3 in mitosis and ciliogenesis. I have no further requests or comments prior to publication.

Answer: We sincerely thank this reviewer for his/her positive opinion on our manuscript. We are delighted that he/she considers our work now acceptable for publication in JCS.

Reviewer#3

The authors propose for the first time new functions in ciliogenesis and mitosis of the BAF complex subunit DPF3. They provide convincing evidence of a centriolar satellite localization of the protein. They also show that the depletion of DPF3 in human cell lines is sufficient to impair the growth of axonemes in interphase and perturb spindle formation and function in mitosis that lead to cell death. This work will be of interest for the cell biology community working on the microtubule cytoskeleton.

I acknowledge the efforts made by the authors to clarify the localization of DPF3 and to provide better quality images. They have now addressed my questions regarding the sub-cellular localization of DPF3 and the data provided in the manuscript now support a centriolar satellite localization of the protein. Nonetheless, I feel that the functional assays still require some control experiments and quantifications of some new data provided, as well as more explanations in the Material and Method sections for the analysis. I therefore consider that the manuscript should be further revised.

Comment#1: I still have concerns regarding the function of DPF3. The title of the manuscript claims novel functions for DPF3 in ciliogenesis and mitosis based on functional assays relying only on siRNA approaches, but the authors do not provide any rescue experiments, which I consider as mandatory to validate such a claim. Even if I understand that the authors face technical issues, the tetracyclin-inducible gene expression system to tune the level of DPF3 that they present should solve their problem and results included in the present manuscript.
Answer: We are sorry and surprised by this reviewer's comment. It seems that he/she does not judge our data sufficient enough to claim that DPF3 has a function in mitosis and ciliogenesis. However, we do believe that the precise localization of DPF3 in both mitotic and ciliated cells as well as multiple functional assays shown in the manuscript give different and complementary experimental evidences to support such conclusion. As also typified by reviewer#2: ' *the authors have further provided additional biochemical assays (GST pull-down, WB and microtubule co-sedimentation assays) in the last round of revision, which together reinforce their observations and their main conclusion about the function of DPF3 in mitosis and ciliogenesis'.*

Regarding rescue experiments, we would have love to provide data from such experiment. Reviewer#3 initially asked for these rescue experiments to strengthen the specificity of the siRNA-based loss of function. First, we would like to remind that several key experiments have been done with two different siRNA against DPF3 as well as with two negative controls, which make us confident about the specificity of the cell phenotype observed using siRNA-based approach. As explained in the previous round of review, overexpression of DPF3a/b isoforms in eukaryotic cells leads to aggregates which might be associated with undesired/artefactual cell phenotypes such as cell death, and therefore cannot lead us to properly conclude about the effect of DPF3 re- expression. A tetracycline-inducible gene expression system was proposed in the rebuttal letter but this approach is also hampered by various technical difficulties. In fact, endogenous DPF3 is expressed at very low level in many cells and, even though a Tet- On system is supposed to fine-tune the expression of protein, this is impossible to find the appropriate balance between the depletion of endogenous DPF3 and re-expression of exogenous DPF3 without aggregation properties. Moreover, we would like to emphasize that there is also another layer of complexity in the rescue experiment. Indeed, we have to consider to correctly co-express both DPF3a et DPF3b isoforms since the two isoforms seems to have combined /complementary role during mitosis

© 2024. Published by The Company of Biologists under the terms of the Creative Commons Attribution License (https://creativecommons.org/licenses/by/4.0/).

according to new data provide in the previous round of review. The requirement of co- expression further increases the technical challenge to perform adequate rescue experiment.

We are actually trying to develop a CRISPR/Cas 9 based-strategy to generate an efficient gene knockout-rescue system. However, this technical approach requires a precise and time-consuming development/optimization without any guarantee of success. So, in our case, rescue experiment is a very challenging task with multiple potential issues. Although they would have been informative, I hope that you share our scientific opinion that those experiments are not highly mandatory for the demonstration of a role for DPF3 in mitosis and ciliogenesis since we have used many others experiments in the paper to reach this conclusion.

As suggested by Reviewer#1, we propose to mention in our manuscript that we have tried rescue experiments and explained the technical challenges we faced with overexpressing DPF3 system. This will indeed explain to readers why this experiment is not included in the manuscript and will help future workers in the area. We sincerely prefer to consider this possibility rather than performing a ‘pushy’ and not well- established rescue experiment, which might lead to erroneous or inappropriate conclusion in our paper.

Hoping that you will find our scientific request justified and our work now suitable for publication in *Journal of Cell Science*

<u>Comment#2:</u> I was concerned by the fact that the authors cannot discriminate between a block in G2 or M phase. The new phase-contrast movies 1 and 2 provided clearly show that cells enter mitosis and die before dividing after DPF3 siRNA as compared to the control. Nonetheless, can the authors quantify the proportion of cells entering mitosis and dying before cell division in control and DPF3 siRNA conditions? It seems from the movie provided that this mitotic block and death phenotypes are more obvious at the end of the movie. It is the case, and why? Can the authors provide a timer on the movies?
<u>Answer</u>: It is true that phase-contrast live imaging experiments (Movies 1 and 2) clearly show that DPF3-depleted cells can reach mitosis. However, the cell division is aborted at this stage, consequently leading to cell death. These data validate initial fluorescent live imaging analysis (Movies 3 to 8), which in addition clearly demonstrated that DPF3- depleted cells are temporally blocked at the metaphase-anaphase transition and then die after mitotic cell division (Movie 8) .

Phase-contrast movies were set-up as following:

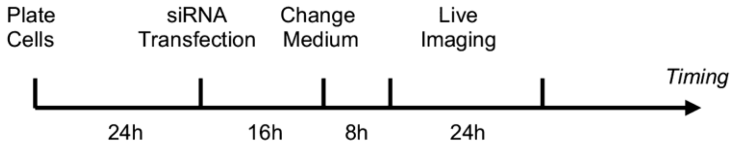

This information has been now added in the legend on these movies.

This experimental strategy was chosen: *i*) to let the siRNA works efficiently and adequately decrease the expression of DPF3 (total of 32 hours post-transfection) and *ii*) to perform live imaging during 24 hours (one image every 10 minutes). This timing ensures completion of at least one cell cycle and therefore led us to observe detect mitotic defects.Ò
Thus, the mitotic blockage and death phenotypes are more evident at the end of the movie because this occurs when DPF3 inhibition is adequate and after completion of one cell cycle.

<u>Comment#3:</u> The use of the CDK4/cyclin D1 inhibitor to synchronize cells at the G1/S transition is very convincing and should be included in the manuscript, instead of the aphidicolin experiment in Figure 4E, in case of space limitation. Indeed, aphidicolin treatment does not block cells at the G1/S transition (this should be corrected in the text lines 260 and 262) but impair S phase progression while inducing DNA damage able to activate the checkpoint at the G2/M transition. Therefore, DPF3 siRNA cells could be blocked at the G2/M transition before entering M phase as a result of the aphidicolin treatment. This experiment does not fuel the mitotic block observed after DPF3 siRNA but adds another level of complexity.
<u>Answer</u>: We have now corrected and mentioned that aphidicolin is an early S phase blocker. This

© 2024. Published by The Company of Biologists under the terms of the Creative Commons Attribution License (https://creativecommons.org/licenses/by/4.0/).

paragraph has been rephrased adequately. Please note that new data with CDK4/cyclin D1 inhibitor has been now added as Figure 4G a in the revised version of our manuscript as requested.

Comment#4: the beginning of mitosis was confirmed by an increase of phosphorylated histone H3 on Serine 10…" (line 269). This phosphorylation starts at the end of G2 phase, so can the authors reformulate this sentence, as accumulation of cells at the end of G2 would also lead to an increase in PH3-S10P?
Answer: We have now modified the corresponding section in the revised version of our manuscript. We have now mentioned that phospho-Histone H3 Ser10 is a late G2/M marker.

Comment#5: The authors explain very well in the rebuttal letter how they proceed with the quantifications of PCM dispersion in Fig 7F. Can they add these explanations in the Mat et Met section of the manuscript?
Answer: Good suggestion. This has been now added in the Mat and Meth - 'Immunofluorescence section'.

Third decision letter

MS ID#: JOCES/2023/261744

MS TITLE: Non-canonical role for the BAF complex subunit DPF3 in mitosis and ciliogenesis

AUTHORS: Giulia Verrillo, Anna Maria Obeid, Alexia Genco, Jacopo Scrofani, Francois Orange, Sarah Hanache, Julien Mignon, Tanguy Leyder, Catherine Michaux, Celine Kempeneers, Noemie Bricmont, Stephanie Herkenne, Isabelle Vernos, Maud Martin, and Denis Mottet
ARTICLE TYPE: Research Article

I am happy to tell you that your manuscript has been accepted for publication in Journal of Cell Science, pending standard ethics checks.

© 2024. Published by The Company of Biologists under the terms of the Creative Commons Attribution License (https://creativecommons.org/licenses/by/4.0/).
